# piRNAs safeguard splicing and RNA fidelity during heat shock

**DOI:** 10.1101/2025.10.27.684940

**Published:** 2025-10-28

**Authors:** Sehee Min, Johnny Cruz-Corchado, Veena Prahlad

**Affiliations:** Department of Cell Stress Biology, Roswell Park Comprehensive Cancer Center, Elm and Carlton Streets, Buffalo, NY 14263

**Keywords:** piRNA, heat shock, *hsp*, splicing, nonsense-mediated decay (NMD), endo-siRNAs, transcript fidelity, RNA quality control, protein homeostasis, *C. elegans*

## Abstract

Piwi-interacting RNAs (piRNAs) that silence transposons and other non-self-sequences in the genome, also pervasively target endogenous mRNAs in diverse species, and most germline transcripts in *Caenorhabditis elegans*^1–10^. The functions of this broad targeting remain unclear. Here, we uncovered a surprising role for the piRNA pathway in regulating the splicing and fidelity of nascent mRNAs induced during stress. Upon heat shock, piRNAs target essential heat-shock protein (*hsp*) RNAs—normally absent but massively upregulated upon stress—and generate abundant sequence-specific secondary RNAs (22G-RNAs) antisense to these transcripts. Instead of enforcing silencing, these 22G-RNAs associate with nascent *hsp* transcripts and RNA polymerase II at the transcription complex, delaying splicing and preserving a pool of pre-mRNAs for processing during recovery from stress. This role of the piRNA pathway in enforcing splicing delays, extends beyond *hsps* to alternatively spliced genes and ensures the proper expression of long-intron-containing genes in the genome. In piRNA-deficient *prde-1* mutants, splicing is precocious, *hsp* pre-mRNAs are depleted, expression of long-genes is impaired, transcripts accumulate errors, and consequently, embryos become critically dependent on nonsense-mediated decay (NMD) for survival. Downregulating NMD components in piRNA-deficient animals results in the accumulation of polyubiquitinated proteins and embryonic lethality. Thus, by safeguarding splicing under stress, the piRNA pathway complements NMD as a mechanism of RNA quality control, linking RNA fidelity to genome surveillance.

Piwi-interacting RNAs (piRNAs) are small, single-stranded RNA guides for PIWI Argonaute proteins that suppress transposable elements and foreign RNAs through transcriptional and post-transcriptional silencing^1–10^. Yet, across animal species, a large proportion of piRNAs are produced from, and target endogenous genes, suggesting that the piRNA pathway also plays evolutionarily ancient and conserved roles in regulating gene expression^1–7,11–15^. However, their endogenous functions remain mostly unknown. In *Caenorhabditis elegans*, piRNAs associate with PRG-1 (the *C. elegans* PIWI protein) and can target, with high mismatch tolerance, nearly all germline RNAs, including ectopic sequences such as GFP transgenes^1,2,16,17^. piRNA-targeting initiates the production of secondary antisense small endogenous silencing RNAs (endo-siRNAs, or 22G-RNAs) templated from the piRNA-targeted mRNAs by RNA-dependent RNA polymerases (RdRPs)^18–20^. These 22G-RNAs recruit WAGO Argonaute proteins to enforce co- and post-transcriptional silencing. Self-RNAs are spared this promiscuous silencing through their interactions with another Argonaute protein, CSR-1, guided by 22G-RNAs generated from actively translated transcripts independently of piRNAs^21,22^. These CSR-1 associated 22G-RNAs, thus, act as molecular engrams that recognize expressed ‘self’ genes to prevent their silencing. However, this leaves unexplained how inducible genes such as heat shock protein (*hsp*) genes—normally absent and thus lacking CSR-1–22G-RNA guides, but whose abrupt and massive expression is essential to ensure survival under heat shock and other stress^23–25^ — escape piRNA-mediated silencing.

## *hsp* mRNAs are targeted by the piRNA pathway

**Figure 1.**
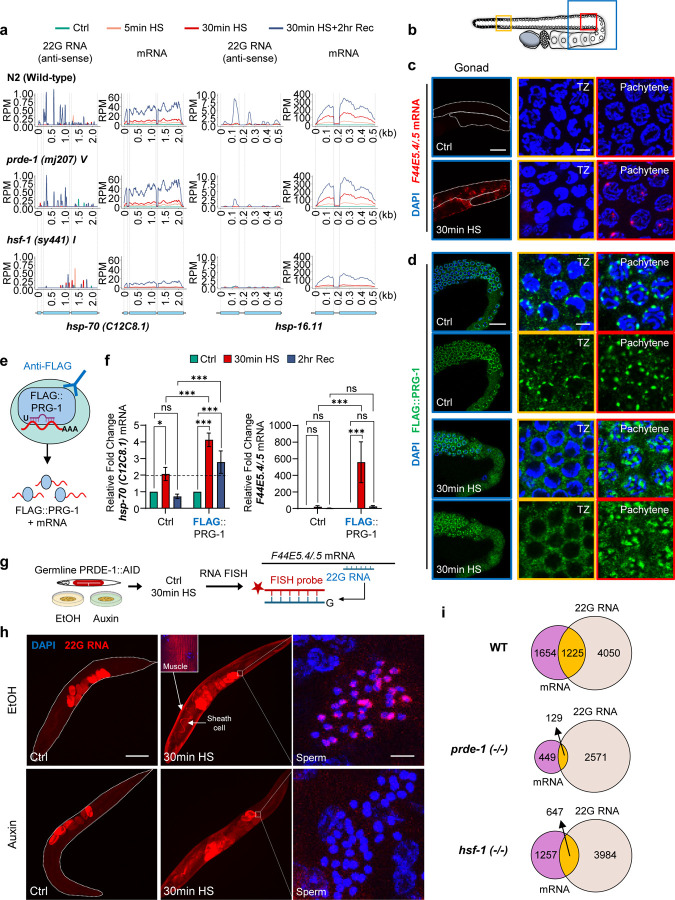
Piwi-interacting RNAs (piRNAs) target *hsp* mRNAs upon heat shock. **a.** Metaprofile plots showing normalized antisense 22G-RNA reads (RPM), and mRNA reads (RPM) across *hsp* mRNAs in wild-type (WT, *C. elegans* wild isolate, var Bristol), *prde-1(mj207) V,* and *hsf-1(sy441) I* mutants. **Top**: conditions of heat shock. **Bottom**: Schematic of *hsp* genes analyzed. **b-d.** Spatial localization of *hsp-70* mRNA and PIWI (PRG-1) in the WT germlines. **(b)** Schematic of gonad regions in **c** and **d**: Blue box, Gonad region shown. Orange box, transition zone (TZ). Red box, Late pachytene. Representative confocal images of each region showing **(c)** *hsp-70 (F44E5.4/.5)* mRNA localized using RNA Fluorescence *in situ* hybridization (RNA FISH; Projected image), and **(d)** PIWI (FLAG::PRG-1) by immunostaining with anti-FLAG antibody (Z-section). Germlines from non-heat-shocked (Ctrl) and heat-shock-exposed (30min HS) animals are shown. Red: *hsp-70 (F44E5.4/.5)* mRNA. Green: FLAG::PRG-1. Blue: DNA staining with DAPI. Scale bars, c: 50 μm (Gonad), and 3 μm (TZ, Pachytene). Scale bars, d: 20 μm (Gonad), and 3 μm (TZ, Pachytene). **e, f.** Native RNA immunoprecipitation (RIP) to probe *hsp* mRNA’s association with PIWI (FLAG::PRG-1). **(e)** Experiment Schematic. **(f)** Relative fold change (RT-qPCR) in *hsp-70s* (*C12C8.1* and *F44E5.4/.5*) mRNAs that immunoprecipitated with PIWI. *hsp* mRNA levels were normalized to *pmp-*3 (input) and are shown relative to non-heat-shocked animals. Ctrl: wild-type animals with no FLAG-tagged proteins were negative controls. *bath-45* served as positive control (Suppl. Fig 2b). Legend: Treatment conditions. Bars: mean value. Error bars: standard error. n = 3–5 biologically independent experiments. Statistical analysis: 2-way ANOVA. **g, h.** Germline depletion of PRDE-1 using the auxin-inducible degron (AID) system followed by RNA FISH to detect 22G-RNAs antisense to *hsp-70 (F44E5.4/.5).* **(g)** Experiment schematic: EtOH: Vehicle control. Probe design shown. **(h)** Representative micrographs (projected confocal Z-sections) showing RNA FISH of 22G-RNA under control conditions (EtOH) and upon PRDE-1 depletion (auxin). Red: 22G-RNA. Blue: DAPI stained DNA. Expression in muscle (arrow, box) and gonad sheath cells (arrow), and in sperm (highlighted). Embryos staining is non-specific. Scale bars: 100 μm (Ctrl, 30min HS), and 3 μm (Sperm). **i.** Venn diagrams showing overlap between differentially expressed mRNAs and 22G-RNAs upon 30 minutes heat shock in WT, *prde-1* and *hsf-1* mutants.

To address this question, we performed small RNA (sRNA-seq) and mRNA (paired-end, strand-specific, polyA-selected) sequencing from synchronized day-one adults exposed to mild (5 mins, 34°C) or strong (30 mins, 34°C) heat shock (Suppl. Fig 1a; Suppl. Tables 1, 2). Animals were also analyzed after two hours of recovery at 20°C, when normal physiological functions largely resume^26^. In parallel, to identify piRNA-dependent effects, we sequenced *prde-1* mutants, deficient in piRNA biogenesis^27,28^ (Suppl. Fig. 1a; Suppl. Tables 1, 2). As expected, heat shock induced global changes in mRNA (Suppl. Fig 1b), including the massive upregulation of *hsp* genes (Fig 1a; Suppl. Fig 1c, d *mRNA*). Surprisingly, in wild-type animals, heat shock also produced 22G-RNAs antisense to the *hsp* mRNAs (Fig 1a; Suppl. Fig 1c, d *22G-RNA*), suggesting that rather than escaping piRNA surveillance, *hsp* mRNAs may be targeted by the piRNA system. Like the *hsp* mRNAs, these antisense 22G-RNAs also appeared within 5 minutes of heat shock and peaked during recovery (Fig 1a; Suppl. Fig 1c, d *22G-RNA*). Consistently, by 30 minutes of heat shock, several piRNAs were also modestly but significantly induced (Suppl. Fig 1e, f). In *prde-1* mutants, *hsp* induction upon heat shock was intact, but many fewer 22G-RNAs antisense to *hsp* mRNAs were produced (Fig 1a; Suppl. Fig 1c, d *mRNA; 22G-RNA*), further suggesting that these 22G-RNAs were generated because of piRNA-targeting.

Because *hsp* expression is essential for stress survival, and piRNA targeting typically enforces silencing through the PIWI Argonaute PRG-1^16,19^, to confirm these unexpected results, we tested whether *hsp* mRNAs interact with PRG-1. PRG-1 and RdRPs reside in perinuclear condensates (nuage or germ granules) of the germline, where piRNA-induced 22G-RNAs are also produced^29,30^. Heat shock induced the expression of *hsp* mRNA in the germline: RNA Fluorescence *in situ* hybridization (RNA FISH) showed that *hsp-70s (C12C8.1* and *F44E5.4/.5)* were induced by (and only by) late pachytene nuclei (Fig 1b, c; Suppl. Fig 2a). PRG-1 was expressed in these regions. Moreover, although PRG-1 localization is known to dissolve upon exposure to increased temperatures, PRG-1 remained in perinuclear condensates upon heat shock, in these pachytene nuclei (Fig. 1b, d; compare transition zone, TZ, where *hsp* mRNAs are absent, with Pachytene). Immunoprecipitating PRG-1 under control, heat-shock, and recovery conditions from animals expressing FLAG-tagged PRG-1 showed that *hsp-70 (C12C8.1* and *F44E5.4/.5)* mRNAs associated with PRG-1 (RNA immunoprecipitation, RIP, followed by RT–qPCR; Fig 1e, f). Non-FLAG animals were used to account for non-specific interactions of *hsp* mRNAs, and *bath-45,* a known piRNA target, served as a positive control (Fig 1e, f; Suppl. Fig 2b). In addition, and most convincing, depleting PRDE-1 specifically in the germline using an auxin-inducible degron (AID) system abolished 22G-RNAs presence throughout the animal (RNA FISH), demonstrating that 22G-RNAs originate in the germline through piRNA targeting (Fig 1g, h; embryos show non-specific staining). Surprisingly, despite their germline origin, the 22G-RNAs were present throughout the animal, in somatic cells that expressed *hsp* mRNA (e.g. muscle, somatic sheath cells), as well as in the germline cells such as sperm, which do not (RNA FISH; Fig 1g, h; Suppl. Fig 2c). Upon germline depletion of PRDE-1, 22G-RNA levels were starkly decreased in somatic and germline cells (Fig 1g, h), while *hsp-70* mRNA continued to be expressed (Suppl. Fig 2c). Together, these findings confirmed that *hsp* mRNAs were subject to piRNA surveillance, actively targeted by the canonical piRNA pathway in the germline, and produced 22G-RNAs that propagated systemically across tissues.

Further evidence that the *hsp* genes induced by heat shock were templating antisense 22G-RNA production came from profiling mRNAs and sRNAs in a mutant in heat shock factor 1 (HSF1; HSF-1 in *C. elegans*), the transcription factor responsible for *hsp* expression upon heat shock. This mutant, *hsf-1 (sy441)* is deficient in heat-induced activity but expresses higher basal levels of *hsp* mRNA^26,31^ . Yet *hsf-1* mutants failed to produce 22G-RNAs antisense to *hsp* genes (Fig 1a; Suppl. Fig 1c, d). Surprisingly, even the few *hsp* mRNAs, like *hsp-70 (F44E5.4/.5)*, that did accumulate upon heat shock in *hsf-1 (sy441)* mutants, did not template 22G-RNAs (Suppl. Fig 1c, d), perhaps suggesting a broader role for HSF-1 in piRNA-dependent 22G-RNA production. piRNA surveillance also extended beyond *hsps* to other heat-shock-regulated genes: ~15% of differentially expressed genes (DEGs) upon a 5-minute heat-shock and ~40% by 30 minutes templated 22G-RNA production, and both the numbers of DEGs, and DEG-derived 22G-RNAs were reduced in *prde-1* and *hsf-1* mutants (Fig 1i; Suppl. Fig 2d; Suppl. Table 3). 22G-RNA function is determined by its Argonaute partner; however, the 22G-RNAs that targeted *hsp* mRNAs were ‘unclassified’ and not previously identified in association with known *C. elegans* Argonaute proteins (Suppl. Fig 3; Suppl. Table 3). However, DEGs other than the *hsp* genes, did template 22G-RNAs that had been previously identified as guide RNAs for WAGO, CSR-1 and other Argonaute proteins (Suppl. Table 4). WAGO-associated 22G-RNAs that were induced upon 30 minutes of heat shock exposure were piRNA-dependent, as apparent by their decreased levels in *prde-1* mutants, and in wild-type they increased as their target mRNAs decreased, consistent with their known roles in post-transcriptional or co-transcriptional silencing^8,32^ (Suppl. Fig 3a, b). CSR-1 22G-RNAs declined upon heat shock (perhaps due to translational attenuation), as their target mRNAs rose in both wild type and *prde-1* mutants, consistent with their mostly piRNA-independent production (Suppl. Fig 3a, b). Unclassified 22G-RNAs continued to increase in a *hsf- 1-* and *prde-1*- dependent manner, even during recovery from heat shock, as did their complementary mRNAs (Suppl. Fig 3a, b). The effects were dynamic and reversible during recovery from stress (Suppl. Fig 2d (Rec); Suppl. Fig 3a, b), and the mRNAs that templated 22G-RNA production showed distinct GO enrichments (Suppl. Fig 4; Suppl. Table 5), suggesting that the broad piRNA-dependent and independent 22G-RNA expression that was triggered by heat shock likely contributes to the pervasive transcriptome-wide remodeling^8,32^ universally associated with this stress-induced transcription program.

## The piRNA-pathway regulates post-transcriptional splicing.

**Figure 2.**
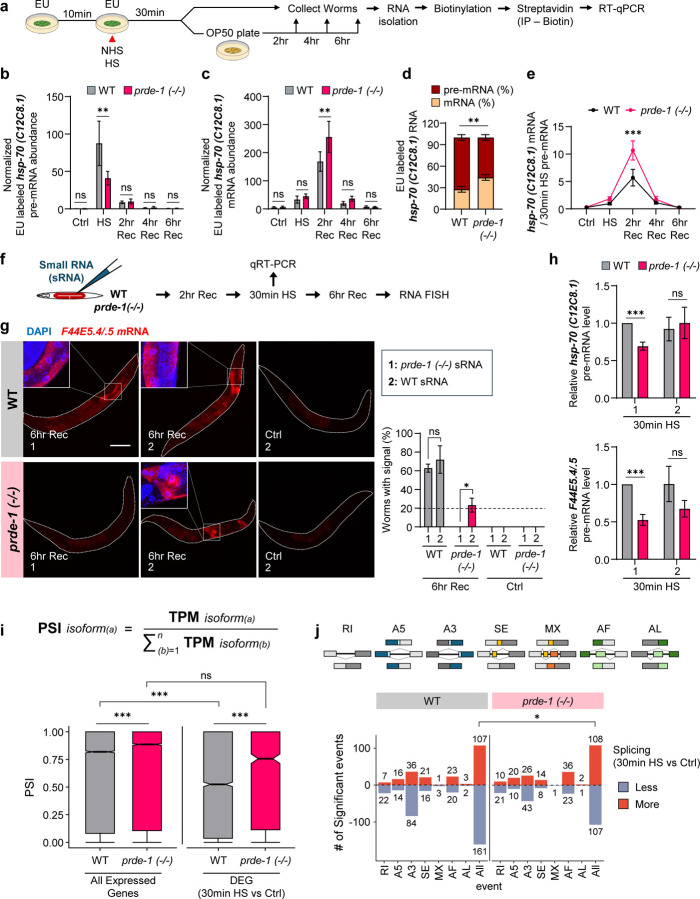
piRNAs delay *hsp* mRNAs splicing. **a-e.** Metabolic pulse-chase labeling with EU and quantification of *hsp* RNA. **(a)** Experiment schematic: animals were fed EU-mixed bacteria 10 minutes before heat shock (pre-loading), heat shocked for 30 minutes (pulse-labeling nascent RNA), and moved onto unlabeled bacterial plates (chase). The EU-labeled nascent RNA was isolated from NHS animals (Ctrl), heat-shocked animals immediately after 30 min heat-shock (HS), and after 2,4, and 6 hrs post-heat shock recovery (Rec), biotin-conjugated, and immunoprecipitated. Normalized absolute RNA abundance was quantified by RT-qPCR (see methods; *pmp-3* mRNA used to normalize for variability in labeling). EU-labeled *hsp-70 (C12C8.1)* **(b)** pre-mRNA and **(c)** mRNA abundance. Y-axis: Normalized abundance. X-axis: Treatments. Note EU-labeled mRNA is derived from the splicing of EU-labeled pre-mRNA. **(d)** Percentages of pre-mRNA and mRNA in total EU-labeled RNA after 30 min heat shock. **(e)** Relative amounts of EU-labeled spliced mRNAs produced from the nascent EU-labeled unspliced pre-mRNA. Values normalized to *pmp-3*. Y-axis: Relative abundance. X-axis: Treatment. Bars (b-d) and line graph (e): mean value. Error bars: standard error. (b, c, e) n = 5~13 biologically independent experiments. Statistical analysis: 2 way-ANOVA; (d) n = 14 biologically independent experiments. Statistical analysis: two-tailed unpaired t-test. **f, g.** Microinjection of small RNA fractions isolated from WT animals rescues *hsp-*70 fate. **(f)** Experiment schematic: small RNA fractions purified from heat-shocked WT and *prde-1* animals were injected into WT and *prde-1* animals shortly before heat-shock exposure, and animals were probed for *hsp-70 (F44E5.4/.5)* 6 hours post-heat shock. Non-heat-shock animals were injected to control for effects of injection alone. **(g)** RNA FISH of *hsp-70 (F44E5.4/.5)* in WT and *prde-1* animals 6 hours following heat shock or under NHS conditions. Injection mix (box) and treatment indicated. Red: *hsp-70 (F544E5.4/.5)* mRNA. Blue: DNA staining with DAPI. Scale bars: 100 μm. **Right:** Quantification of results. Y-axis: Percentage of injected animals positive for *hsp-70*. X-axis: Injection mix, strains and treatment. Bars: mean value. Error bars: standard error. n = 17~22 worms (6hr Rec), 6~10 worms (Ctrl). Statistical analysis: Fisher’s exact test. **h.** Microinjection of small RNA fractions isolated from heat-shocked WT animals rescues *hsp-*70 pre-mRNA levels. Normalized RT-qPCR of relative fold change of *hsp-70s* (*C12C8.1 and F44E5.4/.5*) pre-mRNAs following sRNA injection. Y-axis: *hsp-70* pre-mRNA levels, normalized to *pmp-3*, relative to heat-shocked wild-type animals injected with small RNAs from *prde-1* mutants. X-axis: Injection mix (see box in Fig 2g). Bars: mean value. Error bars: standard error. n = 6 biologically independent experiments. Statistical analysis: two-tailed unpaired t-test. **i.** Genes differentially regulated by heat shock in WT, but not *prde-1* animals, are enriched for alternative spliced isoforms. **Top**: Calculation of PSI “percent spliced in” values. **Bottom**: Alternative spliced isoforms, as reflected by PSI values, in all expressed genes, and in differentially expressed genes (p.adj <0.05) in wild-type and *prde-1* animals. Boxplots: median value depicted. Error bars: standard error. Statistical analysis: two-tailed unpaired t-test. **j.** Alternative splicing (AS) events in WT and *prde-1* animals upon heat shock. **Top**: AS events, retained intron (RI), alternative 5’/3’ Splice Sites (A5/A3), skipped exon (SE), mutually exclusive exons (MX), and alternative first and last exons (AF/AL). **Bottom**: Number of AS events amongst differentially expressed genes (HS vs NHS condition; p.adj <0.05) in WT and *prde-1* mutants. Bars: Number of events (splicing increase or decrease relative to NHS condition). Statistical analysis: Chi-square analysis.

Given the known roles of piRNAs in post- and co-transcriptional silencing, we suspected that piRNA targeting of *hsp* and other stress-induced genes functioned to clear transcripts during recovery from heat shock. If so, *hsp* mRNAs should persist longer after stress in *prde-1* mutants. Instead, RT-qPCR (Suppl. Fig 5a–c) and RNA-FISH (Suppl. Fig 5d) revealed the opposite: the steady state abundance of *hsp* mRNAs declined more rapidly in *prde-1* mutants compared to wild type. We examined whether decreased RNA Polymerase II (Pol II) binding at *hsp* genes^33^, or altered transcription duration, explained this progressive decline. But this was also not the case: chromatin immunoprecipitation followed by qPCR (ChIP-qPCR) showed that Pol II levels at *hsp* gene transcription start sites (TSS), and its return to basal levels by 2 hours post-heat shock, were similar in *prde-1* and wild-type animals (Suppl. Fig 5e, f). Therefore, to understand the role of the piRNA pathway’s targeting of *hsps* and other heat-shock genes, we directly assessed *hsp* mRNA dynamics in the absence and presence of the piRNA pathway by its synthesis and turnover using a pulse-chase metabolic labeling approach: we labeled the heat-shock induced pre-mRNA with 5-ethynyluridine (EU)^34,35^ provided to the animals only during heat shock exposure, and tracked the fate of this RNA during recovery from heat shock by absolute RT–qPCR using exon–intron (pre-mRNA) and exon–exon (mature mRNA) primers (Fig 2a; see methods for experimental details). Consistent with similar Pol II occupancy at *hsp* genes, *prde-1* synthesized similar total amounts of RNA as wild-type animals upon heat shock (Suppl. Fig 5g, h). Despite this, *prde-1* mutants had significantly reduced amounts of nascent (EU-labeled) pre-mRNA by the end of the 30-minute heat shock (Fig 2b; Suppl. Fig 5i), but, on the other hand, accumulated significantly more EU-containing spliced mRNA by 2 hours following heat shock (Fig 2c; Suppl. Fig 5j). This suggested that in the absence of piRNAs, *hsp* splicing or maturation occurred at an accelerated pace, resulting in a precocious increase in nascent mRNAs and a concomitant decrease in pre-mRNA pools.

The amounts of unspliced nascent pre-mRNA present at the end of heat shock exposure, calculated as the ratio of EU-labeled pre-mRNA over total EU-labeled RNA^36^, also indicated that piRNA/22G-RNAs functioned to delay splicing in wild-type animals. In the presence of piRNAs/22G-RNAs, in wild-type animals, ~75% of nascent *hsp-70 (C12C8.1)* transcripts, which contain two introns, remained as pre-mRNA to be spliced post-transcriptionally during recovery (Fig 2d). In contrast, only ~57% persisted unspliced in *prde-1* mutants (Fig 2d), suggesting that the piRNA pathway was biasing the decision between co- and post-transcriptional splicing to favor delayed splicing. This remaining *hsp-70 (C12C8.1)* was also spliced more rapidly in *prde-1* animals: within 2 hours of recovery, dramatically more EU-containing spliced mRNAs were produced from the nascent EU-labeled unspliced pre-mRNA (Fig 2e). The other inducible *hsp-70 (F44E5.4/.5)*, contained only one intron, and was mainly co-transcriptionally spliced in both wild-type and *prde-1* animals (Suppl. Fig 5k). Still, the post-transcriptional splicing of the residual *hsp-70 (F44E5.4/.5)* proceeded markedly faster in *prde-1* mutants than in wild-type animals (Suppl. Fig 5l). This was also true for the small heat shock protein (*hsp-16.11*; Suppl. Fig 5m).

The accelerated splicing rates in *prde-1* mutants were not simply due to altered expression of protein coding genes. We could rescue *hsp* RNA dynamics — both the persistence of *hsp* mRNA (Fig 2g), and the levels of *hsp* pre-mRNA in heat-shocked *prde-1* animals (Fig 2h) by microinjecting *prde-1* animals with only the heat-shock-induced small RNA fraction (<200nt) obtained from wild-type, but not *prde-1* animals, shortly prior to heat shock exposure. Small RNA injections *per* se did not induce or enhance *hsp* expression (Fig 2g, h). Thus, global alterations in mRNAs were unlikely to be responsible for the differences in splicing rates in *prde-1* animals, and, together with the results from pulse-chase metabolic labeling, these results showed that the piRNA/22G-RNA pathway functioned dynamically, preserving mRNA expression during heat-shock recovery by restraining splicing and maintaining a pool of pre-mRNAs for delayed processing.

Heat shock and other stressors lead to genome-wide splicing inhibition, intron retention, and retention of unspliced RNAs in chromatin ^36–40^. We therefore analyzed our RNA-seq data to assess whether the piRNA pathway was responsible for intron retention or alternative splicing (AS) of genes other than *hsps*, leveraging previous studies demonstrating introns in poly(A)+ selected mRNAs^36,41,42^. In wild-type animals, heat shock-DEGs (p.adj <0.05) were enriched for AS isoforms, and had significantly more transcript isoforms than DEGs in *prde-1,* as indicated by their much lower “percent spliced in” (PSI or Ψ)^43,44^ values (Fig 2i). In addition, 540 transcripts showed significant dPSI (differential PSI; p <0.1) values, compared to only 139 transcripts in *prde-1* (Suppl. Table 6). Moreover, for seven representative AS events^45,46^ — retained intron (RI), alternative 5’/3’ Splice Sites (A5/A3), skipped exon (SE), mutually exclusive exons (MX), and alternative first and last exons (AF/AL) (Fig 2j) — mRNAs differentially expressed upon heat shock in wild-type animals were significantly less spliced, retaining more introns compared to *prde-1* (Fig 2j; Suppl. Table 7). Nearly 40% (125/308) of these genes that produced AS transcripts upon heat shock in wild-type animals templated 22G-RNAs, compared to only 12% (35/280) in *prde-1* (Suppl. Fig 6a). These AS mRNAs were enriched in developmental processes, and in particular in neuronal functions known to rely on AS (Suppl. Fig 6b; Suppl. Table 8), suggesting that their delayed splicing may be important for the restoration of developmental processes during recovery from heat shock, and accordingly, heat-shocked *prde-1* mothers showed a marked delay in generating viable embryos post-heat shock (Suppl. Fig 6c, d).

## 22G-RNAs act through a ‘telescripting’-like mechanism to protect long gene expression.

Mechanistically piRNA/22G-RNAs could regulate splicing indirectly, through their known interactions with chromatin^8,29,47^. Alternatively, and consistent with the rapid induction and splicing of *hsp* genes, they could regulate splicing directly by acting on target pre-mRNAs at the transcription complex to bias splicing decisions^39^. Therefore, to understand how 22G-RNAs regulate splicing, we adapted Photoactivatable Ribonucleoside-Enhanced Crosslinking and Immunoprecipitation (PAR-CLIP) to test whether these small RNAs are associated with Pol II at the transcription complex. While co-transcriptional splicing occurs in association with elongating Pol II, post-transcriptional splicing is thought to occur after the pre-mRNA is released from Pol II, but the mechanisms that regulate the timing, transition, and completion of splicing are unclear^48,49^ . Therefore, taking advantage of the fact that 4sU, like EU, is primarily incorporated into nascent RNAs, we also assessed if *hsp* mRNA and/or pre-mRNA associated with Pol II, by immunoprecipitating Pol II, immediately at 30 minutes upon heat shock, when transcription was still in process, or after 2 hours of recovery from heat shock, when Pol II was no longer engaged with the *hsp* locus (ChIP-qPCR; Suppl. Fig 5e, f), but spliced mRNAs accumulated (Fig 2c; Suppl. Fig 5j).

**Figure 3.**
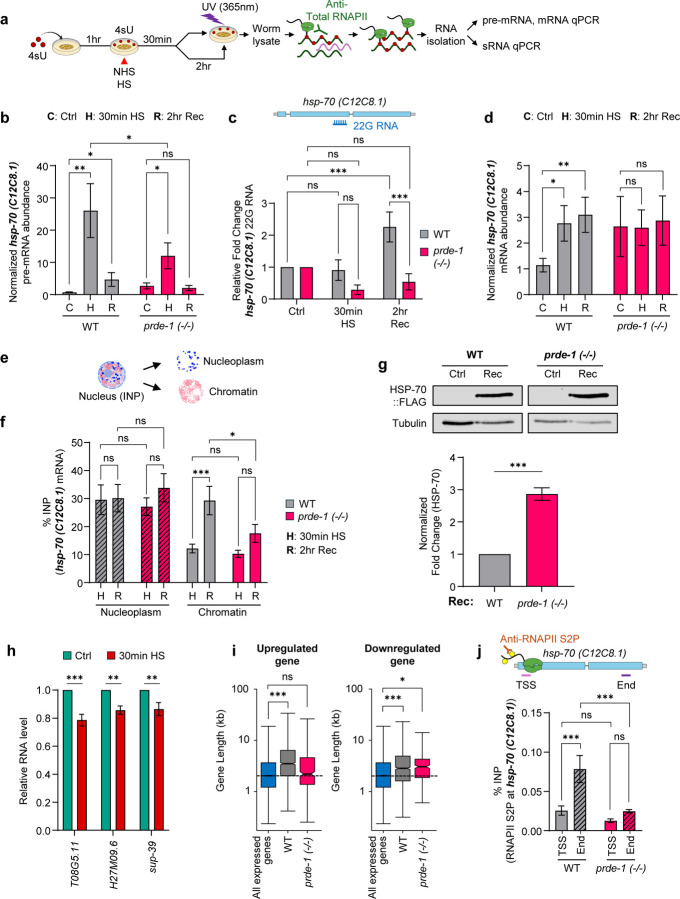
Antisense 22G-RNAs associated with RNA Polymerase II at the transcription complex to modulate post-transcriptional splicing. **a-d.** Modified PAR-CLIP to assess 22G-RNAs’ role in splicing. **(a)** Experiment schematic: Animals were fed 4sU-mixed bacteria, subjected to heat shock, exposed to UV (365 nm) either immediately or after 2 hours of recovery, RNA Pol II was immunoprecipitated, *hsp* mRNA and pre-mRNA were quantified by computing absolute RNA abundance (RT-qPCR and standard curves; *pmp-3* mRNA used to normalize for variability in IP amounts across samples and experiments) and 22G-RNA quantified as relative fold change using stem loop PCR (design shown). See Suppl. Fig 7c for details. Amounts of **(b)** *hsp-70 (C12C8.1)* pre-mRNA, **(c)** 22G-RNA, and **(d)** *hsp-70 (C12C8.1)* mRNA that cross-linked to, and immunoprecipitated with Pol II. Y-axis: Normalized or relative abundance. X-axis: Treatment conditions (see top legend). Bars: mean value. Error bars: standard error. (b) n = 8~13 biologically independent experiments. Statistical analysis: two-tailed unpaired t-test (within strain) and 2-way ANOVA (between strains); (c) n = 5~6 biologically independent experiments. Statistical analysis: 2-way ANOVA; (d) n = 5~10 biologically independent experiments. Statistical analysis: 2-way ANOVA. **e, f.** Nuclear fractionation to assess chromatin retention of *hsp-70* RNA. **(e)** Experiment schematic. Nuclei were isolated, fractionated to obtain nucleoplasm and chromatin, and enrichment of *hsp-70* mRNA in both fractions was determined. **(f)** Fold enrichment over input (nuclei) of *hsp-70 (C12C8.1)* mRNA in respective fractions. Y-axis: % input (total nucleus). X-axis: Nuclear fraction, strain, and treatment. Bars: mean value. Error bars: standard error. n = 9~10 biologically independent experiments. Statistical analysis: 2-way ANOVA. **g.** HSP-70 protein levels in WT and *prde-1.* **Top:** Representative western blot image showing HSP-70(C12C8.1)::FLAG in NHS (Ctrl) or after heat-shock in WT and *prde-1* animals expressing endogenous *flag*-tagged *hsp-70*. Animals harvested 3 hrs following HS (Rec) to allow HSP-70 protein synthesis. Tubulin: loading control. **Bottom:** Relative HSP-70 protein expression (relative to wild-type heat-shocked levels, because control animals do not express detectable HSP-70 protein). Bars: mean value. Error bars: standard error. n = 3 biologically independent experiments. Statistical analysis: two-tailed unpaired t-test. **h.** U1 RNA levels upon heat shock in WT animals (RT-qPCR: relative fold change). U1 RNA values normalized to *pmp-3* mRNA values and shown relative to NHS levels. X-axis: U1 RNAs. Bars: mean value. Error bars: standard error. n = 3~4 biologically independent experiments. Statistical analysis: 2-way ANOVA. **i.** Gene length dependence of heat-shock upregulated and downregulated protein-coding genes genes (p.adj <0.05). Y-axis: Average gene lengths (kb). X-axis: Strain. Genome-wide gene lengths of all expressed genes depicted for comparison. Boxplots: median value depicted. Error bars: standard error. Statistical analysis: Tukey HSD. **j.** Elongating RNA polymerase II (anti-S2P) levels at *hsp* gene. **Top:** Schematic of *hsp-70 (C12C8.1)* gene, and regions probed: Transcription Start Site (TSS) and distal regions (3’) of *hsp-70* (*C12C8.1*). **Bottom:** ChIP-qPCR showing fold enrichment over input. Y-axis: % ChIP input. X-axis: Strain, and region assayed. Bars: mean value. Error bars: standard error. n = 4~8 biologically independent experiments. Statistical analysis: 2-way ANOVA.

Animals were fed 4-thiouridine (4sU) at non-inhibitory concentrations (Fig 3a; Suppl. Fig 7a, b), subjected to heat shock, exposed to UV (365 nm) to crosslink nascent RNA to Pol II at the described time points, Pol II was immunoprecipitated, and the RNA species associated with Pol II identified using absolute and stem-loop RT-qPCR. In wild-type animals, as expected, unspliced *hsp* pre-mRNAs co-immunoprecipitated with Pol II at 30 minutes after heat shock, decreasing by 2 hours post-heat shock recovery (Fig 3b; Suppl. Fig 7d). Nevertheless, a small but significant fraction of nascent pre-mRNA remained associated with Pol II 2 hours post-heat shock (Fig 3b; Suppl. Fig 7d), when Pol II was no longer transcriptionally engaged (ChIP-qPCR; Suppl. Fig 5e, f). Notably, 22G-RNAs antisense to *hsp-70* (*C12C8.1*) also co-immunoprecipitated with Pol II, 2 hours post-heat shock (Fig 3c), as concomitantly, increasing amounts of *hsp-70* mRNA were spliced, and also associated with Pol II (Fig 3d). Furthermore, chromatin fractionation showed that these progressively increasing spliced mRNAs were retained in chromatin (Fig 3e, f). In *prde-1* mutants, less pre-mRNA (Fig 3b), but more constitutively spliced nascent mRNA (Fig 3d) was associated with Pol II at 30 minutes heat shock, and 22G-RNAs were not present (Fig 3c). In addition, spliced *hsp-70* mRNA failed to increase on chromatin (Fig 3e, f), or in association with Pol II above these basal constitutive levels (Fig 3d), instead appearing to be efficiently exported and translated as seen by elevated HSP-70 protein levels (Fig 3g).

A broadly similar dynamic was observed with *hsp-70 (F44E5.4/.5)*, even though the majority of RNA was already spliced, co-transcriptionally (Suppl. Fig 5k). Here, by the end of 30 minutes of heat shock, the remaining amounts of pre-mRNA that associated with Pol II were not significantly different between wild-type and *prde-1* animals (Suppl. Fig 7d), but significantly more mRNA was crosslinked with Pol II in wild-type animals (Suppl. Fig 7e), remained associated with Pol II during recovery (Suppl. Fig 7e), and accumulated in chromatin (Suppl. Fig 7f). Also, like with *hsp-70 (C12C8.1)*, significantly more 22G-RNA associated with Pol II in wild-type animals compared to *prde-1* (Suppl. Fig 7g). Perhaps due to the difference in splicing strategy between the two *hsp-70s*, 22G-RNA kinetics in association with Pol II varied: at 30 minutes upon heat shock, 22G-RNA associated with Pol II decreased in *prde-1*, rather than increasing in wild-type. Notwithstanding these differences, these data together showed that the piRNA-generated 22G-RNAs act at the transcription complex, associating with Pol II, to delay splicing, and promote chromatin retention of *hsp* mRNAs, postponing the availability of *hsp-70* mRNAs for translation. The continued association of unspliced and spliced RNAs along with 22G-RNAs with Pol II after transcription was complete and Pol II had disengaged from the *hsp* genes — most evident in *hsp-70 (C12C8.1)*, where a larger fraction underwent post-transcriptional splicing — also suggested that post-transcriptional splicing of *hsp* RNA occurs in direct or indirect association with Pol II.

The ability of antisense 22G-RNAs to retard splicing at the transcription complex through association with Pol II and nascent pre-mRNA was reminiscent of mechanisms by which U1 snRNA base-pairs with nascent transcripts to modulate their splicing efficiency and rates, and initiates telescripting, a process that prevents premature transcription termination, particularly of long-intron containing genes^50–52^. In mammalian cells, heat shock decreases U1 snRNA levels, resulting in the loss of telescripting and a decrease in the expression of long-intron-containing genes^53,54^. Like mammalian cells, *C. elegans*, also showed a modest but significant decrease in at least three of five U1 snRNAs tested (Fig 3h; Suppl. Fig 7h). Yet, unlike mammalian cells, in wild-type *C. elegans,* despite reduced U1 RNA levels, genes upregulated upon heat shock were preferentially enriched for long-intron-containing genes. This was visible in the increase in their average gene lengths (Fig 3i) and the cumulative distribution plots of gene lengths of differentially upregulated and downregulated genes (p.adj <0.05) (Suppl. Fig 8a), and metagene profiles of expression values of all expressed genes segregated by gene length (Suppl. Fig 8b, c; Suppl. Table 9). *prde-1* animals showed a marked deficiency in upregulating long genes, although they did not differ from wild-type in downregulation of long genes (Fig. 3i, Suppl. Fig 8a; also see Suppl. Fig 8b, c). This raised the intriguing possibility that the 22G-RNAs generated by wild-type animals were functionally substituting for U1 snRNAs during stress, ensuring productive Pol II elongation and expression of long-intron-containing genes. In support of this, ChIP–qPCR showed that elongating Pol II (S2P) levels were markedly and significantly decreased at *hsps (C12C8.1* and *F44E5.4/.5)* 3′ ends in *prde-1*, despite normal occupancy at the 5′-end, mirroring phenotypes caused by disrupting U1–nascent RNA interactions^51,52^ (Fig 3j; Suppl. Fig 8d).

Together, these findings indicate that the piRNA pathway delays splicing and promotes chromatin retention of mature mRNAs by associating with the transcription complex. In doing so, the antisense 22G-RNAs act in a manner strikingly similar to U1 telescripting: both systems protect splicing, preserve Pol II elongation, and the expression of long-intron containing genes, essential for development.

## The piRNA pathway preserves transcript fidelity and complements nonsense-mediated decay as a mechanism enforcing RNA quality control.

**Figure 4.**
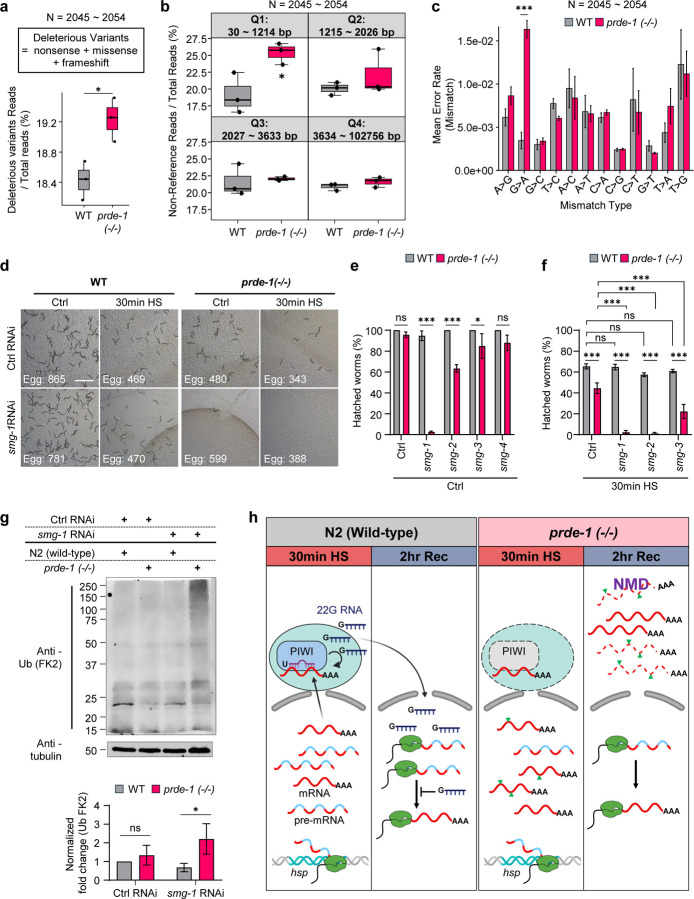
piRNA protects transcript fidelity and complements nonsense-mediated decay (NMD) to maintain protein quality control. **a.** Percentage of nonsense, missense, and frameshift variants containing mRNA molecules in wild type and *prde-1* mutants (variants identified by GATK and SnpEff). Y-axis: % variants. X-axis: Strain. Total variant containing sites=2045–2054. Approximately 2.7 × 10^7^ individual mRNAs analyzed. Boxplots: median value depicted. Error bars: standard error. Statistical analysis: two-tailed unpaired t-test.Figure 4. piRNA protects transcript fidelity and complements nonsense-mediated decay (NMD) to maintain protein quality control. **b.** Gene length stratification of mRNA variants in *prde-*1. Percentage of variants among total variant-containing mRNA molecules expressed from genes of different lengths in wild type and *prde-1* mutants. Q1-Q4: Genome divided into quantiles by gene length, as in Suppl. Fig 8b, c (gene lengths in bp). Y-axis: % variants. X-axis: Strain. Boxplots: median value depicted. Error bars: standard error. Statistical analysis: two-tailed unpaired t-test. **c.** Mean mismatch rates amongst variant-containing mRNA molecules. Y-axis: Mean error rate (number of mismatch reads/total reads with variants). X-axis: Mismatch. Total variant containing sites=2045–2054. Approximately 2.7 × 10^7^ individual mRNAs analyzed. Bars: mean value. Error bars: standard error. Statistical analysis: two-tailed unpaired t-test. **d-f.** Dependence on NMD. **(d)** Representative DIC micrographs of progeny from control and heat-shocked WT and *prde-1* animals on Ctrl (L4440) and *smg-1* RNAi. The number of eggs laid by the mothers in one representative experiment is shown. Note: WT progeny have grown into adults. Scale bar: 2mm. (**e, f**) Percent viable embryos from mothers subjected to RNAi-mediated downregulation of NMD components under **(e)** control, and **(f)** heat shock conditions. Y-axis: Percentage of viable embryos. X-axis: Strain and RNAi-treatment. Bars: mean value. Error bars: standard error. n = 3~5 biologically independent experiments. Statistical analysis: 2 way-ANOVA. **g.** Polyubiquitinated protein levels in WT and *prde-*1animals exposed to control (L4440) or *smg-1* RNAi. **Top:** Representative western blot (antibody:clone FK2). Tubulin: loading control. **Bottom:** Quantification of polyubiquitinated protein (normalized to wild-type Control RNAi-treated animals). Bars: mean value. Error bars: standard error. n = 5 biologically independent experiments. Statistical analysis: 2 way-ANOVA. **h.** Model for the mechanism through which piRNA/22G-RNA pathway regulates splicing.

The accelerated splicing observed in *prde-1* mutants, even under heat shock conditions when spliceosome function is challenged, and reduced RNA polymerase II occupancy at the 3′ ends of *hsp* genes (Fig 3j, Suppl. Fig 8d), suggested to us that in the absence of piRNAs, mRNA transcripts may accumulate errors due to aberrant splicing or premature termination. To directly test this hypothesis, we used Unique Molecular Identifiers (UMIs)^55–59^ to label individual mRNAs from heat-shocked wild-type and *prde-1* mutants and selected single mRNA molecules (free of PCR amplification artifacts) to analyze for the accumulation of transcript variants or ‘errors’ (Suppl. Online Material). To generate an ‘error rate’, we mapped the individual RNAs onto the *C.elegans* reference genome, excluded SNPs that could be attributed to potential genomic changes due to the *prde-1* mutation (see Methods for details), and identified high quality variant containing sites using the program Genome Analysis Toolkit (GATK), as regions where one or more mRNAs contained mismatches or misalignments when compared to the reference genome. To increase our confidence that any mismatches were ‘errors’, we further selected regions with a minimum coverage > 10 reads. We then determined the fraction of mRNAs in wild-type and *prde-1* animals that contained these mismatches (see Methods and Suppl. Table 10). Amongst the approximately 2.7 × 10^7^ individual mRNAs, we identified 2045–2054 variant containing sites, and of these, a larger fraction of mRNAs in *prde-1* mutants had one or more variants (Suppl. Fig 9a; p.adj = 0.133). This was particularly true when we analyzed nonsense, missense, and frameshift variants, called by the program SnpEff (Fig 4a; p.adj <0.05). Notably, the *prde-1* variant mRNAs were not uniformly distributed across the genome; instead, they were disproportionately enriched in mRNAs from smaller genes, which were also more highly expressed by *prde-1* (Fig 4b; also see Suppl. Fig 8c). Amongst the base changes G>A was the most highly represented (Fig 4c), consistent with the increased error rates associated with misincorporation of faster-incorporating bases by Pol II^60^. This suggested that the splicing delay imposed by the piRNA pathway protected transcript fidelity under stress, and in its absence, animals may be accumulating more ‘errors’ in their transcripts. However, this analysis did not exclude the possibility that these variants represented RNA modifications or increased RNA-editing in the *prde-1* animals.

Therefore, to independently evaluate whether *prde-1* animals accumulated more splicing errors, we examined whether they were more susceptible to the downregulation of the nonsense-mediated mRNA decay (NMD) pathway, a system that surveils and eliminates transcripts that are unproductively spliced^61–66^. Remarkably, downregulating *smg-1*, the NMD kinase, resulted in the complete loss of viability of *prde-1* embryos, not only upon heat shock but also under control conditions (Fig 4d–f). Downregulating *smg-2* (homolog of UPF1)^67^ and *smg-3* (homolog of UPF2)^67^ in parents also resulted in complete or partial loss of viability in their embryos, again even under control conditions (Fig 4e), or if parents were subjected to heat shock (Fig 4f). In contrast, wild-type embryos were not affected by *smg-1, smg-2, smg-3* RNAi, under either heat shock or control conditions (Fig 4d–f), consistent with previous studies showing that NMD is not required for *C. elegans* survival^61,67,68^. The involvement of NMD suggested that the mis-transcribed mRNA in *prde-1* was likely being eliminated during translation. In support of this, in the absence of *smg-1*, *prde-1* animals accumulated significantly higher amounts of polyubiquitinated proteins even under control conditions, in the absence of proteotoxic stress (Fig 4g; see methods for how viable *smg-1*-RNAi-treated *prde-1* were obtained). Taken together these studies suggest that piRNAs and NMD act in complementary modes of RNA quality control: piRNAs delay splicing to preserve accurate pre-mRNAs under stress, while NMD serves to eliminate erroneous transcripts when this buffering mechanism is lost (Fig 4h).

## Discussion.

In summary, using *hsp* mRNAs as the paradigmatic model for inducible gene expression, we uncovered a surprising role for the piRNA pathway in regulating splicing during the heat shock response, when spliceosome activity is compromised^36,37,53^. We found that upon heat shock, newly induced *hsp* mRNAs become targets for piRNA surveillance, rapidly becoming associated with PIWI (PRG-1) and producing piRNA-induced antisense 22G-RNAs. These 22G-RNAs interact with RNA polymerase II and nascent *hsp* pre-mRNAs to regulate splicing and delay transcript maturation and translation. Remarkably, this piRNA-dependent mechanism is necessary to safeguard mRNA fidelity. In the absence of piRNAs, splicing is accelerated, leading to transcript errors and increased dependence on NMD. Thus piRNA-regulation of splicing complements NMD as an RNA quality control mechanism.

Our studies also suggest that the function of the piRNA pathway extends beyond *hsp* genes to regulate alternative splicing, and the expression of long genes, many of which also template 22G-RNAs during stress. The 22G-RNAs that modulate *hsp* splicing are part of a larger population of piRNA- and *hsf-1-* dependent ‘unclassified” 22G-RNAs, whose levels increase during recovery from heat shock along with their complementary mRNA targets, suggesting that this pool of endo-siRNAs may mark post-transcriptionally spliced or processed genes, although this remains to be shown. Also, we are yet to identify the detailed mechanisms of splicing regulation. Still, the similarities to U1 RNA-mediated splicing and telescripting^50–54^ are striking: both systems use base-pairing of noncoding RNAs to stabilize nascent transcripts, bias splicing, stabilize pre-mRNAs, prevent the premature loss of elongating Pol II from the 3’ end of genes, and protect the expression of long-intron-containing genes. These parallels raise the intriguing possibility that, analogous to therapeutic antisense oligonucleotides explored for clinical applications, *C. elegans* may employ piRNA/22G RNAs as a natural small RNA–guided telescripting system to substitute for, or complement, U1 RNAs for RNA splicing. Post-transcriptional control of gene expression is emerging as a critical mechanism to regulate development, stress, and phenotypic plasticity^39,48^. Yet, how cells select transcripts for delayed splicing, how such delays are mechanistically implemented, and if and how splicing decisions are coordinated across the diverse tissues is poorly understood. The sequence complementarity of 22G-RNAs to expressed exonic regions of mRNAs, and their responsiveness to transcriptional and translational events within the cell, might allow them to act as flexible modulators of splicing decisions.

Several cell-autonomous activities of small RNAs, including piRNAs have been described, across species, and in *C. elegans*. These include post-transcriptional gene silencing through RNA cleavage, RNA decay, translation repression, transcriptional gene silencing through inhibition of RNA polymerase II (Pol II)-mediated transcript elongation and cDNA or histone methylation^5–7,69,70^. Only very few previous studies have linked the piRNA pathway to splicing regulation^71,72^. Yet, these studies suggest that the piRNA-splicing-regulation, and its functions may be conserved across species. Thus, in *Drosophila*^71^ nuclear Piwi complexes inhibit splicing of the *gypsy* retrotransposon through chromatin-based, co-transcriptional silencing, whereas in locusts^72^ piRNAs facilitate splicing of specific pre-mRNAs during oocyte maturation, suggesting both inhibitory and stimulating role for piRNAs in splicing control. Intriguingly, in *C. elegans*, the presence of introns protects transcripts from default Argonaute-mediated silencing in the germline, and the WAGO Argonaute, HRDE-1 have been shown to interact with the conserved RNA helicase Aquarius/EMB-4^73–75^, further implicating piRNA function in RNA splicing regulation. In our studies, the 22G-RNAs appear capable of crossing cell boundaries, suggesting a potential role in coordinating splicing across tissues. Also, remarkably, the 22G-RNAs antisense to *hsp* mRNAs were enriched in sperm, an observation that parallels the presence of piRNAs in mature mammalian sperm^76^, where they are thought to influence sperm quality, motility, and epigenetic inheritance.

In *C. elegans*, loss of the piRNA pathway leads to a sporadic, and temperature-dependent increase in infertility over time, a defect that can be mitigated by slowing growth or metabolism^9,77^. In addition, NMD, which mainly silences pseudogenes, is crucial for longevity^78,79^. Thus, our findings suggest that, similar to aging-dependent neurodegenerative disorders, the increased infertility upon piRNA loss may be due to the progressive accumulation of RNA errors arising from the loss of piRNA-control over splicing rates, and increased misfolded proteins due to an overwhelmed NMD system. Taken together with the known roles of piRNAs in transposon and pseudogene regulation of germline mRNAs, and the growing evidence for links between splicing, genome stability, and transposon activation^80^, our findings suggest that piRNAs pathway may serve as a conserved node to integrate genome surveillance with RNA and protein quality control.

## C. ELEGANS STRAINS AND GROWTH CONDITIONS

*C. elegans* used in all experiments are listed in Table 1. Strains were obtained from the *Caenorhabditis Genetics Center* (CGC, Twin Cities, MN), generated in the Prahlad laboratory, or generated by SunyBiotech (Suzhou, Jiangsu, China). All crossed worms were verified by genotyping. The primers used in genotyping are listed in Table 2.

All strains were maintained at 20°C on nematode growth media (NGM) plates. NGM plates were prepared by pouring 8.9 ml autoclaved liquid NGM per 60 mm plate. NGM was allowed to solidify at room temperature (RT) for 2 days, seeded with 300 μl OP50 *E. coli* (O.D = 1.8~2.0), and dried at RT for no more than 2 days. Population densities were matched based on growth rate of the strains. To maintain healthy continuously growth populations, 10~15 animals at larvae stage 4 (L4) were passed onto a new plate every 4 days. For experiments, day-one adult animals were used. These were generated either by picking L4-stage worms and harvesting them 24~24 hrs later, or by bleach-hatching and collecting day-one adults worms 82~84 hrs later, depending on experimental requirements.

## HEAT SHOCK

Worms were exposed to acute heat stress as previously described in Das et al., elife 2020^1^. Briefly, NGM plates containing day-one adults were sealed with parafilm and immersed in a water bath pre-warmed to 34°C for different duration as mentioned. For recovery experiments, NGM plates were incubated at 20°C incubator immediately after heat shock and harvested after specific times mentioned in the experiments.

## RNA SEQUENCING

### RNA Library Preparation

For RNA-sequencing, three independent biological replicates of each strain and each treatment were harvested and processed as described. For each sample/treatment, approximately 200 L4-stage worms were transferred to OP50 plates. After 26~27hrs, worms from two plates per condition [non-heat shock (NHS), 5min HS, 30min HS, and 30min HS + 2hr Rec] were collected in nuclease-free water. Total RNA was extracted using the Mirvana miRNA Isolation Kit (Invitrogen; Cat #AM1560). Genomic DNA in the sample was removed using Turbo DNA-free Kit (Invitrogen; Cat #AM1907). RNA was separated by size into mRNA (>200 nt) and small RNA (<200 nt). Small RNA samples were treated with 10U RppH (NEB; Cat #M0356S) at 37°C for 30 min, then purified using the Zymo RNA Clean & Concentrator kit (Zymo Research; Cat #R1015). RNA concentrations were measured with a Qubit 4 fluorometer (Thermo Scientific). mRNA libraries were prepared using the KAPA mRNA Hyperprep kit (Roche; Cat #08098123702); small RNA libraries were prepared using the Illumina TruSeq Small RNA Library Preparation kit (Illumina; Cat #RS-200), following manufacturer’s protocol.

### Small RNA analysis

Small RNA data was analyzed using the tinyRNA^2^ (v1.50) pipeline with its default configuration. The process included adapter trimming and quality filtering with fastp^3^, followed by genome alignment to the *C. elegans* genome (release WS279) using Bowtie^4^. An additional setting, counter_decollapse: True, was specified to generate decollapsed SAM files. The module tiny-count was used to count the different types of small RNA according to the selection rules specified in the following table.

**Table:** 

Select for...	with value...	Classify as...	Hierarchy	Overlap	Strand	5’End Nucleotide	Length
Class	risiRNA	rRNA	1	Partial	both	all	all
Class	miRNA	miRNA	2	5’Anchored, 0, 4	sense	all	all
Class	piRNA	piRNA	2	5’Anchored	sense	all	18–21
Class	CSR	CSR Class 22G	3	Partial	antisense	G	21–23
Class	WAGO	WAGO Class 22G	3	Partial	antisense	G	21–23
Class	ALG	ALG Class 26G	3	Partial	antisense	G	26
Class	ERGO	ERGO Class 26G	3	Partial	antisense	G	26
Class	ALG	ALG target 22G	3	Partial	antisense	G	21–23
Class	ERGO	ERGO target 22G	3	Partial	antisense	G	21–23
Class	unk	unclassifed 22G	3	Partial	antisense	G	21–23
Class	unk	unclassifed siRNA	4	Partial	both	all	21–24

The read counts obtained from tiny-count were used to generate genome-wide coverage profiles for the 22G-RNAs and piRNA. The coverage was normalized as Read per million (RPM) and output as BigWig (bw) files using rtracklayer^5^. Differential Expression was computed by using a modified version of tyny.deseq.r, the R module was adjusted to remove the ribosomal RNA (rRNA) from the analysis, and to calculate the gene expression in RPM in addition to DESeq normalized counts. Changes in expression were presented as log2 fold-change, and genes with an adjusted p-value of <0.05 (after correction with Benjamini & Hochberg)^6^, were considered significant.

### mRNA seq analysis

RNA-seq samples were analyzed and processed using the nf-core/rnaseq (v3.12.0) pipeline, which was executed with Nextflow^7^ (v22.10.6). Sequence quality was assessed with FastQC ^8^(v0.11.9), and low-quality reads and adapters were removed using Trimmomatic^9^ (v0.67). The processed reads were then aligned to the *C. elegans* genome (WBcel235, Ensembl release 111) with STAR^10^ (v2.7.9a) using default settings. Alignments were quantified against the WBcel235 annotation with Salmon (v1.9.0). Alignment quality control was performed with Qualimap^11^ (v2.2.2) and RSeQC^12^ (v3.0.1).

### Differential Expression

Differential expression analysis was performed between RNA-seq samples using **DESeq2** with the **Wald test**. Expression changes were presented as log2 fold-change. Genes with an adjusted p-value <0.05, following correction with the **Benjamini & Hochberg**^6^ method, were considered significantly differentially expressed.

### Analysis of Alternative Splicing Events

To identify differential alternative splicing events, RNA-seq data were re-processed using the nf-core/rnasplice (v1.0.4) workflow. This pipeline was executed with Nextflow (v23.10.0) and relied on reproducible software environments from Bioconda and Biocontainers.

Within the rnasplice pipeline two modules were used to detect differential splicing and calculate the Percent Spliced-In (PSI) for each event, SUPPA2^13^ (v2.3) and rMATS (v4.1.2)., Splicing events were considered significant if the absolute difference in PSI (dPSI) between the 30 minutes heat shock and control conditions had a p-value <0.1.


$$\text{Formula}:\Psi =\sum \left(TPMinclusion\right)+\sum \left(TPMexclusion\right){\sum \left(TPMinclusion\right)}^{13}$$

Pearson’s Chi-squared test with Yates’ correction was used to determine if the number of up- or down-regulated splicing events during heat shock differed between the N2 and PRDE1 strains (p-value <0.05).

Differential Transcript Usage (DTU) analysis was performed using SUPPA2. First, usage of transcripts (isoforms) were tested for significant differences between conditions. Isoforms with a p <0.1 were considered significant. For analysis and visualization, the average Percent Spliced In (PSI or Ψ) value for each isoform was calculated across the biological replicates within each condition. The resulting list of PSI per isoforms was then filtered to retain only those occurring in genes previously identified as differentially expressed (DEGs). Student t-test was used to compare the means between the conditions at significance level p <0.05.


Formula:PSI_i=TPM_i/Σ_j=1^nTPM_j13

### Gene Ontology

Gene Ontology analysis was performed by using Over Representation Analysis (ORA), with the R package ClusterProfiler^14,15^. GO annotations for *C. elegans* were obtained from R package org.Ce.eg.db: Genome wide annotation for Worm^16^ (version 3.18.0). Ontology terms enriched with an adjusted p-value <0.05 or <0.1 (Benjamini & Hochberg)^6^ were considered significant. Fold-Enrichment was calculated as the ratio of the frequency of input genes annotated in a GO term to the frequency of all genes annotated to that term^14^.

### Data Visualization

The metaprofiles were generated using Deeptools^17^ (v3.56) by normalizing the coverage by RPM per bp) and then modified to plot them using R.

Venn diagrams were generated using the Eulerr^18^ package (v.7.0.2) in R^18^. Additional plots were generated using ggplot2 (v.3.5.1)^19^ .

## RNA FISH

### mRNA FISH

RNA FISH was performed as previously described^20^. Probes were designed against mRNA according to the Biosearch Technologies website, and consisted of 25–35 11bp sequences that complementary to the *hsp-70s (C12C8.1* and *F44E5.4/.5)* mRNA. For mRNA FISH, about 20 day-one adult worms were harvested in 1x PBS and fixed in 4% paraformaldehyde (Electron Microscopy Science; Cat #50-980-487) for 45 mins. After two washes with 1x PBS, worms were permeabilized in 70% ethanol at 4°C for 24~26 hrs. Worms were washed with Stellaris Buffer A (Biosearch Technologies, Cat # SMF-WA1–60) and incubated at 37°C for 16 hrs in hybridization buffer (Cat #SMF-HB1–10) containing 0.2 mM FISH probes targeting *hsp-70s (C12C8.1* and *F44E5.4/.5)* mRNA. After washing three times with pre-warmed Wash Buffer A and one time with Wash Buffer B (Biosearch Technologies, Cat # SMF-WB1–20), worms were mounted in Vectashield with DAPI (Vector Laboratories; Cat #H-1200–10). Imaging was performed using a Leica TCS SPE Confocal Microscope with 20x dry and 63x oil objectives, and LAS AF software.

### 22G RNA FISH

For 22G-RNA FISH, animals were processed as described above for mRNA FISH. Fluorescence conjugated probes targeting 22G-RNAs were purchased from IDT, and were complementary to one of the most abundant 22G-RNAs identified by sRNA-seq (hence they were in the sense direction as mRNA, and could not hybridize to mRNA). 0.2 μM FISH probe targeting *F44E5.4/.5* 22G RNA was used per slide. Sequence of the probe is in Table 3.

## AUXIN-INDUCIBLE DEGRADATION (AID) TREATMENT

Auxin plates were prepared at a final concentration of 4 mM by adding indole-3-acetic acid (Alfa Aesar; Cat #A10556), dissolved in ethanol at 400 mM, to autoclaved NGM that had cooled to approximately 55°C prior to pouring. NGM plates with ethanol served as controls. Plates were dried at RT for 2 days, then for 2 more days after seeding with 300 μl OP50 (O.D = 1.8~2.0). About 20 day-one adults were transferred to OP50-seeded auxin or ethanol plates and incubated at 20°C for 4 hrs. Worms were then either not heat shocked (NHS) or subjected to heat shock (HS) for 30mins at 34⁰C and processed for FISH as above.

## IMMUNOSTAINING

Immunostaining of dissected gonad was performed as previously described^20^ . 10~15 day-one adults were picked into 15 μl 1x PBS on a cover glass (Leica Biosystem; Cat #3800105) and dissected with a surgical blade #11. Dissected worms were fixed in 4% paraformaldehyde for 6 mins. A charged slide (Epredia; Cat #4951PLUS-001) was placed over the cover glass, then quickly transferred onto a metal block that had been cooled with dry ice for over 10mins. After quickly removing the cover glass, samples were post-fixed in 100% methanol pre-chilled at −20°C for 2mins, washed in 1x PBST (1x PBS with 0.1% Tween-20), and then blocked in 1x PBST with 0.5% BSA for 1 hr. Samples were incubated at 4°C overnight with Mouse anti-FLAG M2 (Sigma-Aldrich, Cat #F3165–1MG), diluted 1:2,000 in 1x PBST. Following washing, samples were incubated at RT for 2 hrs with Donkey anti-Mouse Alexa Fluor 488 (Invitrogen; Cat #A32766), diluted 1:500 in 1x PBST. After washing, samples were mounted in the Vectashield with DAPI and imaged.

## RNA ISOLATION AND QUATIFICATION OF RNA LEVELS BY RT-qPCR

RNA was extracted from 30~40 day-one adults exposed to indicated heat shock durations and recovery times. Animals were collected in 50 μl Trizol and immediately snap-frozen in liquid nitrogen. Samples were thawed on ice, mixed with 200 μl Trizol, and lysed using Precellys 24 homogenizer. RNA was isolated from lysates with chloroform and precipitated with isopropanol and glycogen. After washing with 70% EtOH, RNA pellet was dissolved in nuclease-free water and treated with TURBO DNase. cDNA was synthesized using iScript cDNA synthesis kit (Bio-Rad; Cat #1708891). qPCR was performed using PowerUp SYBR Green Master Mix (Applied Biosystems; Cat #A25742) on a QuantStudio 3 Real-time PCR System (10 μl reaction, 96-well plate).

The amounts of RNA were quantified using one of the following methods:

### mRNA RT-pPCR

- Relative quantification using the ΔΔCt method: mRNA (exon-exon junction primers), and pre-mRNA (intron-exon primers) levels were computed relative to *pmp-3* (previously verified by use to remain steady during heat shock)^1^, which served as an internal control. Primer locations are depicted in the Method Fig 1 below and primer sequences are in Table 2.
- Absolute quantification using standard curves: Absolute amounts of pre-mRNA and mRNA were estimated through the generation of standard curves using serial dilutions of defined concentrations of *hsp-70 (C12C8.1)* and *F44E5.4/.5* mRNA, pre-mRNA, as well as *pmp-3* mRNA. The mRNA and pre-mRNA used to generate each standard curve, was obtained by PCR amplification from cDNA or genomic DNA extracted from whole worms, PCR amplified using primers external to the primer target sites used in the experiments, gel purification, and quantification using a Qubit 4 fluorometer. qPCR was conducted using 10-fold serial dilutions of a known concentration of purified PCR products. The standard curves were created from the CT values obtained from the dilutions (see Method Fig 1).
  The amounts of mRNA and pre-mRNA were extrapolated derived from these curves using the formula.
$$\text{Formula}:Absolute\;amount\;of\;RNA=Initial\;Con.\times 10^{\left(\frac{CT\;Value-constant}{slope}\right)}$$
  To account for differences in lysis efficiency, variations in harvesting of animals, and other factors, a similar curve was calculated for *pmp-3* and the final values were normalized to *pmp-*3 values known to remain stable through heat shock^1^.
  The primers used for qPCR analysis are designed using Primer 3 software and generated by Integrated DNA Technologies (See Table 2).

### SMALL RNA (sRNA) RT-qPCR

Small RNA qPCR was performed using stem-loop RT-qPCR by modifying published protocols^21^ Total RNA extracted from animals, or immunoprecipitated after 4sU treatment and RNAPII immunoprecipitation (see below), incubated with 50 nM 22G RNA-specific stem loop primer at 70°C for 5mins, cooled on ice for ≥5 mins. cDNA was synthesized using 100U Superscript II Reverse Transcriptase (Invitrogen; Cat #18064014) in 1x reaction buffer, 1U SUPERase-In RNase inhibitor, and 0.5 mM dNTPs. The RT-qPCR mix included 2 μl cDNA, 0.25 μM 22G RNA-specific forward primer, 0.25 μM universal reverse primer,1x PowerUp SYBR Green Master Mix. Relative fold change of 22G-RNA was quantified relative to immunoprecipitated *pmp-3* using the ΔΔCt method. Primer specificity was tested using a synthetic sRNA (21ur-2348). Specificity of the reaction was verified by running out the reaction on agarose gels. Representative gel and PCR design are shown in Suppl. Fig 7c. The sequence of primers is listed in Table 3.

### ChIP-qPCR

Chromatin immunoprecipitation (ChIP) was performed as previously described^20^ . Following exposure to one of three conditions (NHS, 30min HS, or 30min HS + 2hr Rec), approximately 700 day-one adults, synchronized by bleach hatching, were harvested in 1x PBS and crosslinked with 2% formaldehyde at RT for 10 mins. Crosslinking was quenched with 250 mM Tris (pH 7.4) for 10 mins. Samples were washed three times in ice-cold 1× PBS containing protease inhibitor cocktail (PIC; Thermo Scientific; Cat #78429) and then snap-frozen in liquid nitrogen. Pellets were resuspended in 200 μl FA buffer (50 mM HEPES/KOH (pH 7.4), 150 mM NaCl, 50 mM EDTA, 1% Triton X-100, 0.1% sodium deoxycholate, 0.5% sarkosyl) freshly supplemented with 1 mM DTT and 1x PIC. Worms were lysed using a Precellys 24 homogenizer and sonicated (Bioruptor Pico; 8~10 cycles of 30sec on/off). Debris was removed by centrifugation (13,500 rpm at 4°C for 5 mins). Protein A/G Magnetic Beads (20 μl per sample; Thermo Scientific, Cat #88803) were washed two times with FA buffer. To eliminate non-specific binding, 5 μl of beads were then added to the lysates and incubated at 4°C for 1 hr. 10% of the pre-cleared lysate was kept as input, and the rest was incubated overnight at 4°C with anti-total RNAPII (Diagenode; Cat #C1520004) or RNAPII s2P (Diagenode; Cat #15200005), followed by 3 hrs incubation with 15 μl beads. Beads were washed sequentially with low salt, high salt, LiCl, and TE buffers, then eluted in 150 μl elution buffer (10 mM EDTA, 1% SDS, 0.1 M sodium bicarbonate). Elutes and volume-matched inputs were de-crosslinked overnight with 0.8U proteinase K (NEB; Cat #P8107S) and 160 mM NaCl. DNA was purified using phenol/chloroform/isoamyl alcohol. qPCR was performed as above. All experiments were expressed as % input value. The sequence of the primers used for ChIP experiments are listed in Table 2 and depicted in Fig 3j; Suppl. Fig 5e, f, and 8d.

## RNA IMMUNOPRECIPITATION (RIP) FOLLOWED BY RT-qPCR

RNA immunoprecipitation (RIP) was performed with ~700 day-one adults per condition, expressing FLAG::FRG-1 (endogenous PRG-1 tagged with FLAG epitope using CRISPR/Cas9) synchronized by bleach hatching. To account for the non-specific interactions of the abundant *hsp* mRNA generated by heat shock, we used wild-type (N2) animals not expressing FLAG as a negative control, and compared specific immunoprecipitation (IP) of PRG-1 IP to this non-specific IP. RIP was conducted in the absence of cross-linking. Worms were harvested in nuclease-free water, pelleted, and snap-frozen in liquid nitrogen. Worms were resuspended in FA buffer with 1 mM DTT, 1x PIC, and 0.01U/μl SUPERase-In RNase inhibitor (Invitrogen; Cat #AM2696), then lysed and sonicated (2 cycles of 30 sec on/off). 10% of the pre-cleared lysate was kept as input, and the rest was incubated with anti-FLAG M2 (Sigma-Aldrich, Cat #F3165–1MG) at 4°C for 2~3 hrs, followed by beads for 1hr. After washing, beads were eluted with elution buffer containing 0.01U/μl SUPERase-In RNase inhibitor. Elutes and volume-matched inputs were de-crosslinked by incubating them with 0.8U proteinase K (NEB; Cat #P8107S) at 45°C for 1hr. RNA was isolated using Trizol and qRT-PCR was performed as above. Because we were had no reason to expect that *pmp-3* would also bind to PRG-1, and be immunoprecipitated, to control for variation in harvesting, lysis, loading etc., we normalized *hsps* in IP sample to *pmp-3* in the input sample using the ΔΔCt method. *bath-45*, known to interact with PRG-1 was used as a positive control for the efficacy of RIP. The sequence of the primers used for RIP experiments are listed in Table 2.

### 4sU RNAPII IP qPCR

All procedures for 4sU RNAPII immunoprecipitation (IP) are outlined in Fig 3a. Specifically, 4sU was delivered as a liquid suspension mixed with bacterial food. The bacterial food, OP50, was heat-killed to minimize its metabolism of 4-Thiouridine (4sU). To most optimally deliver 4sU within a short time, about 700 day-one adult worms were rapidly collected in nuclease-free water and then resuspended in S-basal containing 10mg/ml heat-killed OP50 and 4mM 4sU (Sigma Aldrich; Cat #T4509). The suspension was seeded onto empty NGM plates to prevent hypoxia (a critical step since *C.elegans* suspended in liquid media have a markedly different heat shock response). The worms, bacteria and 4sU were allowed to dry at RT for 1hr, and subjected to one of three conditions [not heat shocked (NHS), heat shocked at 34⁰C for 30 mins (30min HS), or heat shocked at 34⁰C for 30 mins and allowed to recover for 2 hrs at 20⁰C (30min HS + 2hr Rec)], and then crosslinked by exposure to UV (365 nm) for 30 sec. Samples were harvested in nuclease free water and snap-frozen in liquid nitrogen. Worm lysis, immunoprecipitation, RNA isolation, and qRT-PCR followed the RIP-qRT-PCR protocol, using anti-total RNAPII (Diagenode; Cat #C1520004) instead of anti-FLAG. To quantify mRNA and pre-mRNA, absolute amounts of RNA were estimated from standard curves and normalized by immunoprecipitated *pmp-3*.

## EU LABELLING OF NASCENT RNA

To examine the expression patterns of nascent *hsp* genes we used a pulse-chase approach using the Click-iT Nascent RNA Capture Kit (Invitrogen; Cat #C10365) according to the manufacturer’s instructions, to label RNA. In brief, 50 day-one adult worms were placed on an OP50 bacterial lawn containing 4 mM EU and incubated at 20°C for 10 mins to preload them with EU. Subsequently, the worms were incubated at 20°C (serving as controls) or underwent heat shock for 30 mins at 34°C. Control and heat shocked animals were harvested in 50 µl Trizol and immediately snap-frozen in liquid nitrogen. To assess maturation of pre-mRNA to mRNA, animals were allowed to recover in the absence of EU. For this, worms were transferred, immediately after heat shock, from EU containing OP50 plates onto new NGM plates seeded with regular OP50. The plates were then incubated at 20°C for 2, 4, or 6 hours, and subsequently harvested in Trizol. RNA isolation was performed as described in the RT-qPCR protocol. EU-labeled RNA was biotinylated with 0.4 mM biotin azide and purified by reprecipitating. The biotinylated RNA was captured using 12 µl of streptavidin beads. cDNA synthesis was carried out directly on the beads containing nascent RNA using the iScript cDNA synthesis kit. qPCR was processed as described in the RT-qPCR protocol. To quantify mRNA and pre-mRNA, absolute amounts of RNA were estimated from standard curves. To assess maturation from pre-mRNA to mRNA, mRNA expression level at each time point was divided by pre-mRNA expression at 30min HS. In both analysis, mRNA and pre-mRNA expression amounts were quantified relative to EU labeled *pmp-3* amounts.

## SUBCELLULAR FRACTIONATION

Subcellular fractionation was performed with ~700 day-one adults per condition synchronized through bleach hatching method. Worms were harvested in 1.5 ml tube with nuclease free water, pelleted and snap-frozen in liquid nitrogen. Frozen pellet was washed with 500 μl hypotonic buffer (15 mM HEPES/KOH (pH7.5), 10 mM KCl, 5 mM MgCl2, 0.1 mM EDTA, 350 mM Sucrose). Worms were resuspended in 100 μl hypotonic buffer supplemented with 1 mM DTT, 1x PIC and 0.01U/μl SUPERase-In RNase inhibitor and homogenized by pestle (SP Bel-Art ; Cat #F19923–0001). To remove worms’ bodies and debris, homogenates were centrifuged at 500 xg at 4°C for 5 mins, and supernatant was transferred to a new 1.5 ml tube. The supernatant was then centrifuged at 4,000 xg at 4°C for 5 mins to collect nucleic (pellet) fraction.

The nuclei pellet was washed three times with 200 μl hypotonic buffer. After last washing, the pellet was resuspended in 120 μl nucleus lysis buffer (50 mM Tris (pH7.5), 140 mM NaCl, 1.5 mM MgCl2, 0.5% (w/v) NP-40) supplemented with 1 mM DTT, 1x PIC and 0.01U/μl SUPERase-In RNase inhibitor, and incubated in ice for 20 mins. 20 μl of sample was aliquoted to a 1.5 ml tube labelled as “input”, and the remaining samples were centrifuged at 20,000 xg at 4°C for 10 mins. The supernatant was transferred to a 1.5 ml tube labelled as “nucleoplasm”. The pellet was resuspended in 100 μl high salt solution (10 mM Tris (pH7.5), 700 mM NaCl) supplemented with 1 mM DTT, 1x PIC and 0.01U/μl SUPERase-In RNase inhibitor, and incubated in ice for 20 mins, and centrifuged at 20,000 xg at 4°C for 5 mins. The supernatant was transferred to a 1.5 ml tube labelled as “Chromatin”. The RNA in each sample was isolated using Trizol, and *hsp* gene expression level was measured as described in the RT-qPCR protocol. The results of experiments were presented as percentage input values.

This method of extraction did result in the loss of some nuclei. Nevertheless, the nuclei obtained were ‘clean’ and not contaminated with cytoplasmic fractions, confirmed by conducting a Western blot and probing for tubulin.

## RNA INTERFERENCE (RNAi)

RNAi experiments were conducted using the standard feeding RNAi methods. Bacterial clones expressing the control (empty vector pL4440) construct and the dsRNA targeting different *C. elegans* genes were obtained from the Ahringer RNAi library^22^ now available through Source Bioscience. All RNAi clones used in experiments were sequenced for verification before use. For RNAi experiments, RNAi bacteria with empty (pL4440 vector as control) or the dsRNA-expressing plasmid were grown overnight in LB liquid culture containing ampicillin (100 μg/ml) and then induced with IPTG (1 mM) for 2 hrs before seeding the bacteria on NGM plates supplemented with ampicillin (100 μg/ml) and IPTG (1 mM). Bacterial lawns were allowed to grow 48hr before the start of the experiment. RNAi seeded plates were used for embryo hatch rate experiments or western blot experiments for ubiquitinylated proteins.

## EMBRYO HATCH RATE

Embryo hatch rate was assessed using 2~5 day-one adult worms, which laid eggs for different durations following NHS or 30min HS, as described previously^20^ . After removing mothers, embryos were counted and incubated at 20°C. The number of live progenies were scored after 72 hrs. For RNAi experiment, mothers were prepared by transferring them to RNAi plates at L4-stage and allowed to lay eggs for 12 hrs after treatment.

## WESTERN BLOT

Worm lysates were prepared by boiling samples in Laemmli sample buffer (Bio-Rad; Cat #1610737) with β-mercaptoethanol. For ubiquitinylated protein analysis, about 150 worms were grown on RNAi plates by transferring them 24 hours after bleach hatching from OP50 plate. This was done to bypass embryonic lethality of *smg-1* RNAi. The worms were collected, snap frozen, and lysed by boiling and grinding using a pestle. Lysates were separated on SDS-PAGE (HSP-70: 10%, Ubiquitinylated protein: 12%) and transferred onto nitrocellulose membrane (Bio-Rad; Cat #1620115). Membranes were blocked at RT for 1 hr in Odyssey Blocking Buffer (LI-COR; Cat #927–50000), incubated overnight at 4°C with primary antibodies, and then with IRdye goat anti-mouse IgG 800CW (1:10,000; LI-COR; Cat #926–32210) at RT for 2 hrs. Imaging was performed using the LI-COR Odyssey Infrared System and analyzed with Image Studio software.

To detect HSP-70 levels, mouse anti-FLAG M2 (1:1000; Sigma-Aldrich; Cat #F3165–1MG) was used to detect HSP-70::FLAG in strains where endogenous *hsp-70* (C12C8.1) was tagged with CRISPR/Cas9. Mouse anti-Ubiquitinylated proteins, clone FK2 (1:1000; Sigma-Aldrich; Cat #04–263), was used to detect ubiquitinylated proteins. For tubulin detection as a loading control, mouse anti-tubulin, clone AA4.3 (1:1000; DSHB; Cat #AB_579793) was used.

## SMALL RNA INJECTION AND RECOVERY ASSAY

Small RNA was prepared from total RNA extracted from

- Wild-type animals worms subjected to 30 min HS
- *prde-1* animals subjected to 30 min HS

Small RNAs were fractionated using the Mirvana miRNA Isolation Kit. The small RNA was then injected at a concentration of 50 ng/μl. Following injection, worms were recovered at 20°C for 2 hrs and subsequently maintained under non heat shock conditions (NHS) or subjected to 30 mins heat shock at 34°C. Worms were collected in 1x PBS after 6 hrs incubation at 20°C and used for *F44E5.4/.5* mRNA FISH experiment. For RT-qPCR analysis, worms were collected immediately in Trizol, and their RNA harvested as described above.

## ANALYSIS OF mRNA VARIANTS

To assess whether the loss of piRNAs led to increased transcript errors, we devised a method to identify sequences of individual mRNA molecules prior to the possible incorporation of errors due to PCR amplification. RNA extracted from 3 biological repeats of: wild type *C. elegans* at 30 minutes at 34°C, and *prde-1* at 30 minutes at 34°C were used. mRNAs were tagged with UMIs using the **SMART-Seq**^**®**^ **Total RNA Pico Input with UMIs (ZapR**^**™**^ **Mammalian)**. The UMIs were attached to mRNAs during the first strand-cDNA synthesis, prior to PCR amplification. Subsequently, mRNAs were PCR amplified with 3 cycles of amplification for the initial library PCR and 12 cycles of amplification for the enrichment PCR. Sequencing was performed on the Novaseq 6000; the number paired-end reads sequenced were in the range of 50 million-80million, with a read size of 100 bp.

Next, data was pre-processed using a modified version of the RNA with UMIs v1.0.16 pipeline^23^ and a modified version of the GATK Best Practice: RNA-seq Variant Calling Workflow using the tools from gatk ( v4.6.1.0) and Picard (v3.3.0).

The complete list of relevant tools, and the custom script used are available on github (https://github.com/Prahlad-Lab/umi_celegans_analysis ) DOI 10.5281/zenodo.17430104.

The fastq.gz files required to run this analysis pipeline will be made available through the European Nucleotide Archive (ENA) at EMBL-EBI under accession number PRJEB101605 (https://www.ebi.ac.uk/ena/browser/view/PRJEB101605).

FastQC ^8^ was used to check the quality of the FASTQ paired-end reads. The FASTQ files were converted to unmapped bam (uBAM) using gatk FastqToSam tool. Unique molecular identifiers (UMI) were extracted using fgbio ExtractUmifromBam. The UMI were extracted using the parameters indicated in the SMART-Seq^®^ Total RNA Pico Input with UMIs (ZapR^™^ Mammalian) kit, the SMART UMI Adapter from read2 was removed by trimming reads a total of 12 nucleotides: “8 nt UMIs + 3 nt UMI linker + 3 nt”. The quality of the uBAM file was measured again with FastQC^8^.

Reads were aligned to genome with STAR^24^ (v 2.7.11b) using the *Caenorhabditis elegans* genomic reference and annotation (ENSEMBL WBcel235 version 114)^25^. Per-Sample two pass-mapping was enabled using the option -twopassMode Basic. The main output was an unsorted BAM that included unmapped reads, --outSAMtype was set to “BAM Unsorted” and -- outSAMunmapped was set to “Within”. A transcriptome-aligned BAM was also output with -- quantMode = Transcriptomes SAM. Additional alignment parameters were set to match Encode bulk-rna seq standards --outFilterType = BySJout, --outFilterMultimapNmax = 20, -- outFilterMismatchNmax = 999, --alignIntronMin = 20, --alignIntronMax = 1000000, -- alignMatesGapMax = 1000000, --alignSJoverhangMin = 8, and --alignSJDBoverhangMin = 1. The number of allowed mismatches were set following the parameter detailed in the BROAD pipeline outFilterMatchNminOverLread = 0.33 --outFilterMismatchNoverLmax = 0.1.

### UMI Grouping and Filter

Following alignment, BAM files were sorted by coordinating with Picard SortSam tool. UMI-tools^26^ was used to create subgroups based on UMI and sequencing errors within UMIs themselves were corrected. The function umi_tools group was used to output the BAM files and the following options were set : to specify the tag where the UMIs were stored, --extract-umi-method was set to “tag” and --umi-tag was set to “RX” and to output text files with stats about the groups, --group-out = .grouped.tsv The stats text file was used to obtain Family Size of the UMIs and their unique IDs. Around 50% of the reads in all the replicates were Family size =1, as shown in the plot.


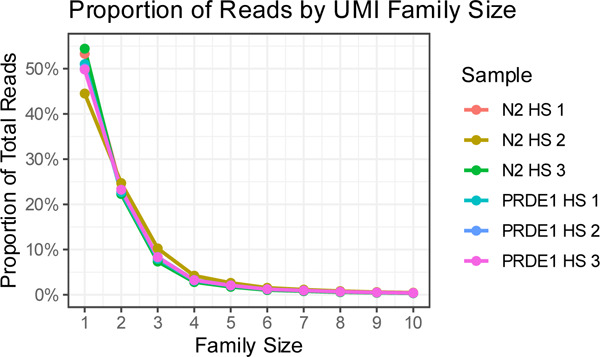


Ideally, we would have preferred to generate a consensus from the UMIs families which were in groups >1 (i.e. amplified by PCR), in order to extract the sequence prior to the introduction of PCR errors. However, since 50% (2.7–3.4 × 10^7^ individual reads) of the UMI-tagged mRNAs were singletons, i.e. corresponded to the original mRNA sequence and were not PCR amplified, we used these UMIs instead for our analysis (Table 4). Samtools was used to filter and sort the BAM files, using the unique IDs, to keep only the reads a family size of one (Singletons BAM).

Following the GATK best practices for RNA-seq Variants, the singletons BAM files were processed with gatk SplitNCigarReads to split the reads with “N” in their cigar. To filter out DNA variants, a recalibration model was built with gatk BaseRecalibrator, using Variant Calls from the Caenorhabditis Natural Diversity Resource^27^ (CaeNDR) for Hawaiian Strain CB4856 (CB4856.hard-filter.vcf.gz) to identify the --known-sites. The resulting model was applied to the singletons BAM files with gatk ApplyBQSR to obtain BSQR BAM.

### Pileup Generation and analysis

A pileup of the BSQR BAM was generated with bcftools mpileup using the parameter -d 1000000 to get keep the reads at all loci. **To increase confidence that we were detecting stochastic mRNA variants, we:**

- analyzed only regions that had generated over 10 reads (i.e. at least 10 mRNAs corresponded to the region to identify the ones which were different). The pileup file was analyzed with a custom python script to filter the regions with total depth <=10 and count the proportion of alternative reads and reference reads.
- Excluded regions where “Alternative Read/Total Reads =1.0”.
- A custom R script was used to generate tables with the statistics of the Total Number of “mistakes” and the Distribution of the Alternative Reads/Reference reads.

### Variant Call and Analysis

HaplotypeCaller was used with the BSQR BAM to call variants and output VCF file. SnpEff ^28^ was used to annotate the vcf with the SnpEff wbcel235 internal annotation. A custom R script was used to read the VCF and filter the variants with total dept < =10, and SnpEFF Functional Impact type of “Modifier”. Variant Sites with a Ratio of Alternative Read/Total Reads <1.0, were excluded to remove potential genomic Variants. The following were calculated:

- Transcription error rate: Ratio of Alternative Read/Total Reads
- Deleterious Variant Rates: Ratio Deleterious Variants/Total Reads
- Mismatch Error rate: change in nucleotides type.
- Transcriptional errors stratified by gene length: protein-coding genes were divided into four quantiles based on their length (from shortest to longest) and then Transcription Error rate was calculated for each quantile. Two sample t-test was used to determine if there was statistical difference between N2 and PRDE1 samples, with p-value=0.05 considered significant.

## STATISTICAL ANALYSIS

### Statistical analysis (RNA-sequencing data):

Anova with posthoc Tukey HSD was conducted to compare distribution of Gene Length in the Differential Expressed Genes, with adjusted p-value <0.05 was considered significant. Wilcoxon test was used to compare the expression of the piRNAs, difference between the means was considered significant if the p-value <0.05. Hypergeometric test was done using the phyper function in R^29^, overlap was considered significant if p-value <0.05. Pearson’s Chi-squared test with Yates’ correction was used to determine if the number of up- or down-regulated splicing events during heat shock differed between the N2 and PRDE1 strains (p-value <0.05). All the statistical analyses were done in R^29^.

### Statistical analysis (qPCR data and phenotypes):

The data were analyzed by using Student t-test (two-tailed unpaired t-test) or 2 way-ANOVA (Fisher’s LSD test; GraphPad Prism software) as described in respective figure legends. P values are indicated as follows: ∗p < 0.05; ∗∗p < 0.01; ∗∗∗p < 0.001.

### Statistical analysis (other):

The statistical analyses for the remaining datasets are indicated in the Figure Legends.

## Supplementary Material

Supplement 1

## Figures and Tables

**Method Fig 1.**
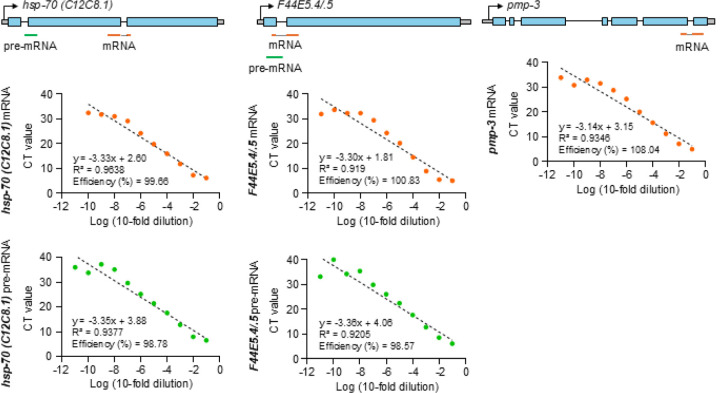
Standard curves for hsp–70 mRNA and pre–mRNA and pmp–3

**Table 1.** Strain information

Strain name	Genotype		Source	Note
N2	Bristol N2; Wild-type		CGC	
sx2499	*prde-1(mj207) V*		CGC	
PS3551	*hsf-1 (sy441) I*		CGC	
FLAG::HSF-1	*3X flag::hsf-1 I*	CRISPR/Cas-9	Prahlad Lab	
FLAG::HSF-1 (*sy441*)	*3X flag::hsf-1(sy441) I*	CRISPR/Cas-9	Prahlad Lab	
WM274	*prg-1(tm872) I; neSi14 [FLAG::prg-1 + Cbr-unc-119(+)] II; unc-119(ed3) III.*		CGC	
JDW10	wrdSi3 [sun-1p::TIR1::F2A::mTagBFP2::AID*::NLS::tbb-2 3’UTR] II		CGC	
VP27	*prde-1::AID (syb7464) V*	CRISPR/Cas-9	Prahlad Lab/SunyBiotech	
JDW10 ;VP27	wrdSi3 [sun-1p::TIR1::F2A::mTagBFP2::AID*::NLS::tbb-2 3’UTR] II; prde-1::AID (syb7464) V	Cross	Prahlad Lab	JDW10 was selecte d by picking BFP positive worms
VP01	*C12C8.1::FLAG (syb2141) I*	CRISPR/Cas-9	Prahlad Lab / SunyBiotech	
VP01; sx2499	*C12C8.1::FLAG (syb2141) I; prde-1(mj207) V*	Cross	Prahlad Lab	

**Table 2.** Primer information

Experiments	Name	Forward	Reverse
genotype	VP01	CTCAAGCTGATATTGATAGA	AGCTAATTGTACAGAGAAAA
	VP27 (Outside AID insertion)	CGTCAAATATGTTGCGCATT	AAAATTCATTCCGCAGCAAC
	VP27 (Inside AID insertion)	GGTTTCCTGCCAAAAATCAA	AAAATTCATTCCGCAGCAAC
	sx2499	GAAATCGCAAAGGACACGAT	CGGCTTGTCACACACAAGAT
Mutliple experiments	*hsp-70 (C12C8.1)* pre-mRNA	TTTATTTTCCGTTTTCAGGTTGA	CCACGTAGGAAGGCGTAGTC
	*hsp-70 (C12C8.1)* mRNA	TTGGTTGGGGGATCAACTCG	GAGCAGTTGAGGTCCTTCCC
	*F44E5.4/.5* pre-mRNA	CGGTACTACGTACTCGTGTGTTG	TGTTATTAAAATTGAAAATGCCAAA
	*F44E5.4/.5* mRNA	CTATCAGAATGGAAAGGTTGAG	TCTTTCCGTATCTGTGAATGCC
	*pmp-3* mRNA	TAGAGTCAAGGGTCGCAGTG	ATCGGCACCAAGGAAACTGG
PCR for generating Standard Curve	*hsp-70 (C12C8.1) (*cDNA)	CCCGTTGTTGAGGTTGAAGT	CGGCAGCTGATACATTGAGA
	*hsp-70 (C12C8.1)* (gDNA)	ACGTACTCATGTGTCGGTAT	CGGCAGCTGATACATTGAGA
	*F44E5.4/.5* (cDNA)	CTATCAGAATGGAAAGGTTGAG	TCACAAGCAGTTCGGAGACG
	*F44E5.4/.5* (gDNA)	TAAAAGGGCTGGGATTCGGG	TCACAAGCAGTTCGGAGACG
	*pmp-3* (cDNA)	TGGAGACAGTGGATGTGGAA	GAAAGAACTGTATCGGCACCA
RIP-RT-qPCR	*bath-45* mRNA	ACGATAGGCCTTTTCCAATG	GAGCTGCCTTTTCCAAATTC
ChIP-qPCR	*hsp-70 (C12C8.1)* TSS	ACGTACTCATGTGTCGGTAT	TCTTCTTCCAGTTTACATAATCCT
	*hsp-70 (C12C8.1)* End	ACAATTCGCAATGAGAAGGGACG	GCATCTTCTGCTGATAACAGTGATC
	*F44E5.4/.5* TSS	TAAAAGGGCTGGGATTCGGG	ACCGAGGTCGATACCAATAGC
	*F44E5.4/.5* End	TTGATGAAACACTTCGTTGGTTGG	TCCAGCAGTTCCAGGATTTC
RT-qPCR	*T08G5.11*	CGATCAAGAAGGCGGAATCC	TCAACTCGAGACTGCCACAC
	*H27M09.6*	GCGATCACAAAGGCGGAATC	TCAACTCGAGACTGCCACAC
	*F08H9.10*	ATCAAGAAGGCGGAATCCCC	TCAACTCGAGACTGCCACAT
	*sup-6*	TGATCATGAAGACGGAATCCCC	TCAACTCGAGACTGCCACAC
	*sup-39*	GCCTACCCATTGCACTTTTGG	GCAGCCCCGATACGCAAAA

* Genotyping of sx2499 mutant was verified through digestion with MuCl.

**Table 3.** Primer information (small RNA)

Experiments	Name	Sequence	Targeting 22G RNA sequence
22G RNA FISH	*F44E5.4/.5* 22G RNA	ATCAGTTTGTCAATCAATTCTC	GAGAATTGATTGACAAACTGAT
sRNA RT-qPCR	*hsp-70 (C12C8.1)* **Stem-loop (SL)**	CTCAACTGGTGTCGTGGAGTCGGCAATTCAGTTGAGTCTATTCG	GCACGTGTGATTTTCGAATAGA
	*hsp-70 (C12C8.1)* 22G RNA **Forward**	ACACTCCAGCTGGGGCACGTGTGATTTTCG	
	*F44E5.4/.5* **Stem-loop (SL)**	CTCAACTGGTGTCGTGGAGTCGGCAATTCAGTTGAGATCAGTTT	GAGAATTGATTGACAAACTGAT
	*F44E5.4/.5* 22G RNA **Forward**	ACACTCCAGCTGGGGAGAATTGATTGACAA	
	Universal **Reverse** primer	CTCAAGTGTCGTGGAGTCGGCAA	
Synthetic RNA	21ur-2348	UGAUGUCCCAUGUCGCAAUUC	

* synthetic 21ur-2348 RNA has 5’ phosphorylation at 5’ end and 2’ O-methyl bases at 3’ end

**Table 4.** UMI total family size 1 (singletons)

sample	family_size	number of reads	proportion
N2 HS 1	1	2.72E+07	0.532974
N2 HS 2	1	3.34E+07	0.445124
N2 HS 3	1	2.79E+07	0.54433
PRDE1 HS 1	1	3.17E+07	0.510223
PRDE1 HS 2	1	3.21E+07	0.500472
PRDE1 HS 3	1	3.20E+07	0.498199

## Data Availability

The data for this study have been deposited in the European Nucleotide Archive (ENA) at EMBL-EBI under accession numbers PRJEB101605 (https://www.ebi.ac.uk/ena/browser/view/PRJEB101605), PRJEB101594 (https://www.ebi.ac.uk/ena/browser/view/PRJEB101594), PRJEB101181(https://www.ebi.ac.uk/ena/browser/view/PRJEB101181) .
