# piRNAs safeguard splicing and RNA fidelity during heat shock

**DOI:** 10.1101/2025.10.27.684940

**Published:** 2025-12-10

**Authors:** Sehee Min, Johnny Cruz-Corchado, Veena Prahlad

**Affiliations:** Department of Cell Stress Biology, Roswell Park Comprehensive Cancer Center, Elm and Carlton Streets, Buffalo, NY 14263

**Keywords:** piRNA, heat shock, *hsp*, splicing, nonsense-mediated decay (NMD), endo-siRNAs, transcript fidelity, RNA quality control, protein homeostasis, *C. elegans*

## Abstract

Piwi-interacting RNAs (piRNAs) silence transposons and foreign sequences; yet, for poorly understood reasons, they also extensively target endogenous mRNAs. Here, we report a previously unappreciated role for the *Caenorhabditis elegans* piRNA pathway in RNA quality control: during heat shock, piRNAs generate endogenous antisense RNAs (endo-siRNAs) that associate with RNA Polymerase II and nascent stress-induced RNAs to delay splicing when the spliceosome is compromised, minimizing transcript errors. In the absence of piRNAs, nascent transcripts undergo premature splicing, accumulate errors, and activate nonsense-mediated decay (NMD) to limit the production of aberrant proteins. We propose that by regulating splicing, the piRNA pathway acts alongside NMD as a quality-control system, safeguarding RNA fidelity and proteome integrity with genome defense.

## Introduction

Piwi-interacting RNAs (piRNAs) are small, single-stranded RNA guides for PIWI Argonaute proteins that suppress transposable elements and foreign RNAs through transcriptional and post-transcriptional silencing(1–10). Yet, across animal species, a large proportion of piRNAs target endogenous genes, suggesting that the piRNA pathway also plays evolutionarily ancient and conserved roles in regulating gene expression(1–7, 11–15). However, their endogenous functions remain mostly unknown. In *Caenorhabditis elegans*, piRNAs associate with PRG-1 (the *C. elegans* PIWI protein) and can target, with high mismatch tolerance, nearly all germline RNAs, including ectopic sequences such as GFP transgenes(1, 2, 16, 17). piRNA-targeting initiates the production of secondary antisense small endogenous silencing RNAs (endo-siRNAs, or 22G-RNAs) templated from the piRNA-targeted mRNAs by RNA-dependent RNA polymerases (RdRPs)(18–20). These 22G-RNAs recruit WAGO Argonaute proteins to enforce co- and post-transcriptional silencing. Self-RNAs are spared this promiscuous silencing through their interactions with another Argonaute protein, CSR-1, guided by 22G-RNAs generated from actively translated transcripts independently of piRNAs(21, 22). These CSR-1-associated 22G-RNAs, thus, act as molecular engrams that recognize expressed ‘self’ genes to prevent their silencing. However, this leaves unexplained how inducible genes— normally absent and thus lacking CSR-1–22G-RNA guides, but whose expression is necessary for organismal function— escape piRNA-mediated silencing.

To address this question, we conducted RNA-sequencing to profile mRNAs, piRNAs, and 22G-RNAs after subjecting *C. elegans* to heat shock—a stimulus which induces the abrupt and massive expression of heat shock protein (*hsp*) and other mRNAs necessary to survive stress, but which are typically undetectable in unstressed cells (23–25). We found that instead of escaping piRNA surveillance, surprisingly, the piRNA system targeted *hsps* and other stress-induced mRNAs, producing robust endo-siRNAs (22G-RNAs) antisense to these transcripts. Therefore, to understand the functional role of this targeting, we investigated the effects of the piRNA/22G-RNA system on nascent transcript synthesis, maturation, turnover, and fidelity, using *hsps* as model transcripts, and 5-ethynyluridine (EU) pulse-chase labeling, Adapted Photoactivatable Ribonucleoside-Enhanced Crosslinking and Immunoprecipitation (PAR-CLIP), chromatin fractionation, and Unique Molecular Identifier- (UMIs)-labelling (26–30) to analyze transcript errors. We found that the piRNA system biases splicing decisions, favoring post- rather than co-transcriptional splicing. This restrains splicing during heat shock when the spliceosome is compromised, increasing the pool of unspliced pre-mRNA to be processed during stress recovery, and limiting transcript errors. We show that the piRNA-generated 22G-RNAs enforce splicing delays by interacting with Pol II and nascent RNA, promoting chromatin retention of mature transcripts. In contrast, in the absence of piRNAs and associated 22G-RNAs, splicing is accelerated, resulting in genome-wide misregulation of long, intron-rich genes, the accumulation of transcript errors, and the increased activation of the nonsense-mediated decay (NMD) pathway to prevent protein misfolding and ensure survival. Based on these studies, we propose that the pervasive targeting of endogenous genes by the piRNA system serves a previously unrecognized but critical role in RNA quality control.

## Results

### Heat-shock induced *hsp* and other mRNAs are targeted by the piRNA pathway

**Figure 1.**
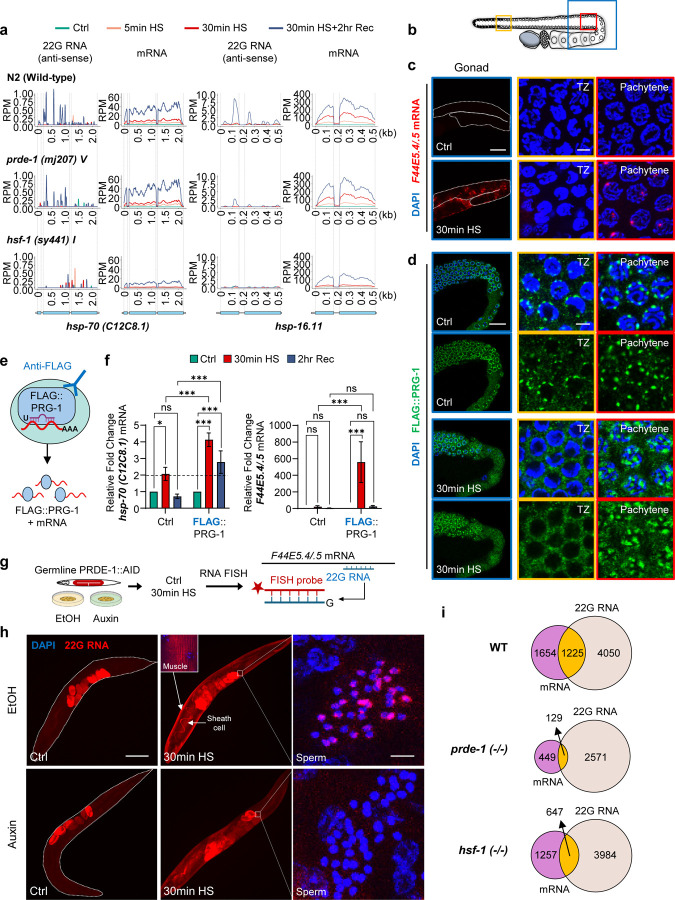
Piwi-interacting RNAs (piRNAs) target *hsp* mRNAs upon heat shock. **a.** Metaprofile plots showing normalized antisense 22G-RNA reads (RPM), and mRNA reads (RPM) across *hsp* mRNAs in wild-type (WT, *C. elegans* wild isolate, var Bristol), *prde-1(mj207) V,* and *hsf-1(sy441) I* mutants. **Top**: conditions of heat shock. **Bottom**: Schematic of *hsp* genes analyzed. **b-d.** Spatial localization of *hsp-70* mRNA and PIWI (PRG-1) in the WT germlines. **(b)** Schematic of gonad regions in **c** and **d**: Blue box, Gonad region shown. Orange box, transition zone (TZ). Red box, Late pachytene. Representative confocal images of each region showing **(c)** *hsp-70 (F44E5.4/.5)* mRNA localized using RNA Fluorescence *in situ* hybridization (RNA FISH; Projected image), and **(d)** PIWI (FLAG::PRG-1) by immunostaining with anti-FLAG antibody (Z-section). Germlines from non-heat-shocked (Ctrl) and heat-shock-exposed (30min HS) animals are shown. Red: *hsp-70 (F44E5.4/.5)* mRNA. Green: FLAG::PRG-1. Blue: DNA staining with DAPI. Scale bars, c: 50 μm (Gonad), and 3 μm (TZ, Pachytene). Scale bars, d: 20 μm (Gonad), and 3 μm (TZ, Pachytene). **e, f.** Native RNA immunoprecipitation (RIP) to probe *hsp* mRNA’s association with PIWI (FLAG::PRG-1). **(e)** Experiment Schematic. **(f)** Relative fold change (RT-qPCR) in *hsp-70s* (*C12C8.1* and *F44E5.4/.5*) mRNAs that immunoprecipitated with PIWI. *hsp* mRNA levels were normalized to *pmp-*3 (input) and are shown relative to non-heat-shocked animals. Ctrl: wild-type animals with no FLAG-tagged proteins were negative controls. *bath-45* served as positive control (Suppl. Fig 2b). Legend: Treatment conditions. Bars: mean value. Error bars: standard error. n = 3–5 biologically independent experiments. Statistical analysis: 2-way ANOVA. **g, h.** Germline depletion of PRDE-1 using the auxin-inducible degron (AID) system followed by RNA FISH to detect 22G-RNAs antisense to *hsp-70 (F44E5.4/.5).* **(g)** Experiment schematic: EtOH: Vehicle control. Probe design shown. **(h)** Representative micrographs (projected confocal Z-sections) showing RNA FISH of 22G-RNA under control conditions (EtOH) and upon PRDE-1 depletion (auxin). Red: 22G-RNA. Blue: DAPI stained DNA. Expression in muscle (arrow, box) and gonad sheath cells (arrow), and in sperm (highlighted). Embryos staining is non-specific. Scale bars: 100 μm (Ctrl, 30min HS), and 3 μm (Sperm). **i.** Venn diagrams showing overlap between differentially expressed mRNAs and 22G-RNAs upon 30 minutes heat shock in WT, *prde-1* and *hsf-1* mutants.

To understand the role of the piRNA system in regulating inducible genes, we performed small RNA (sRNA-seq) and mRNA (paired-end, strand-specific, polyA-selected) sequencing from synchronized day-one adults exposed to mild (5 mins, 34°C) or strong (30 mins, 34°C) heat shock (Suppl. Fig 1a; Suppl. Tables 1, 2). Animals were also analyzed after two hours of recovery at 20°C, when normal physiological functions largely resume(31). In parallel, to identify piRNA-dependent effects, we sequenced *prde-1* mutants, deficient in piRNA biogenesis(32, 33) (Suppl. Fig. 1a; Suppl. Tables 1, 2). As expected, heat shock induced global changes in mRNA (Suppl. Fig 1b), including the massive upregulation of *hsp* genes (Fig 1a; Suppl. Fig 1c, d *mRNA*). Surprisingly, in wild-type animals, heat shock also produced 22G-RNAs antisense to the *hsp* mRNAs (Fig 1a; Suppl. Fig 1c, d *22G-RNA*), suggesting that rather than escaping piRNA surveillance, *hsp* mRNAs may be targeted by the piRNA system. Like the *hsp* mRNAs, these antisense 22G-RNAs also appeared within 5 minutes of heat shock and peaked during recovery (Fig 1a; Suppl. Fig 1c, d *22G-RNA*). Consistently, by 30 minutes of heat shock, several piRNAs were also modestly but significantly induced (Suppl. Fig 1e, f). In *prde-1* mutants, *hsp* induction upon heat shock appeared unperturbed, but many fewer 22G-RNAs antisense to *hsp* mRNAs were produced (Fig 1a; Suppl. Fig 1c, d *mRNA; 22G-RNA*), indicating that these 22G-RNAs were generated because of piRNA-targeting.

Because piRNA targeting of mRNAs generates 22G-RNAs through the activities of the PIWI Argonaute PRG-1 and RdRPs that reside in perinuclear condensates (nuage or germ granules) of the germline(16, 19), to confirm these unexpected results, we tested whether *hsp* mRNAs interact with PRG-1. (34, 35). First, we determined whether *hsps* are also induced in the germline upon heat shock, by conducting RNA Fluorescence *in situ* hybridization (RNA FISH; Fig 1b, c; Suppl. Fig 2a) to localize *hsp-70s (C12C8.1* and *F44E5.4/.5)*. Heat shock induced the expression of *hsp-70* mRNAs in the late pachytene nuclei (and only these nuclei) of the germline. Moreover, although PRG-1 localization is known to dissolve upon exposure to increased temperatures and indeed does so in regions such as the transition zone (TZ; Fig 1d), PRG-1 remained in perinuclear condensates upon heat shock in the pachytene nuclei (Fig. 1d; compare transition zone, TZ, where *hsp* mRNAs are absent, with Pachytene), supporting potential *hsp-70-*PRG-1 interactions. Next, we immunoprecipitated PRG-1 under control, heat-shock, and recovery conditions from animals expressing FLAG-tagged PRG-1 to determine whether *hsp-70 (C12C8.1* and *F44E5.4/.5)* mRNAs associated with PRG-1 (native RNA immunoprecipitation, RIP, followed by RT–qPCR; Fig 1e, f). Non-FLAG animals were used to account for non-specific interactions of *hsp* mRNAs, and *bath-45,* a known piRNA target, served as a positive control (Fig 1e, f; Suppl. Fig 2b). These experiments also indicated that *hsp-70s* interacted with PRG-1 upon heat shock, and *hsp-70 (C12C8.1)*, but not *hsp-70 (F44E5.4/.5)* remained associated for a longer duration, even during recovery (Fig 1e, f; Suppl. Fig 2b). Finally, and most convincing, we depleted PRDE-1 specifically in the germline using an auxin-inducible degron (AID) system, and found that this abolished 22G-RNAs presence throughout the animal (RNA FISH), demonstrating that 22G-RNAs originate in the germline through piRNA targeting (Fig 1g, h; embryos show non-specific staining). Surprisingly, despite their germline origin, the 22G-RNAs were present throughout the animal, in somatic cells that expressed *hsp* mRNA (e.g. muscle, somatic sheath cells), as well as in the germline cells such as sperm, which do not (RNA FISH; Fig 1g, h; Suppl. Fig 2c). Upon germline depletion of PRDE-1, 22G-RNA levels were starkly decreased in somatic and germline cells (Fig 1g, h), while *hsp-70* mRNA continued to be expressed (Suppl. Fig 2c). Together, these findings confirmed that *hsp* mRNAs were subject to piRNA surveillance, actively targeted by the canonical piRNA pathway in the germline, and produced 22G-RNAs that propagated systemically across tissues.

Further evidence that the *hsp* genes induced by heat shock were templating antisense 22G-RNA production came from profiling mRNAs and sRNAs in a mutant in heat shock factor 1 (HSF1; HSF-1 in *C. elegans*), the transcription factor responsible for *hsp* expression upon heat shock. This mutant, *hsf-1 (sy441)* is deficient in heat-induced activity but expresses higher basal levels of *hsp* mRNA(31, 36). Yet *hsf-1* mutants failed to produce 22G-RNAs antisense to *hsp* genes (Fig 1a; Suppl. Fig 1c, d). Surprisingly, even the few *hsp* mRNAs, like *hsp-70 (F44E5.4/.5)*, that did accumulate upon heat shock in *hsf-1 (sy441)* mutants, did not template 22G-RNAs (Suppl. Fig 1c, d), perhaps suggesting a broader role for HSF-1 in piRNA-dependent 22G-RNA production. piRNA surveillance was not limited to *hsps*, but extended to other heat-shock-regulated genes: ~15% of differentially expressed genes (DEGs) upon a 5-minute heat-shock and ~40% by 30 minutes templated 22G-RNA production, and both the numbers of DEGs, and DEG-derived 22G-RNAs were reduced in *prde-1* and *hsf-1* mutants (Fig 1i; Suppl. Fig 2d; Suppl. Table 3). 22G-RNA function is determined by its Argonaute partner; however, the 22G-RNAs that targeted *hsp* mRNAs were ‘unclassified’ and not previously identified in association with known *C. elegans* Argonaute proteins (Suppl. Fig 3; Suppl. Table 4). However, other DEGs did template 22G-RNAs that had been previously identified as guide RNAs for WAGO, CSR-1, and other Argonaute proteins (Suppl. Table 4). WAGO-associated 22G-RNAs that were induced upon 30 minutes of heat shock exposure were piRNA-dependent, as apparent by their decreased levels in *prde-1* mutants, and in wild-type they increased as their target mRNAs decreased, consistent with their known roles in post-transcriptional or co-transcriptional silencing(8, 37) (Suppl. Fig 3a, b). CSR-1 22G-RNAs declined upon heat shock (perhaps due to translational attenuation), as their target mRNAs rose in both wild type and *prde-1* mutants, consistent with their mostly piRNA-independent production (Suppl. Fig 3a, b). Unclassified 22G-RNAs, meaning those whose Argonaute partners had not been previously identified, continued to increase in a *hsf-1-* and *prde-1*-dependent manner, even during recovery from heat shock, as did their complementary mRNAs (Suppl. Fig 3a, b). The effects were dynamic and reversible during recovery from stress (Suppl. Fig 2d (Rec); Suppl. Fig 3a, b), and the mRNAs that templated 22G-RNA production showed distinct GO enrichments (Suppl. Fig 4; Suppl. Table 5).

Together, these results suggested that the broad piRNA-dependent and independent 22G-RNA expression that was triggered by heat shock likely contributes to the pervasive transcriptome-wide remodeling(8, 37) universally associated with this stress-induced transcription program.

### The piRNA-pathway regulates post-transcriptional splicing.

**Figure 2.**
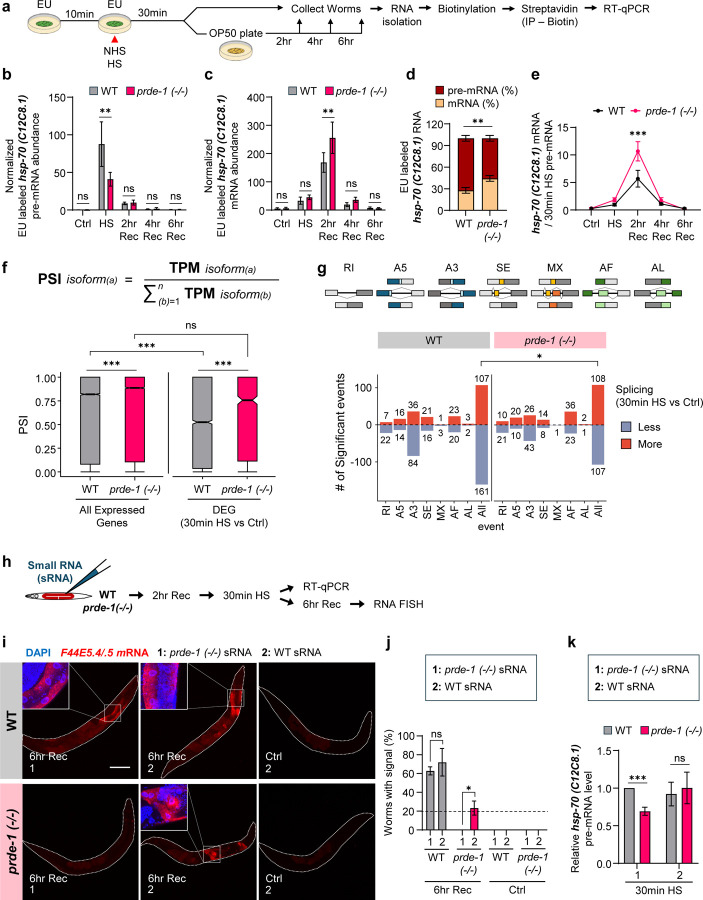
piRNAs delay *hsp* mRNAs splicing. **a-e.** Metabolic pulse-chase labeling with EU and quantification of *hsp* RNA. **(a)** Experiment schematic: animals were fed EU-mixed bacteria 10 minutes before heat shock (pre-loading), heat shocked for 30 minutes (pulse-labeling nascent RNA), and moved onto unlabeled bacterial plates (chase). The EU-labeled nascent RNA was isolated from NHS animals (Ctrl), heat-shocked animals immediately after 30 min heat-shock (HS), and after 2,4, and 6 hrs post-heat shock recovery (Rec), biotin-conjugated, and immunoprecipitated. Normalized absolute RNA abundance was quantified by RT-qPCR (see methods; *pmp-3* mRNA used to normalize for variability in labeling). EU-labeled *hsp-70 (C12C8.1)* **(b)** pre-mRNA and **(c)** mRNA abundance. Y-axis: Normalized abundance. X-axis: Treatments. Note EU-labeled mRNA is derived from the splicing of EU-labeled pre-mRNA. **(d)** Percentages of pre-mRNA and mRNA in total EU-labeled RNA after 30 min heat shock. **(e)** Relative amounts of EU-labeled spliced mRNAs produced from the nascent EU-labeled unspliced pre-mRNA. Values normalized to *pmp-3*. Y-axis: Relative abundance. X-axis: Treatment. Bars (b-d) and line graph (e): mean value. Error bars: standard error. (b, c, e) n = 5–13 biologically independent experiments. Statistical analysis: 2 way-ANOVA; (d) n = 14 biologically independent experiments. Statistical analysis: two-tailed unpaired t-test. **f.** Genes differentially regulated by heat shock in WT, but not *prde-1* animals, are enriched for alternative spliced isoforms. **Top**: Calculation of PSI “percent spliced in” values. **Bottom**: Alternative spliced isoforms, as reflected by PSI values, in all expressed genes, and in differentially expressed genes (p.adj <0.05) in wild-type and *prde-1* animals. Boxplots: median value depicted. Error bars: standard error. Statistical analysis: two-tailed unpaired t-test. **g.** Alternative splicing (AS) events in WT and *prde-1* animals upon heat shock. **Top**: AS events, retained intron (RI), alternative 5’/3’ Splice Sites (A5/A3), skipped exon (SE), mutually exclusive exons (MX), and alternative first and last exons (AF/AL). **Bottom**: Number of AS events amongst differentially expressed genes (HS vs NHS condition; p.adj <0.05) in WT and *prde-1* mutants. Bars: Number of events (splicing increase or decrease relative to NHS condition). Statistical analysis: Chi-square analysis. **h-j.** Microinjection of small RNA fractions isolated from WT animals rescues *hsp-*70 fate. **(h)** Experiment schematic: small RNA fractions purified from heat-shocked WT and *prde-1* animals were injected into WT and *prde-1* animals shortly before heat-shock exposure, and animals were probed for *hsp-70 (F44E5.4/.5)* 6 hours post-heat shock. Non-heat-shocked animals were injected to control for effects of injection alone. **(i)** RNA FISH of *hsp-70 (F44E5.4/.5)* in WT and *prde-1* animals 6 hours following heat shock or under NHS conditions. Injection mix (box) and treatment indicated. Red: *hsp-70 (F544E5.4/.5)* mRNA. Blue: DNA staining with DAPI. Scale bars: 100 μm. **(j)** Quantification of results. Y-axis: Percentage of injected animals positive for *hsp-70*. X-axis: Injection mix, strains and treatment. Bars: mean value. Error bars: standard error. n = 17–22 worms (6hr Rec), 6–10 worms (Ctrl). Statistical analysis: Fisher’s exact test. **k.** Microinjection of small RNA fractions isolated from heat-shocked WT animals rescues *hsp-*70 pre-mRNA levels. Normalized RT-qPCR of relative fold change of *hsp-70* (*C12C8.1*) pre-mRNAs following sRNA injection. Y-axis: *hsp-70* pre-mRNA levels, normalized to *pmp-3*, relative to heat-shocked wild-type animals injected with small RNAs from *prde-1* mutants. X-axis: Injection mix (see box in Fig 2g). Bars: mean value. Error bars: standard error. n = 6 biologically independent experiments. Statistical analysis: two-tailed unpaired t-test.

Given the known roles of piRNAs in post- and co-transcriptional silencing, we suspected that piRNA targeting of *hsp* and other stress-induced genes functioned to clear transcripts during recovery from heat shock. If so, *hsp* mRNAs should persist longer after stress in *prde-1* mutants. Instead, RT-qPCR (Suppl. Fig 5a–c) and RNA-FISH (Suppl. Fig 5d) revealed the opposite: the steady state abundance of *hsp* mRNAs declined more rapidly in *prde-1* mutants compared to wild type. We examined whether decreased RNA Polymerase II (Pol II) binding at *hsp* genes(38), or altered transcription duration, explained this progressive decline. But this was also not the case: chromatin immunoprecipitation followed by qPCR (ChIP-qPCR) showed that Pol II levels at *hsp* gene transcription start sites (TSS), and its return to basal levels by 2 hours post-heat shock, were similar in *prde-1* and wild-type animals (Suppl. Fig 5e, f). Therefore, to understand the role of the piRNA pathway’s targeting of *hsps* and other heat-shock genes, we directly assessed *hsp* mRNA dynamics in the absence and presence of the piRNA pathway by tracking their synthesis and turnover using a pulse-chase metabolic labeling approach: i.e., we labeled the heat-shock induced pre-mRNA with 5-ethynyluridine (EU)(39, 40) provided to the animals only during heat shock exposure, and tracked the fate of this RNA by absolute RT–qPCR using exon–intron (pre-mRNA) and exon–exon (mature mRNA) primers during recovery from heat shock (Fig 2a; see methods for experimental details).

These studies revealed that in the absence of piRNAs, *hsp* splicing or maturation occurred at an accelerated pace, resulting in a precocious increase in nascent mRNAs and a concomitant decrease in pre-mRNA pools, whereas the presence of piRNAs in wild-type animals restrained splicing. The evidence for this was as follows. First, consistent with similar Pol II occupancy at *hsp* genes, *prde-1* synthesized similar total amounts of RNA as wild-type animals upon heat shock (Suppl. Fig 5g, h). Despite this, *prde-1* mutants had significantly reduced amounts of nascent (EU-labeled) pre-mRNA by the end of the 30-minute heat shock (Fig 2b; Suppl. Fig 5i), but, on the other hand, accumulated significantly more EU-containing spliced mRNA by 2 hours following heat shock (Fig 2c; Suppl. Fig 5j). Next, the amounts of unspliced nascent pre-mRNA present at the end of heat shock exposure, calculated as the ratio of EU-labeled pre-mRNA over total EU-labeled RNA(41), was higher in wild-type animals expressing piRNAs/22G-RNAs compared to *prde-1*: ~75% of nascent *hsp-70 (C12C8.1)* transcripts, which contain two introns, remained as pre-mRNA to be spliced post-transcriptionally during recovery in wild-type animals (Fig 2d), whereas only ~57% persisted unspliced in *prde-1* mutants (Fig 2d). Thus, the piRNA pathway biased the decision between co- and post-transcriptional splicing toward delayed splicing. Finally, this remaining *hsp-70 (C12C8.1)* was also spliced more rapidly in *prde-1* animals: within 2 hours of recovery, dramatically more EU-containing spliced mRNAs were produced from the nascent EU-labeled unspliced pre-mRNA (Fig 2e). The other inducible *hsp-70 (F44E5.4/.5)*, contained only one intron, and was mainly co-transcriptionally spliced in both wild-type and *prde-1* animals (Suppl. Fig 5k). Still, the post-transcriptional splicing of the residual *hsp-70 (F44E5.4/.5)* proceeded markedly faster in *prde-1* mutants than in wild-type animals (Suppl. Fig 5l). This was also true for the small heat shock protein (*hsp-16.11*; Suppl. Fig 5m).

Besides the co- and/or post-transcriptional splicing of *hsp* mRNAs, the piRNA system appeared to broadly regulate transcriptome-wide splicing decisions: in wild-type animals compared to *prde-1* mutants, heat shock-DEGs (p.adj <0.05) expressed more alternatively spliced (AS) isoforms, as indicated by their much lower “percent spliced in” (PSI or Ψ)(42, 43) values (Fig 2f). In addition, 540 transcripts showed significant dPSI (differential PSI; p <0.1) values, compared to only 139 transcripts in *prde-1* (Suppl. Table 6). Moreover, for seven representative AS events(44, 45) — retained intron (RI), alternative 5’/3’ Splice Sites (A5/A3), skipped exon (SE), mutually exclusive exons (MX), and alternative first and last exons (AF/AL) (Fig 2j) — mRNAs differentially expressed upon heat shock in wild-type animals were significantly less spliced, retaining more introns compared to *prde-1* (Fig 2g; Suppl. Table 7). In further support of the role of the piRNA system in alternative splicing, nearly 40% (125/308) of DEGs that produced AS transcripts upon heat shock in wild-type animals also templated 22G-RNAs, while only 12% (35/280) did so, in *prde-1* (Suppl. Fig 6a). These AS mRNAs were enriched in developmental processes, and in particular for neuronal functions known to rely more on alternative splicing (Suppl. Fig 6b; Suppl. Table 8). This suggested that their delayed splicing may be important for the restoration of developmental processes during recovery from heat shock, and accordingly, heat-shocked *prde-1* mothers showed a marked delay in generating viable embryos post-heat shock (Suppl. Fig 6c, d). These results showed that the piRNA pathway, perhaps by virtue of the sequence complementarity of the 22G-RNAs it produced, impacted transcriptome wide splicing decisions upon heat shock.

To determine whether the splicing decisions were dynamically controlled by the piRNA system, we investigated whether we could rescue *hsp* RNA dynamics by microinjecting small RNAs obtained from heat-shocked wild-type animals into *prde-1* animals shortly before subjecting them to heat-shock exposure (Fig 2h). This was indeed the case: when *prde-1* animals were microinjected with small RNA fraction (<200nt) obtained from heat-shocked wild-type animals, but not with small RNA fractions from heat-shocked *prde-1* animals, both the persistence of *hsp* mRNA (Fig 2i, j), and the levels of *hsp* pre-mRNA were partially rescued (Fig 2k). Small RNA injections *per* se did not induce or enhance *hsp* expression (Fig 2i, j).

Thus, together, the results from pulse-chase metabolic labeling, transcriptome-wide alterations in AS, and microinjection rescue experiments suggested that the piRNA/22G-RNA pathway functioned dynamically, to alter splicing decisions to preserve mRNA expression during heat-shock recovery by restraining splicing and maintaining a pool of pre-mRNAs for delayed processing.

### 22G-RNAs act through a ‘telescripting’-like mechanism to protect long gene expression.

Mechanistically piRNA/22G-RNAs could regulate splicing indirectly, through their known interactions with chromatin(8, 34, 46). Alternatively, and consistent with their ability to rescue *hsp* expression dynamics, and the rapid expression of *hsp* genes that occurs upon heat shock, the piRNA/22G-RNAs could regulate splicing directly by acting on target pre-mRNAs at the transcription complex to bias splicing decisions(47). Therefore, to understand how 22G-RNAs regulate splicing, we adapted Photoactivatable Ribonucleoside-Enhanced Crosslinking and Immunoprecipitation (PAR-CLIP) to test whether these small RNAs are associated with Pol II at the transcription complex. In addition, because co-transcriptional splicing occurs during Pol II elongation, but the mechanisms that regulate the timing, transition, and completion of post-transcriptional splicing are still unclear(48, 49), we immunoprecipitated Pol II immediately at 30 minutes upon heat shock, when transcription was still in process, or after 2 hours of recovery from heat shock, when Pol II was no longer engaged with the *hsp* locus (ChIP-qPCR; Suppl. Fig 5e, f), but spliced mRNAs accumulated in cells (Fig 2c; Suppl. Fig 5j), and determined the levels of nascent *hsp* mRNA or pre-mRNA that remained associated with Pol II along with the 22G-RNAs.

**Figure 3.**
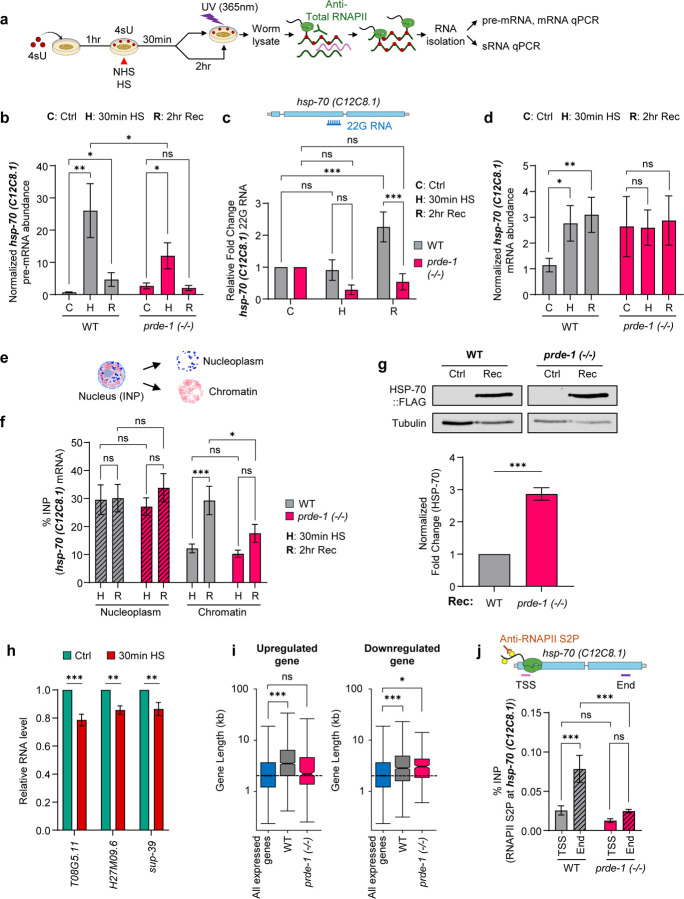
Antisense 22G-RNAs associated with RNA Polymerase II at the transcription complex to modulate post-transcriptional splicing. **a-d.** Modified PAR-CLIP to assess 22G-RNAs’ role in splicing. **(a)** Experiment schematic: Animals were fed 4sU-mixed bacteria, subjected to heat shock, exposed to UV (365 nm) either immediately or after 2 hours of recovery, RNA Pol II was immunoprecipitated, *hsp* mRNA and pre-mRNA were quantified by computing absolute RNA abundance (RT-qPCR and standard curves; *pmp-3* mRNA used to normalize for variability in IP amounts across samples and experiments) and 22G-RNA quantified as relative fold change using stem loop PCR (design shown). See Suppl. Fig 7c for details. Amounts of **(b)** *hsp-70 (C12C8.1)* pre-mRNA, **(c)** 22G-RNA, and **(d)** *hsp-70 (C12C8.1)* mRNA that cross-linked to, and immunoprecipitated with Pol II. Y-axis: Normalized or relative abundance. X-axis: Treatment conditions (see top legend). Bars: mean value. Error bars: standard error. (b) n = 8–13 biologically independent experiments. Statistical analysis: two-tailed unpaired t-test (within strain) and 2-way ANOVA (between strains); (c) n = 5–6 biologically independent experiments. Statistical analysis: 2-way ANOVA; (d) n = 5–10 biologically independent experiments. Statistical analysis: 2-way ANOVA. **e, f.** Nuclear fractionation to assess chromatin retention of *hsp-70* RNA. **(e)** Experiment schematic. Nuclei were isolated, fractionated to obtain nucleoplasm and chromatin, and enrichment of *hsp-70* mRNA in both fractions was determined. **(f)** Fold enrichment over input (nuclei) of *hsp-70 (C12C8.1)* mRNA in respective fractions. Y-axis: % input (total nucleus). X-axis: Nuclear fraction, strain, and treatment. Bars: mean value. Error bars: standard error. n = 9–10 biologically independent experiments. Statistical analysis: 2-way ANOVA. **g.** HSP-70 protein levels in WT and *prde-1.* **Top:** Representative western blot image showing HSP-70(C12C8.1)::FLAG in NHS (Ctrl) or after heat-shock in WT and *prde-1* animals expressing endogenous *flag*-tagged *hsp-70*. Animals harvested 3 hrs following HS (Rec) to allow HSP-70 protein synthesis. Tubulin: loading control. **Bottom:** Relative HSP-70 protein expression (relative to wild-type heat-shocked levels, because control animals do not express detectable HSP-70 protein). Bars: mean value. Error bars: standard error. n = 3 biologically independent experiments. Statistical analysis: two-tailed unpaired t-test. **h.** U1 RNA levels upon heat shock in WT animals (RT-qPCR: relative fold change). U1 RNA values normalized to *pmp-3* mRNA values and shown relative to NHS levels. Y-axis: Relative RNA levels. X-axis: U1 RNAs. Bars: mean value. Error bars: standard error. n = 3–4 biologically independent experiments. Statistical analysis: 2-way ANOVA. **i.** Gene length dependence of heat-shock upregulated and downregulated protein-coding genes (p.adj <0.05). Y-axis: Average gene lengths (kb). X-axis: Strain. Genome-wide gene lengths of all expressed genes depicted for comparison. Boxplots: median value depicted. Error bars: standard error. Statistical analysis: Tukey HSD. **j.** Elongating RNA polymerase II (anti-S2P) levels at *hsp* gene. **Top:** Schematic of *hsp-70 (C12C8.1)* gene, and regions probed: Transcription Start Site (TSS) and distal regions (3’) of *hsp-70* (*C12C8.1*). **Bottom:** ChIP-qPCR showing fold enrichment over input. Y-axis: % ChIP input. X-axis: Strain, and region assayed. Bars: mean value. Error bars: standard error. n = 4–8 biologically independent experiments. Statistical analysis: 2-way ANOVA.

Animals were fed 4-thiouridine (4sU) at non-inhibitory concentrations (Fig 3a; Suppl. Fig 7a, b), subjected to heat shock, exposed to UV (365 nm) to crosslink nascent 4sU-containing RNA to Pol II at the described time points, Pol II was immunoprecipitated, and the RNA species associated with Pol II identified using absolute and stem-loop RT-qPCR. In wild-type animals, as expected, unspliced *hsp* pre-mRNAs co-immunoprecipitated with Pol II at 30 minutes after heat shock, decreasing by 2 hours post-heat shock recovery (Fig 3b; Suppl. Fig 7d). Nevertheless, a small but significant fraction of nascent pre-mRNA remained associated with Pol II 2 hours post-heat shock (Fig 3b; Suppl. Fig 7d), when Pol II was no longer transcriptionally engaged (ChIP-qPCR; Suppl. Fig 5e, f). Notably, 22G-RNAs antisense to *hsp-70* (*C12C8.1*) also co-immunoprecipitated with Pol II, 2 hours post-heat shock (Fig 3c), as concomitantly, increasing amounts of *hsp-70* mRNA were spliced, and also associated with Pol II (Fig 3d). Furthermore, chromatin fractionation showed that these progressively increasing spliced mRNAs were retained in chromatin (Fig 3e, f). In *prde-1* mutants, less pre-mRNA (Fig 3b), but more constitutively spliced nascent mRNA (Fig 3d) was associated with Pol II at 30 minutes heat shock, and 22G-RNAs were not present (Fig 3c). In addition, spliced *hsp-70* mRNA failed to increase on chromatin (Fig 3e, f), or in association with Pol II above these basal constitutive levels (Fig 3d), instead appearing to be efficiently exported and translated as seen by elevated HSP-70 protein levels (Fig 3g).

A broadly similar dynamic was observed with *hsp-70 (F44E5.4/.5)*, even though the majority of RNA was spliced co-transcriptionally (Suppl. Fig 5k). Here, by the end of 30 minutes of heat shock, the remaining amounts of pre-mRNA that associated with Pol II were similar in wild-type and *prde-1* animals (Suppl. Fig 7d), but significantly more mRNA was crosslinked with Pol II in wild-type animals (Suppl. Fig 7e), remained associated with Pol II during recovery (Suppl. Fig 7e), and accumulated in chromatin (Suppl. Fig 7f). Also, like with *hsp-70 (C12C8.1)*, significantly more 22G-RNA associated with Pol II in wild-type animals compared to *prde-1* (Suppl. Fig 7g). Perhaps due to the difference in splicing strategy between the two *hsp-70s*, 22G-RNA kinetics in association with Pol II varied: at 30 minutes upon heat shock, 22G-RNA associated with Pol II decreased in *prde-1*, rather than increasing in wild-type. Notwithstanding these differences, these data together showed that the piRNA-generated 22G-RNAs act at the transcription complex, associating with Pol II, to delay splicing, and promote chromatin retention of *hsp* mRNAs, postponing the availability of *hsp-70* mRNAs for translation. The continued association of unspliced and spliced RNAs along with 22G-RNAs with Pol II after transcription was complete and Pol II had disengaged from the *hsp* genes — most evident in *hsp-70 (C12C8.1)*, where a larger fraction underwent post-transcriptional splicing — also suggested that post-transcriptional splicing of *hsp* RNA occurs in direct or indirect association with Pol II.

The ability of antisense 22G-RNAs to retard splicing at the transcription complex through association with Pol II and nascent pre-mRNA was reminiscent of mechanisms by which U1 snRNA base-pairs with nascent transcripts to modulate their splicing efficiency and rates, and initiates telescripting, a process that prevents premature transcription termination, particularly of long-intron containing genes(50–52). In mammalian cells, heat shock decreases U1 snRNA levels, resulting in the loss of telescripting and a decrease in the expression of long-intron-containing genes(53, 54). Like mammalian cells, *C. elegans*, also showed a modest but significant decrease in at least three of five U1 snRNAs tested (Fig 3h; Suppl. Fig 7h). Yet, unlike mammalian cells, in wild-type *C. elegans,* despite reduced U1 RNA levels, genes upregulated upon heat shock were preferentially enriched for long-intron-containing genes. This was visible in the increase in their average gene lengths (Fig 3i) and the cumulative distribution plots of gene lengths of differentially upregulated and downregulated genes (p.adj <0.05) (Suppl. Fig 8a), and metagene profiles of expression values of all expressed genes segregated by gene length (Suppl. Fig 8b, c; Suppl. Table 9). *prde-1* animals showed a marked deficiency in upregulating long genes, although they did not differ from wild-type in downregulation of long genes (Fig. 3i, Suppl. Fig 8a; also see Suppl. Fig 8b, c). This raised the intriguing possibility that the 22G-RNAs generated by wild-type animals were functionally substituting for U1 snRNAs during stress, ensuring productive Pol II elongation and expression of long-intron-containing genes. In support of this, ChIP–qPCR showed that elongating Pol II (S2P) levels were markedly and significantly decreased at *hsps (C12C8.1* and *F44E5.4/.5)* 3′ ends in *prde-1*, despite normal occupancy at the 5′-end, mirroring phenotypes caused by disrupting U1–nascent RNA interactions(51, 52) (Fig 3j; Suppl. Fig 8d).

Together, these findings indicate that the piRNA pathway delays splicing and promotes chromatin retention of mature mRNAs by associating with the transcription complex. In doing so, the antisense 22G-RNAs act in a manner strikingly similar to U1 during telescripting: both systems protect splicing, preserve Pol II elongation, and the expression of long-intron containing genes, essential for development.

### The piRNA pathway preserves transcript fidelity and complements nonsense-mediated decay as a mechanism enforcing RNA quality control.

**Figure 4.**
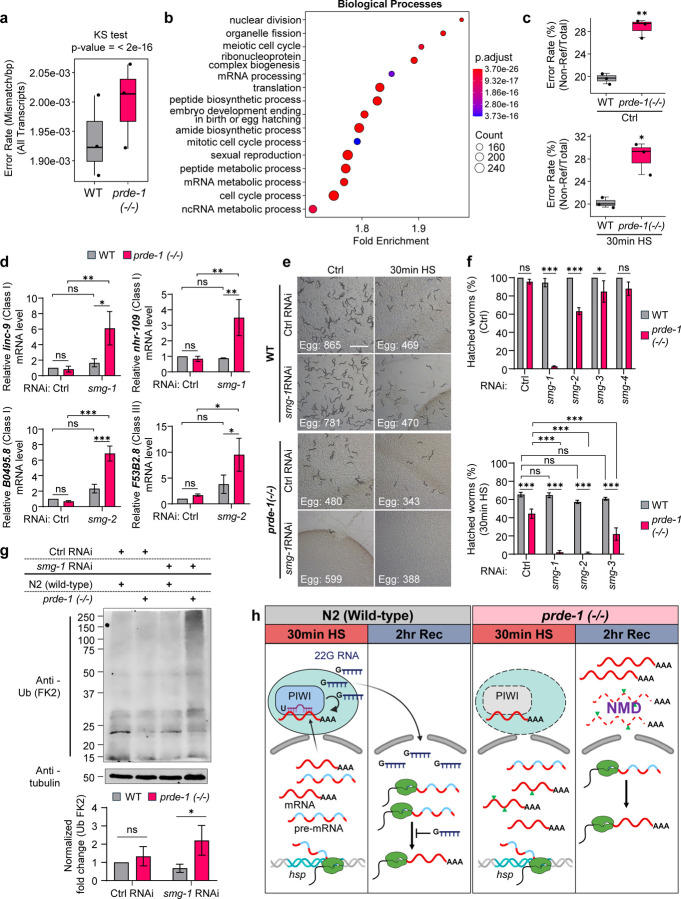
piRNA protects transcript fidelity and complements nonsense-mediated decay (NMD) to maintain protein quality control. **a.** Mean error rate per nucleotide for all transcripts (generated from ‘pileup’) in wild type and *prde-1* mutants. Y-axis: Mean error rate (mismatches per bp). X-axis: strain. Approximately 3.71 × 10^7^ individual mRNAs analyzed per biological replicate per strain. See **Methods** for details. Boxplots: median value depicted. Error bars: standard error. Statistical analysis: Kolmogorov-Smirnov (KS) test **b.** Gene Ontology analysis for transcripts with higher error rate in *prde-1* animals versus wild type. **c.** Variant error rates in differentially expressed genes (p.adj <0.05) between wild type and *prde-1* mutants (variants identified by GATK Haplotype Caller and SnpEff). **Top:** Control conditions. **Bottom:** Heat shock conditions. Y-axis: % variants. X-axis: Strain. Boxplots: median value depicted. Error bars: standard error. Statistical analysis: two-tailed unpaired t-test. **d.** Hyperactivation of NMD in *prde-1* animals. NMD-related gene expression levels in WT and *prde-1* animals on Ctrl (L4440) and *smg-1* or *smg-2* RNAi (RT-qPCR: relative fold change). 3 Class I NMD targets (*linc-9*, *nhr-109*, *B0495.8*) and 1 Class III NMD target (*F53B2.8*) expression values normalized to *eft-3* mRNA values and shown relative to wild-type Control RNAi-treated animals. Y-axis: Relative RNA levels. X-axis: RNAi-treatment. Bars: mean value. Error bars: standard error. n = 4–6 biologically independent experiments. Statistical analysis: 2-way ANOVA. **e-f.** NMD dependence of *prde-1* animals. **(e)** Representative DIC micrographs of progeny from control and heat-shocked WT and *prde-1* animals on Ctrl (L4440) and *smg-1* RNAi. The number of eggs laid by the mothers in one representative experiment is shown. Note: WT progeny have grown into adults. Scale bar: 2mm. **(f)** Percent viable embryos from mothers subjected to RNAi-mediated downregulation of NMD components under **(Top)** control, and **(Bottom)** heat shock conditions. Y-axis: Percentage of viable embryos. X-axis: RNAi-treatment. Bars: mean value. Error bars: standard error. n = 3–5 biologically independent experiments. Statistical analysis: 2 way-ANOVA. **g.** Polyubiquitinated protein levels in WT and *prde-*1animals exposed to control (L4440) or *smg-1* RNAi. **Top:** Representative western blot (antibody:clone FK2). Tubulin: loading control. **Bottom:** Quantification of polyubiquitinated protein (normalized to wild-type Control RNAi-treated animals). Bars: mean value. Error bars: standard error. n = 5 biologically independent experiments. Statistical analysis: 2 way-ANOVA. **h.** Model for the mechanism through which piRNA/22G-RNA pathway enforces RNA quality. In the presence of piRNA, splicing rates are restrained, thereby preserving RNA fidelity. In the absence of piRNAs, splicing is precocious, mRNAs accumulate errors, and NMD is hyperactivated to eliminate erroneous transcripts.

The accelerated splicing observed in *prde-1* mutants, even under heat shock conditions when spliceosome function is challenged, and reduced RNA polymerase II occupancy at the 3′ ends of *hsp* genes (Fig 3j, Suppl. Fig 8d), suggested to us that in the absence of piRNAs, mRNA transcripts may be accumulating errors due to aberrant splicing or premature termination. To directly test this hypothesis, we used Unique Molecular Identifiers (UMIs)(26–30) to label individual mRNAs from heat-shocked wild-type and *prde-1* mutants during first-strand cDNA synthesis, prior to PCR amplification and library preparation, and subjected these UMI-labeled mRNAs to RNA-sequencing. Following sequencing, we derived consensus mRNA sequences from grouped UMI-labeled mRNAs, which allowed us to differentiate random PCR-generated errors from errors within the original mRNA molecule, mapped these sequences onto the *C. elegans* reference genome, excluded values that could be attributed to potential germline SNPs (see Methods for details), and identified regions of mismatch, reflecting transcript variants or ‘errors’ (Suppl. Fig 9a; Suppl. Online Material). We then quantified the error rate in the consensus RNA molecules, from protein coding genes, or other genomic regions in wild-type and *prde-1* mutants, using two methods: (i) we generated a ‘pileup’ of all consensus sequences aligned to the reference genome and identified the percentage of nucleotides at each genomic position which were mismatched, and (ii) as a more stringent method, we identified high quality variant containing sites using the program HaplotypeCaller in the Genome Analysis Toolkit (GATK), and selected those annotated by SnpEff with a predicted impact of “High”, “Moderate” or “Low” as potential ‘errors’. To increase our confidence that any mismatches were ‘errors’, in both methods, we further selected regions with a minimum coverage of > 10 reads (Suppl. Fig 9a; Suppl. Online Material). Generating a pileup from the aligned mRNAs allowed us to approximate a mean error rate across all nucleotides (Global error rate) during heat shock as being in the order of approximately 10–3/bp, and compare the distribution of error-prone mRNA sequences between *prde-1* and wild-type. This differed significantly (Kolmogorov-Smirnov (KS) test; p<2e −16; Suppl. Table 10), with mRNA from the *prde-1* animals displaying a higher mean error rate than wild-type (Fig 4a) and expressing error-prone mRNAs enriched for distinct biological processes (Fig 4b; Suppl. Table 11, 12). *prde-1* mutants also displayed an increased error rate when variants were called more stringently using HaplotypeCaller (Fig 4c, Suppl. Fig 9b, c; Suppl. Table 13). Here, 1315–1684 nucleotide positions in the approximately 3.7 × 10^7^ individual mRNAs per sample analyzed were identified as high-quality variant-sites, and a significantly larger fraction of mRNA molecules that were differentially upregulated in *prde-1* mutants compared to wild-type, under either control or heat shock conditions, harbored these variants (Fig 4c; p.adj <0.05; Suppl. Table 13). Notably, the *prde-1* variant mRNAs were not uniformly distributed across the genome; instead, they were disproportionately enriched in mRNAs from smaller genes, which were also more highly expressed by *prde-1* (Suppl. Fig 9c; see Suppl. Fig 8c for expression levels). Amongst the base changes G>A was the most highly represented (Suppl. Fig 9d), consistent with the increased error rates associated with misincorporation of faster-incorporating bases by Pol II(55). This suggested that the splicing delay imposed by the piRNA pathway protected transcript fidelity under stress, and in its absence, animals may be accumulating more ‘errors’ in their transcripts. However, this analysis did not exclude the possibility that these variants represented RNA modifications or increased RNA-editing in the *prde-1* animals.

Therefore, to independently evaluate whether *prde-1* animals accumulated more splicing errors, we examined whether these animals have upregulated the nonsense-mediated mRNA decay (NMD) pathway, a system that surveils and eliminates transcripts that are unproductively spliced(56–61). If this were the case, we expected known mRNAs that are constitutive NMD targets to be expressed at higher levels upon downregulating *smg-1*, the NMD kinase, or *smg-2* (homolog of UPF1)(62). Amongst five previously characterized, reliable NMD targets in *C. elegans* (61), four were upregulated more in *prde-1* than in wild type animals upon downregulating *smg-1* or *smg-2* (Fig 4d). This implied that in the absence of piRNA, *prde-1* mutants may be constantly generating unproductively spliced mRNAs, that activated NMD.

This hypothesis was strongly supported: downregulating *smg-1*, resulted in the complete loss of viability of *prde-1* embryos, not only upon heat shock but also under control conditions (Fig 4e, f). Downregulating *smg-2* and *smg-3* (homolog of UPF2)(62) in parents also resulted in complete or partial loss of viability in their embryos, again even under control conditions, or if parents were subjected to heat shock (Fig 4f). In contrast, wild-type embryos were not affected by *smg-1, smg-2, smg-3* RNAi, under either heat shock or control conditions (Fig 4e, f), consistent with previous studies showing that NMD is not required for *C. elegans* survival (56, 62, 63). Thus, it appeared that the mis-transcribed mRNA in *prde-1* was likely being eliminated during translation to maintain viability. Consistent with this expectation, in the absence of *smg-1*, *prde-1* animals accumulated significantly higher amounts of polyubiquitinated proteins even under control conditions, in the absence of proteotoxic stress (Fig 4g; see methods for how viable *smg-1*-RNAi-treated *prde-1* were obtained).

Taken together, these studies suggest that piRNAs play a critical role in maintaining RNA integrity, acting as a complementary mode of RNA quality control to NMD: piRNAs delay splicing to preserve accurate pre-mRNAs under stress, while NMD serves to eliminate erroneous transcripts when this buffering mechanism is lost (Fig. 4h).

## Discussion.

Post-transcriptional control of gene expression is emerging as a critical mechanism to regulate development, stress, and phenotypic plasticity(47, 48). Yet how cells select transcripts for delayed splicing, how such delays are mechanistically implemented, and whether and how splicing decisions are coordinated across diverse tissues remain poorly understood. The sequence complementarity of 22G-RNAs to expressed exonic regions of mRNAs, their responsiveness to transcriptional and translational events within the cell, the interactions of 22G-RNAs with nascent transcript and Pol II after Pol II has dissociated from its target gene, and the ability of small RNAs to cross cell boundaries might allow the piRNA/22G-RNAs to act as flexible and systemic modulators of post-transcriptional splicing decisions. While we are yet to identify the detailed mechanisms of splicing regulation, the similarities to U1 RNA-mediated splicing and telescripting(50–54) are striking: both systems use base-pairing of noncoding RNAs to stabilize nascent transcripts, bias splicing, stabilize pre-mRNAs, prevent the premature loss of elongating Pol II from the 3’ end of genes, and protect the expression of long-intron-containing genes. These parallels raise the intriguing possibility that, analogous to therapeutic antisense oligonucleotides under clinical investigation, *C. elegans* may employ piRNA/22G RNAs as a natural small RNA–guided telescripting system to substitute for, or complement, U1 RNAs in RNA splicing.

Only very few previous studies have linked the piRNA pathway to splicing regulation(64, 65). Yet, these studies suggest that the piRNA-splicing-regulation may be conserved across species. Thus, in *Drosophila*(64) nuclear Piwi complexes inhibit splicing of the *gypsy* retrotransposon through chromatin-based, co-transcriptional silencing, whereas in locusts(65) piRNAs facilitate splicing of specific pre-mRNAs during oocyte maturation, suggesting both inhibitory and stimulating role for piRNAs in splicing control. Intriguingly, in *C. elegans*, the presence of introns protects transcripts from default Argonaute-mediated silencing in the germline, and the WAGO Argonaute, HRDE-1 have been shown to interact with the conserved RNA helicase Aquarius/EMB-4(66–68), further implicating piRNA function in RNA splicing regulation.

What might be the rationale for this unexpected role of piRNAs in splicing observed in our studies? piRNAs were initially discovered in germline cells, where they suppress transposons in many animal species. In this context, it is intriguing to note that spliceosomal introns themselves are considered to have evolved from Type II transposons(69, 70), opening the tantalizing possibility that the regulation of splicing a possible ancient, and vestigial function of piRNAs. Still, across species, piRNA and their associated PIWI proteins play broad roles in regulating gene expression in both somatic and germline cells, distinguishing self-RNAs from non-self-RNAs like transgenes and retroviral sequences, directing these ‘non-self’ transcripts for post-transcriptional gene silencing and degradation, directing epigenetic programming, DNA rearrangements, mRNA turnover and translational control (4, 6, 8, 10, 34, 35, 71–80). Our studies suggest that the role of piRNAs in regulating splicing and protecting transcript fidelity, while also interacting with the NMD machinery, may be central to these different roles, allowing the piRNA pathway to determine which newly synthesized transcripts are permitted sustained expression. piRNAs are amongst the fastest evolving small RNAs (4, 6). Likewise, AS is among the most divergent modes of gene expression and has been described as a form of ‘evolutionary tinkering’(44, 81–84). Indeed, it is thought that AS most likely evolved as an inevitable consequence of splicing errors and was later selected to result in the structural and functional diversification of proteins(44, 81–84).

In *C. elegans*, loss of the piRNA pathway leads to a sporadic, and temperature-dependent increase in infertility over time, a defect that can be mitigated by slowing growth or metabolism(9, 85). In addition, NMD, which mainly silences pseudogenes, is crucial for longevity(86, 87). Our findings suggest that similar to aging-dependent neurodegenerative disorders, the increased infertility upon piRNA loss may be due to the progressive accumulation of RNA errors arising from the loss of piRNA-control over splicing rates, and increased misfolded proteins due to an overwhelmed NMD system. Taken together with the known roles of piRNAs in transposon and pseudogene regulation of germline mRNAs, and the growing evidence for links between splicing, genome stability, and transposon activation(88), our findings suggest that piRNAs pathway may serve as a conserved node to integrate genome surveillance with RNA and protein quality control.

## C. ELEGANS STRAINS AND GROWTH CONDITIONS

*C. elegans* used in all experiments are listed in Method Table 1. Strains were obtained from the *Caenorhabditis Genetics Center* (CGC, Twin Cities, MN), generated in the Prahlad laboratory, or generated by SunyBiotech (Suzhou, Jiangsu, China). All crossed worms were verified by genotyping. The primers used in genotyping are listed in Method Table 2.

All strains were maintained at 20°C on nematode growth media (NGM) plates. NGM plates were prepared by pouring 8.9 ml of autoclaved liquid NGM per 60 mm plate. NGM was allowed to solidify at room temperature (RT) for 2 days, seeded with 300 μl OP50 *E. coli* (O.D = 1.8–2.0), and dried at RT for no more than 2 days. Population densities were matched based on growth rate of the strains. To maintain healthy continuously growing populations, 10–15 animals at larvae stage 4 (L4) were passaged onto a new plate every 4 days. For experiments, day-one adult animals were used. These were generated either by picking L4-stage worms and harvesting them 24–24 hrs later, or by bleach-hatching and collecting day-one adult worms 82–84 hrs later, depending on experimental requirements.

## HEAT SHOCK

Worms were exposed to acute heat stress as previously described in Das et al., elife 2020^1^. Briefly, NGM plates containing day-one adults were sealed with parafilm and immersed in a water bath pre-warmed to 34°C for different durations as mentioned. For recovery experiments, NGM plates were incubated in a 20°C incubator immediately after heat shock and harvested after specific times mentioned in the experiments.

## RNA SEQUENCING

### RNA Library Preparation

For RNA-sequencing, three independent biological replicates of each strain and each treatment were harvested and processed as described. For each sample/treatment, approximately 200 L4-stage worms were transferred to OP50 plates. After 26–27hrs, worms from two plates per condition [non-heat shock (NHS), 5min HS, 30min HS, and 30min HS + 2hr Rec] were collected in nuclease-free water. Total RNA was extracted using the Mirvana miRNA Isolation Kit (Invitrogen; Cat #AM1560). Genomic DNA in the sample was removed using Turbo DNA-free Kit (Invitrogen; Cat #AM1907). RNA was separated by size into mRNA (>200 nt) and small RNA (<200 nt). Small RNA samples were treated with 10U RppH (NEB; Cat #M0356S) at 37°C for 30 min, then purified using the Zymo RNA Clean & Concentrator kit (Zymo Research; Cat #R1015). RNA concentrations were measured with a Qubit 4 fluorometer (Thermo Scientific). mRNA libraries were prepared using the KAPA mRNA Hyperprep kit (Roche; Cat #08098123702); small RNA libraries were prepared using the Illumina TruSeq Small RNA Library Preparation kit (Illumina; Cat #RS-200), following manufacturer’s protocol.

### Small RNA analysis

Small RNA data was analyzed using the tinyRNA^2^ (v1.50) pipeline with its default configuration. The process included adapter trimming and quality filtering with fastp^3^, followed by genome alignment to the *C. elegans* genome (release WS279) using Bowtie^4^. An additional setting, counter_decollapse: True, was specified to generate decollapsed SAM files. The module tiny-count was used to count the different types of small RNA according to the selection rules specified in the following table.

**Table:** 

Select for...	with value...	Classify as...	Hierarchy	Overlap	Strand	5’End Nucleotide	Length
Class	risiRNA	rRNA	1	Partial	both	all	all
Class	miRNA	miRNA	2	5’Anchored, 0,4	sense	all	all
Class	piRNA	piRNA	2	5’Anchored	sense	all	18–21
Class	CSR	CSR Class 22G	3	Partial	antisense	G	21–23
Class	WAGO	WAGO Class 22G	3	Partial	antisense	G	21–23
Class	ALG	ALG Class 26G	3	Partial	antisense	G	26
Class	ERGO	ERGO Class 26G	3	Partial	antisense	G	26
Class	ALG	ALG target 22G	3	Partial	antisense	G	21–23
Class	ERGO	ERGO target 22G	3	Partial	antisense	G	21–23
Class	unk	unclassifed 22G	3	Partial	antisense	G	21–23
Class	unk	unclassifed siRNA	4	Partial	both	all	21–24

The read counts obtained from tiny-count were used to generate genome-wide coverage profiles for the 22G-RNAs and piRNA. The coverage was normalized as Read per million (RPM) and output as BigWig (bw) files using rtracklayer^5^. Differential Expression was computed by using a modified version of tyny.deseq.r, the R module was adjusted to remove the ribosomal RNA (rRNA) from the analysis, and to calculate the gene expression in RPM in addition to DESeq normalized counts. Changes in expression were presented as log2 fold-change, and genes with an adjusted p-value of <0.05 (after correction with Benjamini & Hochberg)^6^, were considered significant.

### mRNA seq analysis

RNA-seq samples were analyzed and processed using the nf-core/rnaseq (v3.12.0) pipeline, which was executed with Nextflow^7^ (v22.10.6). Sequence quality was assessed with FastQC ^8^(v0.11.9), and low-quality reads, and adapters were removed using Trimmomatic^9^ (v0.67). The processed reads were then aligned to the *C. elegans* genome (WBcel235, Ensembl release 111) with STAR^10^ (v2.7.9a) using default settings. Alignments were quantified against the WBcel235 annotation with Salmon (v1.9.0). Alignment quality control was performed with Qualimap^11^ (v2.2.2) and RSeQC^12^ (v3.0.1).

### Differential Expression

Differential expression analysis was performed between RNA-seq samples using **DESeq2** with the **Wald test**. Expression changes were presented as log2 fold-change. Genes with an adjusted p-value <0.05, following correction with the **Benjamini & Hochberg**^6^ method, were considered significantly differentially expressed.

### Analysis of Alternative Splicing Events

To identify differential alternative splicing events, RNA-seq data were re-processed using the nf-core/rnasplice (v1.0.4) workflow. This pipeline was executed with Nextflow (v23.10.0) and relied on reproducible software environments from Bioconda and Biocontainers.

Within the rnasplice pipeline two modules were used to detect differential splicing and calculate the Percent Spliced-In (PSI) for each event, SUPPA2^13^ (v2.3) and rMATS (v4.1.2)., Splicing

events were considered significant if the absolute difference in PSI (dPSI) between the 30 minutes heat shock and control conditions had a p-value <0.1.


$$\text{Formula}\;:\;\Psi \;=\sum \left(TP\;Minclusion\right)+\sum \left(TP\;Mexclusion\right)\sum {\left(TP\;Minclusion\right)}^{13}$$

Pearson’s Chi-squared test with Yates’ correction was used to determine if the number of up- or down-regulated splicing events during heat shock differed between the N2 and PRDE1 strains (p-value <0.05).

Differential Transcript Usage (DTU) analysis was performed using SUPPA2. First, the usage of transcripts (isoforms) was tested for significant differences between conditions. Isoforms with a p <0.1 were considered significant. For analysis and visualization, the average Percent Spliced In (PSI or Ψ) value for each isoform was calculated across the biological replicates within each condition. The resulting list of PSI per isoforms was then filtered to retain only those occurring in genes previously identified as differentially expressed (DEGs). Student t-test was used to compare the means between the conditions at significance level p <0.05.


Formula:PSI_i=TPM_i/∑_j=1^nTPM_j13

### Gene Ontology

Gene Ontology analysis was performed by using Over Representation Analysis (ORA), with the R package ClusterProfiler^14,15^. GO annotations for *C. elegans* were obtained from R package org.Ce.eg.db: Genome wide annotation for Worm^16^ (version 3.18.0). Ontology terms enriched with an adjusted p-value <0.05 or <0.1 (Benjamini & Hochberg)^6^ were considered significant. Fold-Enrichment was calculated as the ratio of the frequency of input genes annotated in a GO term to the frequency of all genes annotated to that term^14^.

### Data Visualization

The metaprofiles were generated using Deeptools^17^ (v3.56) by normalizing the coverage by RPM per bp) and then modified to plot them using R.

Venn diagrams were generated using the Eulerr^18^ package (v.7.0.2) in R^18^. Additional plots were generated using ggplot2 (v.3.5.1)^19^ .

## RNA FISH

### mRNA FISH

RNA FISH was performed as previously described^20^. Probes were designed against mRNA according to the Biosearch Technologies website, and consisted of 25–35 11bp sequences that were complementary to the *hsp-70s (C12C8.1* and *F44E5.4/.5)* mRNA. For mRNA FISH, about 20 day-one adult worms were harvested in 1x PBS and fixed in 4% paraformaldehyde (Electron Microscopy Science; Cat #50-980-487) for 45 mins. After two washes with 1x PBS, worms were permeabilized in 70% ethanol at 4°C for 24–26 hrs. Worms were washed with Stellaris Buffer A (Biosearch Technologies, Cat # SMF-WA1–60) and incubated at 37°C for 16 hrs in hybridization buffer (Cat #SMF-HB1–10) containing 0.2 mM FISH probes targeting *hsp-70s (C12C8.1* and *F44E5.4/.5)* mRNA. After washing three times with pre-warmed Wash Buffer A and one time with Wash Buffer B (Biosearch Technologies, Cat # SMF-WB1–20), worms were mounted in Vectashield with DAPI (Vector Laboratories; Cat #H-1200–10). Imaging was performed using a Leica TCS SPE Confocal Microscope with 20x dry and 63x oil objectives, and LAS AF software.

### 22G RNA FISH

For 22G-RNA FISH, animals were processed as described above for mRNA FISH. Fluorescence conjugated probes targeting 22G-RNAs were purchased from IDT, and were complementary to one of the most abundant 22G-RNAs identified by sRNA-seq (hence they were in the sense direction as mRNA, and could not hybridize to mRNA). 0.2 μM FISH probe targeting *F44E5.4/.5* 22G RNA was used per slide. The sequence of the probe is in Method Table 3.

## AUXIN-INDUCIBLE DEGRADATION (AID) TREATMENT

Auxin plates were prepared at a final concentration of 4 mM by adding indole-3-acetic acid (Alfa Aesar; Cat #A10556), dissolved in ethanol at 400 mM, to autoclaved NGM that had cooled to approximately 55°C prior to pouring. NGM plates with ethanol served as controls. Plates were dried at RT for 2 days, then for 2 more days after seeding with 300 μl OP50 (O.D = 1.8–2.0). About 20 day-one adults were transferred to OP50-seeded auxin or ethanol plates and incubated at 20°C for 4 hrs. Worms were then either not heat shocked (NHS) or subjected to heat shock (HS) for 30mins at 34⁰C and processed for FISH as above.

## IMMUNOSTAINING

Immunostaining of dissected gonad was performed as previously described^20^ . 10–15 day-one adults were picked into 15 μl 1x PBS on a cover glass (Leica Biosystem; Cat #3800105) and dissected with a surgical blade #11. Dissected worms were fixed in 4% paraformaldehyde for 6 mins. A charged slide (Epredia; Cat #4951PLUS-001) was placed over the cover glass, then quickly transferred onto a metal block that had been cooled with dry ice for over 10mins. After quickly removing the cover glass, samples were post-fixed in 100% methanol pre-chilled at −20°C for 2mins, washed in 1x PBST (1x PBS with 0.1% Tween-20), and then blocked in 1x PBST with 0.5% BSA for 1 hr. Samples were incubated at 4°C overnight with Mouse anti-FLAG M2 (Sigma-Aldrich, Cat #F3165–1MG), diluted 1:2,000 in 1x PBST. Following washing, samples were incubated at RT for 2 hrs with Donkey anti-Mouse Alexa Fluor 488 (Invitrogen; Cat #A32766), diluted 1:500 in 1x PBST. After washing, samples were mounted in the Vectashield with DAPI and imaged.

## RNA INTERFERENCE (RNAi)

RNAi experiments were conducted using the standard feeding RNAi methods. Bacterial clones expressing the control (empty vector pL4440) construct and the dsRNA targeting different *C. elegans* genes were obtained from the Ahringer RNAi library^21^ , now available through Source Bioscience. All RNAi clones used in experiments were sequenced for verification. For RNAi experiments, RNAi bacteria with empty (pL4440 vector as control) or the dsRNA-expressing plasmid were grown overnight in LB liquid culture containing ampicillin (100 μg/ml) and then induced with IPTG (1 mM) for 2 hrs before seeding the bacteria on NGM plates supplemented with ampicillin (100 μg/ml) and IPTG (1 mM). Bacterial lawns were allowed to grow 48hr before the start of the experiment. RNAi-seeded plates were used for RT-qPCR experiments to measure NMD-related gene expression, to evaluate the hatch rate of embryos, and for immunoblot assays (to analyze ubiquitinylated proteins). For RT-qPCR and Western blot experiments, worms were initially grown on OP50 plates for 24 hours after egg laying or bleaching, respectively. Then, larvae were transferred to RNAi plates and grown for 60–62 hours before harvesting. This was done to bypass the embryonic lethality of *smg-1* or *smg-2* RNAi.

## RNA ISOLATION AND QUANTIFICATION OF RNA LEVELS BY RT-qPCR

RNA was extracted from 30-40-day-old adults exposed to the indicated heat shock durations and recovery times. Animals were collected in 50 μl Trizol and immediately snap-frozen in liquid nitrogen. Samples were thawed on ice, mixed with 200 μl Trizol, and lysed using Precellys 24 homogenizer. RNA was isolated from lysates with chloroform and precipitated with isopropanol and glycogen. After washing with 70% EtOH, RNA pellet was dissolved in nuclease-free water and treated with TURBO DNase. cDNA was synthesized using iScript cDNA synthesis kit (Bio-Rad; Cat #1708891). qPCR was performed using PowerUp SYBR Green Master Mix (Applied Biosystems; Cat #A25742) on a QuantStudio 3 Real-time PCR System (10 μl reaction, 96-well plate). The amounts of RNA were quantified using one of the following methods:

### mRNA RT-pPCR

#### (i) Relative quantification using the ΔΔCt method:

mRNA (exon-exon junction primers), and pre-mRNA (intron-exon primers) levels were computed relative to *pmp-3* (previously verified by use to remain steady during heat shock)^1^, which served as an internal control. For NMD-related gene expression analysis, *eft-3* was used as internal control, as previously described^22^. Primer locations are depicted in the Method Fig 1 below and primer sequences are in Method Table 2.

#### (ii) Absolute quantification using standard curves:

Absolute amounts of pre-mRNA and mRNA were estimated through the generation of standard curves using serial dilutions of defined concentrations of *hsp-70 (C12C8.1)* and *F44E5.4/.5* mRNA, pre-mRNA, as well as *pmp-3* mRNA. The mRNA and pre-mRNA used to generate each standard curve, was obtained by PCR amplification from cDNA or genomic DNA extracted from whole worms, PCR amplified using primers external to the primer target sites used in the experiments, gel purification, and quantification using a Qubit 4 fluorometer. qPCR was conducted using 10-fold serial dilutions of a known concentration of purified PCR products. The standard curves were created from the CT values obtained from the dilutions (see Method Fig 1).

The amounts of mRNA and pre-mRNA were extrapolated derived from these curves using the formula.


$$\text{Formula}:\;Absolute\;amount\;of\;RNA\;=\;inital\;Con.\times \;10^{\left(\frac{CT\;Value\;\;-\;constant}{slope}\right)}$$

To account for differences in lysis efficiency, variations in harvesting of animals, and other factors, a similar curve was calculated for *pmp-3* and the final values were normalized to *pmp-*3 values known to remain stable through heat shock^1^.

The primers used for qPCR analysis were designed using Primer 3 software and generated by Integrated DNA Technologies (See Method Table 2).

### SMALL RNA (sRNA) RT-qPCR

Small RNA qPCR was performed using stem-loop RT-qPCR by modifying published protocols^23^ Total RNA extracted from animals, or immunoprecipitated after 4sU treatment and RNAPII immunoprecipitation (see below), incubated with 50 nM 22G RNA-specific stem loop primer at 70°C for 5mins, cooled on ice for ≥5 mins. cDNA was synthesized using 100U Superscript II Reverse Transcriptase (Invitrogen; Cat #18064014) in 1x reaction buffer, 1U SUPERase-In RNase inhibitor, and 0.5 mM dNTPs. The RT-qPCR mix included 2 μl cDNA, 0.25 μM 22G RNA-specific forward primer, 0.25 μM universal reverse primer,1x PowerUp SYBR Green Master Mix. Relative fold change of 22G-RNA was quantified relative to immunoprecipitated *pmp-3* using the ΔΔCt method. Primer specificity was tested using a synthetic sRNA (21ur-2348). The specificity of the reaction was verified by running the reaction on agarose gels. Representative gel and PCR design are shown in Suppl. Fig 7c. The primer sequences are listed in Method Table 3.

#### ChIP-qPCR

Chromatin immunoprecipitation (ChIP) was performed as previously described^20^ . Following exposure to one of three conditions (NHS, 30min HS, or 30min HS + 2hr Rec), approximately 700 day-one adults, synchronized by bleach hatching, were harvested in 1x PBS and crosslinked with 2% formaldehyde at RT for 10 mins. Crosslinking was quenched with 250 mM Tris (pH 7.4) for 10 mins. Samples were washed three times in ice-cold 1× PBS containing protease inhibitor cocktail (PIC; Thermo Scientific; Cat #78429) and then snap-frozen in liquid nitrogen. Pellets were resuspended in 200 μl FA buffer (50 mM HEPES/KOH (pH 7.4), 150 mM NaCl, 50 mM EDTA, 1% Triton X-100, 0.1% sodium deoxycholate, 0.5% sarkosyl) freshly supplemented with 1 mM DTT and 1x PIC. Worms were lysed using a Precellys 24 homogenizer and sonicated (Bioruptor Pico; 8–10 cycles of 30sec on/off). Debris was removed by centrifugation (13,500 rpm at 4°C for 5 mins). Protein A/G Magnetic Beads (20 μl per sample; Thermo Scientific, Cat #88803) were washed two times with FA buffer. To eliminate non-specific binding, 5 μl of beads were then added to the lysates and incubated at 4°C for 1 hr. 10% of the pre-cleared lysate was kept as input, and the rest was incubated overnight at 4°C with anti-total RNAPII (Diagenode; Cat #C1520004) or RNAPII s2P (Diagenode; Cat #15200005), followed by 3 hrs incubation with 15 μl beads. Beads were washed sequentially with low salt, high salt, LiCl, and TE buffers, then eluted in 150 μl elution buffer (10 mM EDTA, 1% SDS, 0.1 M sodium bicarbonate). Elutes and volume-matched inputs were de-crosslinked overnight with 0.8U proteinase K (NEB; Cat #P8107S) and 160 mM NaCl. DNA was purified using phenol/chloroform/isoamyl alcohol. qPCR was performed as above. All experiments were expressed as % input value. The sequence of the primers used for ChIP experiments are listed in Method Table 2 and depicted in Fig 3j; Suppl. Fig 5e, f, and 8d.

## RNA IMMUNOPRECIPITATION (RIP) FOLLOWED BY RT-qPCR

RNA immunoprecipitation (RIP) was performed with ~700 day-one adults per condition, expressing FLAG::FRG-1 (endogenous PRG-1 tagged with FLAG epitope using CRISPR/Cas9) synchronized by bleach hatching. To account for the non-specific interactions of the abundant *hsp* mRNA generated by heat shock, we used wild-type (N2) animals not expressing FLAG as a negative control, and compared specific immunoprecipitation (IP) of PRG-1 IP to this non-specific IP. RIP was conducted in the absence of cross-linking. Worms were harvested in nuclease-free water, pelleted, and snap-frozen in liquid nitrogen. Worms were resuspended in FA buffer with 1 mM DTT, 1x PIC, and 0.01U/μl SUPERase-In RNase inhibitor (Invitrogen; Cat #AM2696), then lysed and sonicated (2 cycles of 30 sec on/off). 10% of the pre-cleared lysate was kept as input, and the rest was incubated with anti-FLAG M2 (Sigma-Aldrich, Cat #F3165–1MG) at 4°C for 2–3 hrs, followed by beads for 1hr. After washing, beads were eluted with elution buffer containing 0.01U/μl SUPERase-In RNase inhibitor. Elutes and volume-matched inputs were de-crosslinked by incubating them with 0.8U proteinase K (NEB; Cat #P8107S) at 45°C for 1hr. RNA was isolated using Trizol and qRT-PCR was performed as above. Because we were had no reason to expect that *pmp-3* would also bind to PRG-1, and be immunoprecipitated, to control for variation in harvesting, lysis, loading etc., we normalized *hsps* in IP sample to *pmp-3* in the input sample using the ΔΔCt method. *bath-45*, known to interact with PRG-1 was used as a positive control for the efficacy of RIP. The sequence of the primers used for RIP experiments are listed in Method Table 2.

### 4sU RNAPII IP qPCR

All procedures for 4sU RNAPII immunoprecipitation (IP) are outlined in Fig 3a. Specifically, 4sU was delivered as a liquid suspension mixed with bacterial food. The bacterial food, OP50, was heat-killed to minimize its metabolism of 4-Thiouridine (4sU). To most optimally deliver 4sU within a short time, about 700 day-one adult worms were rapidly collected in nuclease-free water and then resuspended in S-basal containing 10mg/ml heat-killed OP50 and 4mM 4sU (Sigma Aldrich; Cat #T4509). The suspension was seeded onto empty NGM plates to prevent hypoxia (a critical step since *C.elegans* suspended in liquid media have a markedly different heat shock response). The worms, bacteria and 4sU were allowed to dry at RT for 1hr, and subjected to one of three conditions [not heat shocked (NHS), heat shocked at 34⁰C for 30 mins (30min HS), or heat shocked at 34⁰C for 30 mins and allowed to recover for 2 hrs at 20⁰C (30min HS + 2hr Rec)], and then crosslinked by exposure to UV (365 nm) for 30 sec. Samples were harvested in nuclease free water and snap-frozen in liquid nitrogen. Worm lysis, immunoprecipitation, RNA isolation, and qRT-PCR followed the RIP-qRT-PCR protocol, using anti-total RNAPII (Diagenode; Cat #C1520004) instead of anti-FLAG. To quantify mRNA and pre-mRNA, absolute amounts of RNA were estimated from standard curves and normalized by immunoprecipitated *pmp-3*.

## EU LABELLING OF NASCENT RNA

To examine the expression patterns of nascent *hsp* genes we used a pulse-chase approach using the Click-iT Nascent RNA Capture Kit (Invitrogen; Cat #C10365) according to the manufacturer’s instructions, to label RNA. In brief, 50 day-one adult worms were placed on an OP50 bacterial lawn containing 4 mM EU and incubated at 20°C for 10 mins to preload them with EU. Subsequently, the worms were incubated at 20°C (serving as controls) or underwent heat shock for 30 mins at 34⁰C. Control and heat shocked animals were harvested in 50 µl Trizol and immediately snap-frozen in liquid nitrogen. To assess maturation of pre-mRNA to mRNA, animals were allowed to recover in the absence of EU. For this, worms were transferred, immediately after heat shock, from EU containing OP50 plates onto new NGM plates seeded with regular OP50. The plates were then incubated at 20°C for 2, 4, or 6 hours, and subsequently harvested in Trizol. RNA isolation was performed as described in the RT-qPCR protocol. EU-labeled RNA was biotinylated with 0.4 mM biotin azide and purified by reprecipitating. The biotinylated RNA was captured using 12 µl of streptavidin beads. cDNA synthesis was carried out directly on the beads containing nascent RNA using the iScript cDNA synthesis kit. qPCR was processed as described in the RT-qPCR protocol. To quantify mRNA and pre-mRNA, absolute amounts of RNA were estimated from standard curves. To assess maturation from pre-mRNA to mRNA, mRNA expression level at each time point was divided by pre-mRNA expression at 30min HS. In both analysis, mRNA and pre-mRNA expression amounts were quantified relative to EU labeled *pmp-3* amounts.

## SUBCELLULAR FRACTIONATION

Subcellular fractionation was performed with ~700 day-one adults per condition synchronized through bleach hatching method. Worms were harvested in 1.5 ml tube with nuclease free water, pelleted and snap-frozen in liquid nitrogen. Frozen pellet was washed with 500 μl hypotonic buffer (15 mM HEPES/KOH (pH7.5), 10 mM KCl, 5 mM MgCl2, 0.1 mM EDTA, 350 mM Sucrose). Worms were resuspended in 100 μl hypotonic buffer supplemented with 1 mM DTT, 1x PIC and 0.01U/μl SUPERase-In RNase inhibitor and homogenized by pestle (SP Bel-Art ; Cat #F19923–0001). To remove worms’ bodies and debris, homogenates were centrifuged at 500 xg at 4°C for 5 mins, and supernatant was transferred to a new 1.5 ml tube. The supernatant was then centrifuged at 4,000 xg at 4°C for 5 mins to collect nucleic (pellet) fraction. The nuclei pellet was washed three times with 200 μl hypotonic buffer. After last washing, the pellet was resuspended in 120 μl nucleus lysis buffer (50 mM Tris (pH7.5), 140 mM NaCl, 1.5 mM MgCl2, 0.5% (w/v) NP-40) supplemented with 1 mM DTT, 1x PIC and 0.01U/μl SUPERase-In RNase inhibitor, and incubated in ice for 20 mins. 20 μl of sample was aliquoted to a 1.5 ml tube labelled as “input”, and the remaining samples were centrifuged at 20,000 xg at 4°C for 10 mins. The supernatant was transferred to a 1.5 ml tube labelled as “nucleoplasm”. The pellet was resuspended in 100 μl high salt solution (10 mM Tris (pH7.5), 700 mM NaCl) supplemented with 1 mM DTT, 1x PIC and 0.01U/μl SUPERase-In RNase inhibitor, and incubated in ice for 20 mins, and centrifuged at 20,000 xg at 4°C for 5 mins. The supernatant was transferred to a 1.5 ml tube labelled as “Chromatin”. The RNA in each sample was isolated using Trizol, and *hsp* gene expression level was measured as described in the RT-qPCR protocol. The results of experiments were presented as percentage input values.

This method of extraction did result in the loss of some nuclei. Nevertheless, the nuclei obtained were ‘clean’ and not contaminated with cytoplasmic fractions, confirmed by conducting a Western blot and probing for tubulin.

## EMBRYO HATCH RATE

Embryo hatch rate was assessed using 2–5 day-one adult worms, which laid eggs for different durations following NHS or 30min HS, as described previously^20^ . After removing mothers, embryos were counted and incubated at 20°C. The number of live progenies were scored after 72 hrs. For RNAi experiment, mothers were prepared by transferring them to RNAi plates at L4-stage and allowed to lay eggs for 12 hrs after treatment.

## WESTERN BLOT

Worm lysates were prepared by boiling samples in Laemmli sample buffer (Bio-Rad; Cat #1610737) with β-mercaptoethanol. For ubiquitinylated protein analysis, about 150 worms were prepared as described in the RNAi protocol. The worms were collected, snap frozen, and lysed by boiling and grinding using a pestle. Lysates were separated on SDS-PAGE (HSP-70: 10%, Ubiquitinylated protein: 12%) and transferred onto nitrocellulose membrane (Bio-Rad; Cat #1620115). Membranes were blocked at RT for 1 hr in Odyssey Blocking Buffer (LI-COR; Cat #927–50000), incubated overnight at 4°C with primary antibodies, and then with IRdye goat anti-mouse IgG 800CW (1:10,000; LI-COR; Cat #926–32210) at RT for 2 hrs. Imaging was performed using the LI-COR Odyssey Infrared System and analyzed with Image Studio software.

To detect HSP-70 levels, mouse anti-FLAG M2 (1:1000; Sigma-Aldrich; Cat #F3165–1MG) was used to detect HSP-70::FLAG in strains where endogenous *hsp-70* (C12C8.1) was tagged with CRISPR/Cas9. Mouse anti-Ubiquitinylated proteins, clone FK2 (1:1000; Sigma-Aldrich; Cat #04–263), was used to detect ubiquitinylated proteins. For tubulin detection as a loading control, mouse anti-tubulin, clone AA4.3 (1:1000; DSHB; Cat #AB_579793) was used.

## SMALL RNA INJECTION AND RECOVERY ASSAY

Small RNA was prepared from total RNA extracted from

- Wild-type animals worms subjected to 30 min HS
- *prde-1* animals subjected to 30 min HS

Small RNAs were fractionated using the Mirvana miRNA Isolation Kit. The small RNA was then injected at a concentration of 50 ng/μl. Following injection, worms were recovered at 20°C for 2 hrs and subsequently maintained under non heat shock conditions (NHS) or subjected to 30 mins heat shock at 34°C. Worms were collected in 1x PBS after 6 hrs incubation at 20°C and used for *F44E5.4/.5* mRNA FISH experiment. For RT-qPCR analysis, worms were collected immediately in Trizol, and their RNA harvested as described above.

## ANALYSIS OF mRNA VARIANTS

To assess whether the loss of piRNAs led to increased transcript errors, we devised a method to identify sequences of individual mRNA molecules prior to the possible incorporation of errors due to PCR amplification. RNA extracted from 3 biological repeats of: wild type *C. elegans* at 30 minutes at 34⁰C, and *prde-1* at 30 minutes at 34⁰C were used. mRNAs were tagged with UMIs using the **SMART-Seq^®^ Total RNA Pico Input with UMIs (ZapR^™^ Mammalian)**. The UMIs were attached to mRNAs during the first strand-cDNA synthesis, prior to PCR amplification. Subsequently, mRNAs were PCR amplified with 3 cycles of amplification for the initial library PCR and 12 cycles of amplification for the enrichment PCR. Sequencing was performed on the Novaseq 6000; the number paired-end reads sequenced were in the range of 50 million-80million, with a read size of 100 bp.

Next, data was pre-processed using a modified version of the RNA with UMIs v1.0.16 pipeline^24^ and a modified version of the GATK Best Practice: RNA-seq Variant Calling Workflow using the tools from gatk ( v4.6.1.0) and Picard (v3.3.0).

The complete list of relevant tools, and the custom script used are available on github (https://github.com/Prahlad-Lab/umi_celegans_analysis_consensus ) DOI 10.5281/zenodo.17873233.

The fastq.gz files required to run this analysis pipeline are available through the European Nucleotide Archive (ENA) at EMBL-EBI under accession number PRJEB101605 (https://www.ebi.ac.uk/ena/browser/view/PRJEB101605).

FastQC ^8^ was used to check the quality of the FASTQ paired-end reads. The FASTQ files were converted to unmapped bam (uBAM) using gatk FastqToSam tool. Unique molecular identifiers (UMI) were extracted using fgbio ExtractUmifromBam. The UMI were extracted using the parameters indicated in the SMART-Seq^®^ Total RNA Pico Input with UMIs (ZapR^™^

Mammalian) kit, the SMART UMI Adapter from read2 was removed by trimming reads a total of 12 nucleotides: “8 nt UMIs + 3 nt UMI linker + 3 nt”. The quality of the uBAM file was measured again with FastQC^8^.

Reads were aligned to genome with STAR^25^ (v 2.7.11b) using the *Caenorhabditis elegans* genomic reference and annotation (ENSEMBL WBcel235 version 114)^26^. Per-Sample two pass-mapping was enabled using the option -twopassMode Basic. The main output was an unsorted BAM that included unmapped reads, --outSAMtype was set to “BAM Unsorted” and -- outSAMunmapped was set to “Within”.

### UMI Grouping and Consensus Generation

Following alignment, BAM files were sorted by coordinating with Picard SortSam tool, then merged with unaligned Bam file to get the UMI tags. **fgbio GroupReadsByUmi** was used to create subgroups (families) based on their position, UMI, and, the parameters --strategy Adjacency --edits 1 and --family-size-histogram were used to correct sequencing errors within UMIs themselves and output the family sizes. Around 70–80% of the reads in all the replicates were Family size =1, as shown in the plot (Method Table 4).


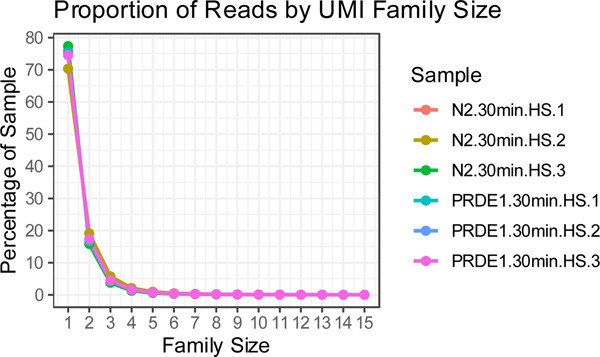


The different families were collapsed into single consensus reads by using fgbio CallMolecularConsensusReads. Families of size 1 were preserved using the parameter --min-reads 1. The consensus reads were mapped again to the WBcel235genome using STAR and merged again to unmapped BAM to get UMI tags.

Following the GATK best practices for RNA-seq Variants, BAM files were processed with gatk SplitNCigarReads to split the reads with “N” in their cigar. To filter out DNA variants, a recalibration model was built with gatk BaseRecalibrator. The resulting model was applied to the consensus BAM files with gatk ApplyBQSR to obtain the BSQR BAM.

### Pileup Generation and analysis

A pileup of the BSQR BAM was generated with bcftools mpileup using the parameter -d 1000000 to get keep the reads at all loci. **To increase confidence that we were detecting mRNA variants, and not stochastic nucleotide changes we:**

- analyzed only regions that had generated over 10 reads (i.e. at least 10 mRNAs corresponded to the region to identify the ones which were different). The pileup file was analyzed with a custom python script to filter the regions with total depth <=10 and count the proportion of alternative reads and reference reads.
- Excluded regions where “Alternative Read/Total Reads =1.0” or “0.5”. This was used to exclude possible homozygous or heterozygous SNPs with frequencies of 1 or 0.5, respectively, although this does not account for differences in expression from the different alleles.

A custom R script was used to generate tables with the statistics of the Total Number of “mistakes”, the Distribution of the Alternative Reads/Reference reads, a Kolmogorov-Smirnov Test was used to compare the distributions. GO analysis of the genes with more mismatches in PRDE1 than in N2 was performed.

### Variant Call and Analysis

HaplotypeCaller was used with the BSQR BAM to call variants and output VCF file. SnpEff ^28^ was used to annotate the vcf with the SnpEff wbcel235 internal annotation. A custom R script was used to read the VCF and filter the variants with total dept < =10, and SnpEFF Functional Impact type of “Modifier”. Variant Sites with a Ratio of Alternative Read/Total Reads =1.0were excluded to remove potential genomic Variants. The following were calculated:

- Transcription error rate: Ratio of Alternative Read/Total Reads
- Deleterious Variant Rates: Ratio Deleterious Variants/Total Reads
- Mismatch Error rate: change in nucleotides type.
- Two sample t-test was used to determine if there was statistical difference between N2 and PRDE1 samples, with p-value,< 0.05 considered significant.

## STATISTICAL ANALYSIS

### Statistical analysis (RNA-sequencing data):

Anova with posthoc Tukey HSD was conducted to compare distribution of Gene Length in the Differential Expressed Genes, with adjusted p-value <0.05 was considered significant. Wilcoxon test was used to compare the expression of the piRNAs, difference between the means was considered significant if the p-value <0.05. Hypergeometric test was done using the phyper function in R^29^, overlap was considered significant if p-value <0.05. Pearson’s Chi-squared test with Yates’ correction was used to determine if the number of up- or down-regulated splicing events during heat shock differed between the N2 and PRDE1 strains (p-value <0.05). All the statistical analyses were done in R^29^. Kolmogorov–Smirnov test was used to compare the probability distributions of mRNA error rates in the pileup analysis.

### Statistical analysis (qPCR data and phenotypes):

The data were analyzed by using Student t-test (two-tailed unpaired t-test) or 2 way-ANOVA (Fisher’s LSD test; GraphPad Prism software) as described in respective figure legends. P values are indicated as follows: ∗p < 0.05; ∗∗p < 0.01; ∗∗∗p < 0.001.

### Statistical analysis (other):

The statistical analyses for the remaining datasets are indicated in the Figure Legends.

## Supplementary Material

Supplement 1

## Figures and Tables

**Method Fig 1.**
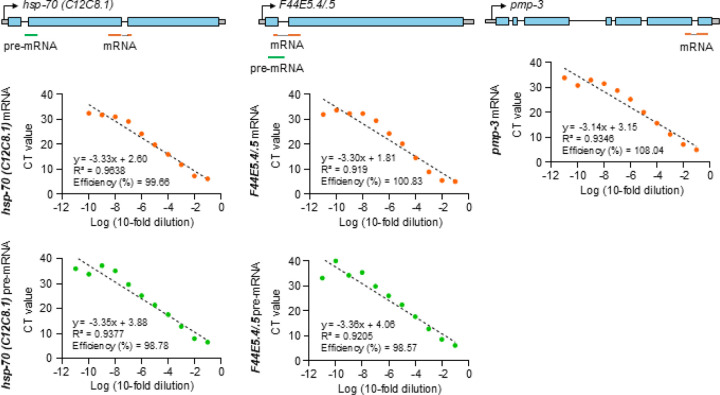
Standard curves for hsp-70 mRNA and pre-mRNA and pmp-3

**Table 1.** Strain information

Strain name	Genotype		Source	Note
N2	Bristol N2; Wild-type		CGC	
sx2499	*prde-1(mj207) V*		CGC	
PS3551	*hsf-1 (sy441) I*		CGC	
FLAG::HSF-1	*3X flag::hsf-1 I*	CRISPR /Cas-9	Prahlad Lab	
FLAG::HSF-1 (*sy441*)	*3X flag::hsf-1(sy441) I*	CRISPR /Cas-9	Prahlad Lab	
WM274	*prg-1(tm872) I; neSi14 [FLAG::prg-1 + Cbr-unc-119(+)] II; unc-119(ed3) III.*		CGC	
JDW10	wrdSi3 [sun-1p::TIR1::F2A::mTagBFP2::AID*::NLS ::tbb-2 3’UTR] II		CGC	
VP27	*prde-1::AID (syb7464) V*	CRISPR /Cas-9	Prahlad Lab /SunyBiotech	
JDW10 ;VP27	wrdSi3 [sun-1p::TIR1::F2A::mTagBFP2::AID*::NLS ::tbb-2 3’UTR] II; prde-1::AID (syb7464) V	Cross	Prahlad Lab	JDW10 was selecte d by picking BFP positive worms
VP01	*C12C8.1::FLAG (syb2141) I*	CRISPR /Cas-9	Prahlad Lab / SunyBiotech	
VP01; sx2499	*C12C8.1::FLAG (syb2141) I; prde-1(mj207) V*	Cross	Prahlad Lab	

**Table 2.** Primer information

Experiments	Name	Forward	Reverse
Genotype	VP01	CTCAAGCTGATATTGATAGA	AGCTAATTGTACAGAGAAAA
	VP27 (Outside AID insertion)	CGTCAAATATGTTGCGCATT	AAAATTCATTCCGCAGCAAC
	VP27 (Inside AID insertion)	GGTTTCCTGCCAAAAATCAA	AAAATTCATTCCGCAGCAAC
	sx2499	GAAATCGCAAAGGACACGAT	CGGCTTGTCACACACAAGAT
Multiple Experiments	*hsp-70 (C12C8.1)* pre-mRNA	TTTATTTTCCGTTTTCAGGTTGA	CCACGTAGGAAGGCGTAGTC
	*hsp-70 (C12C8.1)* mRNA	TTGGTTGGGGGATCAACTCG	GAGCAGTTGAGGTCCTTCCC
	*F44E5.4/.5* pre-mRNA	CGGTACTACGTACTCGTGTGTTG	TGTTATTAAAATTGAAAATGCCAAA
	*F44E5.4/.5* mRNA	CTATCAGAATGGAAAGGTTGAG	TCTTTCCGTATCTGTGAATGCC
	*hsp-16.11* pre-mRNA	TGAATCTTCTGAGGTAAGTAACACG	GCTTGAACTGCGAGACATTG
	*hsp-16.11* mRNA	CTCCATCTGAATCTTCTGAGATTGT	TCTGGCTTGAACTGCGAGAC
	*pmp-3* mRNA	TAGAGTCAAGGGTCGCAGTG	ATCGGCACCAAGGAAACTGG
PCR for generating Standard Curve	*hsp-70 (C12C8.1) (*cDNA)	CCCGTTGTTGAGGTTGAAGT	CGGCAGCTGATACATTGAGA
	*hsp-70 (C12C8.1)* (gDNA)	ACGTACTCATGTGTCGGTAT	CGGCAGCTGATACATTGAGA
	*F44E5.4/.5* (cDNA)	CTATCAGAATGGAAAGGTTGAG	TCACAAGCAGTTCGGAGACG
	*F44E5.4/.5* (gDNA)	TAAAAGGGCTGGGATTCGGG	TCACAAGCAGTTCGGAGACG
	*pmp-3* (cDNA)	TGGAGACAGTGGATGTGGAA	GAAAGAACTGTATCGGCACCA
RIP-RT-qPCR	*bath-45* mRNA	ACGATAGGCCTTTTCCAATG	GAGCTGCCTTTTCCAAATTC
ChIP-qPCR	*hsp-70 (C12C8.1)* TSS	ACGTACTCATGTGTCGGTAT	TCTTCTTCCAGTTTACATA ATCCT
	*hsp-70 (C12C8.1)* End	ACAATTCGCAATGAGAAGG GACG	GCATCTTCTGCTGATAACA GTGATC
	*F44E5.4/.5* TSS	TAAAAGGGCTGGGATTCGGG	ACCGAGGTCGATACCAATAGC
	*F44E5.4/.5* End	TTGATGAAACACTTCGTTGGTTGG	TCCAGCAGTTCCAGGATTTC
RT-qPCR	*T08G5.11*	CGATCAAGAAGGCGGAATCC	TCAACTCGAGACTGCCACAC
	*H27M09.6*	GCGATCACAAAGGCGGAATC	TCAACTCGAGACTGCCACAC
	*F08H9.10*	ATCAAGAAGGCGGAATCCCC	TCAACTCGAGACTGCCACAT
	*sup-6*	TGATCATGAAGACGGAATC CCC	TCAACTCGAGACTGCCACAC
	*sup-39*	GCCTACCCATTGCACTTTTGG	GCAGCCCCGATACGCAAAA
	*eft-3*	ACTTGATCTACAAGTGCGGAGGA	AAAGATCCCTTACCCATCTCCTG
	*linc-9*	AAGACTACGCCCTGGAC	CTTCTCGAATTGTAGTGGTCG
	*nhr-109*	GCAGGGAATGAAGGATTATACG	AATGGAATGTAGGAGTTGCG
	*B0495.8*	GAGTATGTCCTCACGATATGG	GTTTACGATACGCTCGCAG
	*F53B2.8*	GGATTTGGGAGATGCTATGAG	CGTACCCTTTTCTGGTGTTTC
	*Y39B6A.21*	GCCCTGGATAGTGTTAATTGTG	GCTGTCTTTGTCTCCGAAAAG

* Genotyping of sx2499 mutant was verified through digestion with MuCl.

**Table 3.** Primer information (small RNA)

Experiments	Name	Sequence	Targeting 22G RNA sequence
22G RNA FISH	*F44E5.4/.5* 22G RNA	ATCAGTTTGTCAATCAATTCTC	GAGAATTGATTGAC AAACTGAT
sRNA RT-qPCR	*hsp-70 (C12C8.1)* **Stem-loop (SL)**	CTCAACTGGTGTCGTGGAGTCGGC AATTCAGTTGAGTCTATTCG	GCACGTGTGATTTT CGAATAGA
	*hsp-70 (C12C8.1)* 22G RNA **Forward**	ACACTCCAGCTGGGGCACGTGTGA TTTTCG	
	*F44E5.4/.5* **Stem-loop (SL)**	CTCAACTGGTGTCGTGGAGTCGGC AATTCAGTTGAGATCAGTTT	GAGAATTGATTGAC AAACTGAT
	*F44E5.4/.5* 22G RNA **Forward**	ACACTCCAGCTGGGGAGAATTGATT GACAA	
	Universal **Reverse** primer	CTCAAGTGTCGTGGAGTCGGCAA	
Synthetic RNA	21ur-2348	UGAUGUCCCAUGUCGCAAUUC	

* synthetic 21ur-2348 RNA has 5’ phosphorylation at 5’ end and 2’ O-methyl bases at 3’ end

**Table 4.** UMI Family Size

SAMPLE	FAMILY SIZE		NUMBER OF UMI-FAMILIES			PERCENTAGE OF FAMILIES	
**N2.30MIN.HS.1**	1		24524361			75.959	
**N2.30MIN.HS.1**	2		5400148			16.7258	
**N2.30MIN.HS.1**	3		1336034			4.1381	
**N2.30MIN.HS.1**	4		451271			1.3977	
**N2.30MIN.HS.1**	5		203684			0.6309	
**N2.30MIN.HS.1**	6		106590			0.3301	
**N2.30MIN.HS.1**	7		70936			0.2197	
**N2.30MIN.HS.1**	8		43942			0.1361	
**N2.30MIN.HS.1**	9		31135			0.0964	
**N2.30MIN.HS.1**	10		22472			0.0696	
**N2.30MIN.HS.1**	11		16931			0.0524	
**N2.30MIN.HS.1**	12		12091			0.0374	
**N2.30MIN.HS.1**	13		10270			0.0318	
**N2.30MIN.HS.1**	14		7700			0.0238	
**N2.30MIN.HS.1**	15		6294			0.0195	
**N2.30MIN.HS.2**	1		30086473			70.356	
**N2.30MIN.HS.2**	2		8174687			19.1162	
**N2.30MIN.HS.2**	3		2454899			5.7407	
**N2.30MIN.HS.2**	4		896515			2.0965	
**N2.30MIN.HS.2**	5		410860			0.9608	
**N2.30MIN.HS.2**	6		208172			0.4868	
**N2.30MIN.HS.2**	7		141249			0.3303	
**N2.30MIN.HS.2**	8		87246			0.204	
**N2.30MIN.HS.2**	9		62244			0.1456	
**N2.30MIN.HS.2**	10		45576			0.1066	
**N2.30MIN.HS.2**	11		34216			0.08	
**N2.30MIN.HS.2**	12		24578			0.0575	
**N2.30MIN.HS.2**	13		21183			0.0495	
**N2.30MIN.HS.2**	14		15921			0.0372	
**N2.30MIN.HS.2**	15		12649			0.0296	
**N2.30MIN.HS.3**	1		25262426			77.3577	
**N2.30MIN.HS.3**	2		5177514			15.8544	
**N2.30MIN.HS.3**	3		1237052			3.7881	
**N2.30MIN.HS.3**	4		423263			1.2961	
**N2.30MIN.HS.3**	5		195074			0.5973	
**N2.30MIN.HS.3**	6		101639			0.3112	
**N2.30MIN.HS.3**	7		69452			0.2127	
**N2.30MIN.HS.3**	8		43029			0.1318	
**N2.30MIN.HS.3**	9		30368			0.093	
**N2.30MIN.HS.3**	10	22085					0.0676
**N2.30MIN.HS.3**	11	16736					0.0512
**N2.30MIN.HS.3**	12	11993					0.0367
**N2.30MIN.HS.3**	13	10153					0.0311
**N2.30MIN.HS.3**	14	7569					0.0232
**N2.30MIN.HS.3**	15	6034					0.0185
**PRDE1.30MIN.HS.1**	1	28484889					75.4403
**PRDE1.30MIN.HS.1**	2	6316795					16.7296
**PRDE1.30MIN.HS.1**	3	1615033					4.2773
**PRDE1.30MIN.HS.1**	4	566822					1.5012
**PRDE1.30MIN.HS.1**	5	266046					0.7046
**PRDE1.30MIN.HS.1**	6	139724					0.37
**PRDE1.30MIN.HS.1**	7	96951					0.2568
**PRDE1.30MIN.HS.1**	8	59486					0.1575
**PRDE1.30MIN.HS.1**	9	42911					0.1136
**PRDE1.30MIN.HS.1**	10	31353					0.083
**PRDE1.30MIN.HS.1**	11	23826					0.0631
**PRDE1.30MIN.HS.1**	12	16649					0.0441
**PRDE1.30MIN.HS.1**	13	14570					0.0386
**PRDE1.30MIN.HS.1**	14	11210					0.0297
**PRDE1.30MIN.HS.1**	15	8782					0.0233
**PRDE1.30MIN.HS.2**	1	28896617					74.5928
**PRDE1.30MIN.HS.2**	2	6660140					17.1923
**PRDE1.30MIN.HS.2**	3	1744353					4.5028
**PRDE1.30MIN.HS.2**	4	613302					1.5832
**PRDE1.30MIN.HS.2**	5	285714					0.7375
**PRDE1.30MIN.HS.2**	6	148717					0.3839
**PRDE1.30MIN.HS.2**	7	102995					0.2659
**PRDE1.30MIN.HS.2**	8	63467					0.1638
**PRDE1.30MIN.HS.2**	9	45498					0.1174
**PRDE1.30MIN.HS.2**	10	33617					0.0868
**PRDE1.30MIN.HS.2**	11	25055					0.0647
**PRDE1.30MIN.HS.2**	12	17947					0.0463
**PRDE1.30MIN.HS.2**	13	15305					0.0395
**PRDE1.30MIN.HS.2**	14	11683					0.0302
**PRDE1.30MIN.HS.2**	15	9226					0.0238
**PRDE1.30MIN.HS.3**	1	28795499					74.5599
**PRDE1.30MIN.HS.3**	2	6667426					17.2639
**PRDE1.30MIN.HS.3**	3	1736294					4.4958
**PRDE1.30MIN.HS.3**	4	605894					1.5688
**PRDE1.30MIN.HS.3**	5	280303					0.7258
**PRDE1.30MIN.HS.3**	6	145531					0.3768
**PRDE1.30MIN.HS.3**	7	101463					0.2627
**PRDE1.30MIN.HS.3**	8			62804	0.1626		
**PRDE1.30MIN.HS.3**	9			44520	0.1153		
**PRDE1.30MIN.HS.3**	10			33263	0.0861		
**PRDE1.30MIN.HS.3**	11			25169	0.0652		
**PRDE1.30MIN.HS.3**	12			17905	0.0464		
**PRDE1.30MIN.HS.3**	13			15608	0.0404		
**PRDE1.30MIN.HS.3**	14			11845	0.0307		
**PRDE1.30MIN.HS.3**	15			9208	0.0238		

## Data Availability

The data for this study have been deposited in the European Nucleotide Archive (ENA) at EMBL-EBI under accession numbers PRJEB101605 (https://www.ebi.ac.uk/ena/browser/view/PRJEB101605), PRJEB101594 (https://www.ebi.ac.uk/ena/browser/view/PRJEB101594), PRJEB101181(https://www.ebi.ac.uk/ena/browser/view/PRJEB101181) .
