# Supplementary material for: piRNAs safeguard splicing and RNA fidelity during heat shock
Source: bioRxiv. 2025 Dec 10:2025.10.27.684940. Originally published 2025 Oct 28. Preprint. [Version 2] doi: 10.1101/2025.10.27.684940 (PMC12636506; doi:10.1101/2025.10.27.684940)
Supplement: Supplement 1 [file NIHPP2025.10.27.684940v2-supplement-1.pdf]

Suppl. Figure 1

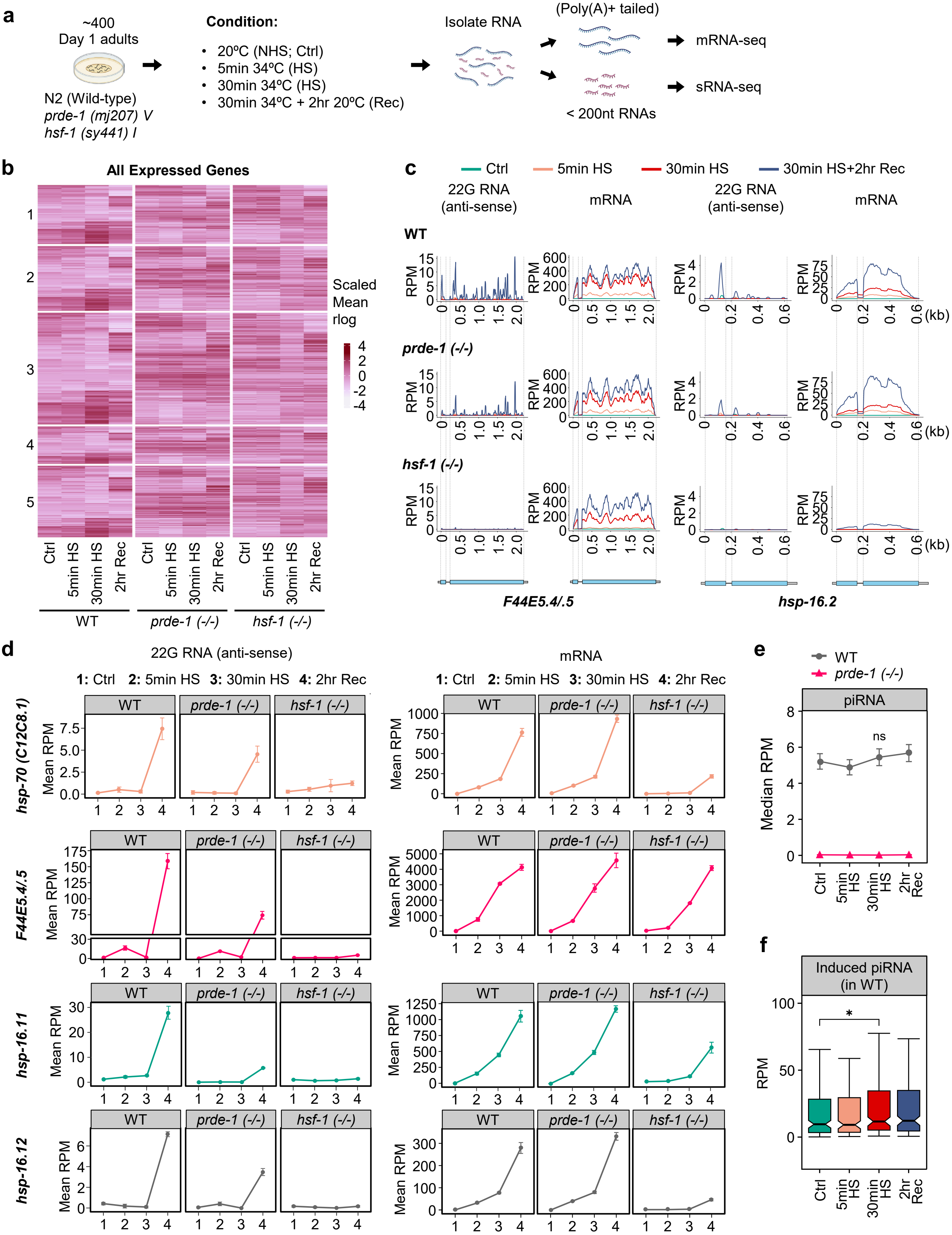

bioRxiv preprint doi: https://doi.org/10.1101/2025.10.27.684940; this version posted December 10, 2025. The copyright holder for this preprint (which was not certified by peer review) is the author/funder, who has granted bioRxiv a license to display the preprint in perpetuity. It is made available under aCC-BY 4.0 International license.

Supplementary Figure 1. Piwi-interacting RNAs (piRNAs) target *hsp* mRNAs upon heat shock.

a. RNA-sequencing experiment schematic.

b. Heatmaps: Wild type (N2; WT, left), *prde-1* (middle), and *hsf-1* (right). Mean expression values of the regularized log (rlog) transformed counts for all expressed genes shown for each strain. Expression scaled row-wise (z-score) to highlight relative changes. K-means clustering was performed on the gene expression of the WT samples, and gene order and clustering (1-5) are preserved across strains and treatments. Columns: treatments [Non heat shocked control (NHS; Ctrl), 5 minutes heat shock (5min HS), 30 minutes heat shock (30min HS), and 2 hours recovery (2hr Rec)]. Color bar: scaled expression level.

c. Metaprofile plots showing normalized antisense 22G-RNA reads (RPM), and mRNA reads (RPM) across *hsp* mRNAs in wild-type (WT), *prde-1* and *hsf-1* mutants. **Top**: Conditions of heat shock. **Bottom**: Schematic of *hsp* genes analyzed.

d. Left: Normalized expression (RPM) of 22G-RNAs antisense to *hsp* genes. **Right:** Normalized expression (PRM) of *hsp* mRNAs in WT, *prde*-1, and *hsf-1* mutants. Y-axis: Expression (RPM). X-axis: Treatments (see top legend).

e. Normalized expression (RPM) of piRNAs in WT and *prde*-1 mutants. RPM >0.5 in at least one sample and RPM >0 in at least replicates were counted. Statistical analysis: Wilcoxon test.

bioRxiv preprint doi: https://doi.org/10.1101/2025.10.27.684940; this version posted December 10, 2025. The copyright holder for this preprint (which was not certified by peer review) is the author/funder, who has granted bioRxiv a license to display the preprint in perpetuity. It is made available under aCC-BY 4.0 International license.

f. Boxplot showing normalized expression (RPM) of significantly differentially expressed piRNAs between the WT 30 minutes heat shock and control conditions. Boxplots: median value depicted. Y-axis: Expression (RPM). X-axis: Treatments. Statistical analysis: Wilcoxon test.

Suppl. Figure 2

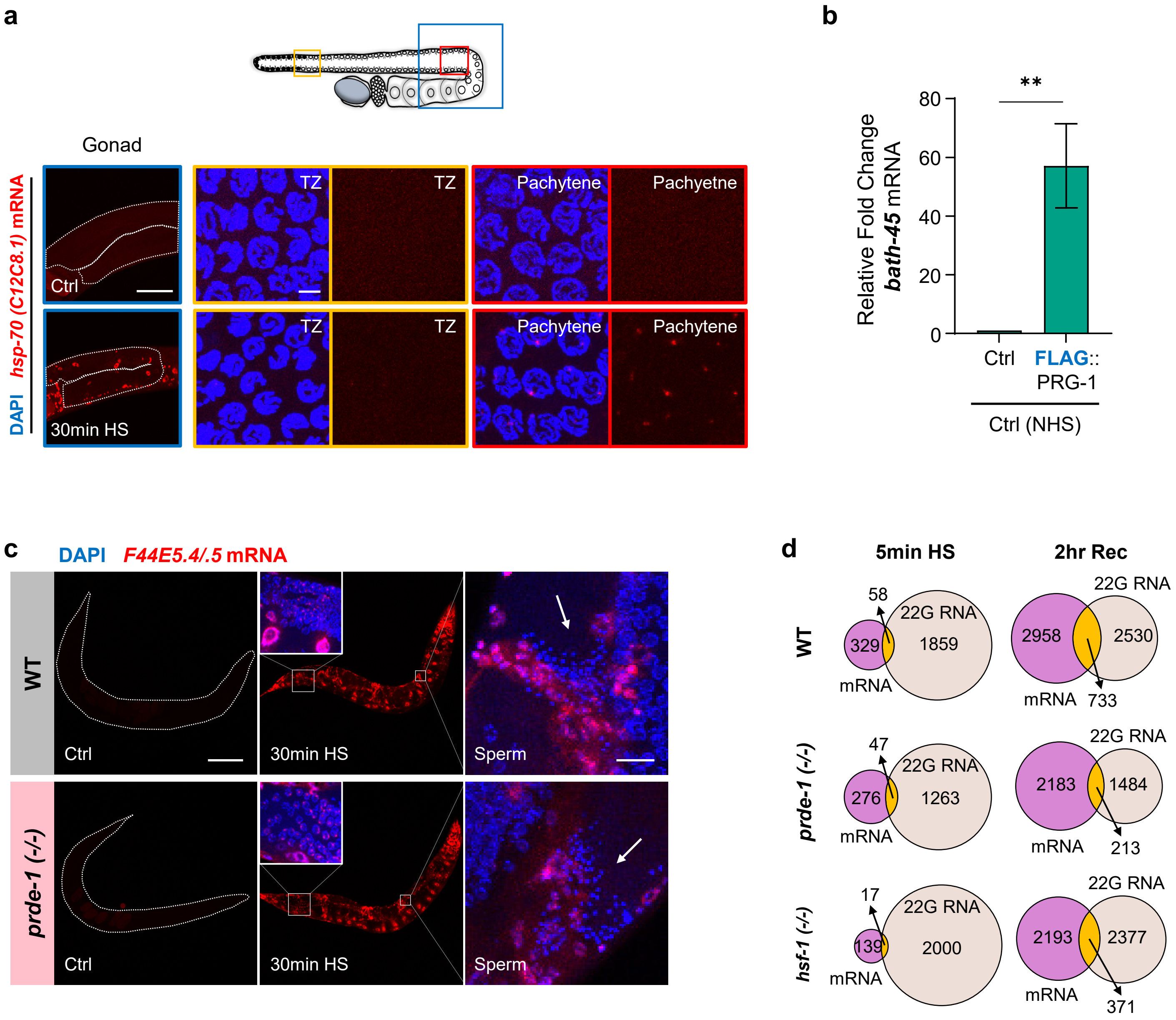

bioRxiv preprint doi: https://doi.org/10.1101/2025.10.27.684940; this version posted December 10, 2025. The copyright holder for this preprint (which was not certified by peer review) is the author/funder, who has granted bioRxiv a license to display the preprint in perpetuity. It is made available under aCC-BY 4.0 International license.

Supplementary Figure 2. piRNAs target *hsp* mRNAs expressed in the germline upon heat shock to generate 22G-RNAs.

a. Representative micrographs of projected confocal Z-sections of regions (Gonad, TZ, and Pachytene) showing RNA Fluorescence *in situ* hybridization (RNA FISH) of *hsp-70 (C12C8.1)* in WT animals. Red: *hsp-70 (C12C8.1)* mRNA. Blue: DNA staining with DAPI. Similar to Fig 1b, c. Scale bars: 50 μm (Gonad) and 3 μm (TZ, Pachytene).

b. *bath-45* immunoprecipitated with PRG-1 in non-heat shocked animals serving as a positive control for RIP experiments in Fig 1e. Bars: mean value. Error bars: standard error. n = 4 biologically independent experiments. Statistical analysis: two-tailed unpaired t-test.

c. Representative micrographs of projected confocal Z-sections showing RNA FISH of *hsp-70 (F44E5.4/.5)* mRNA in WT and *prde-1* animals upon non heat shock (Ctrl) or heat shock. Note lack of expression in sperm. Compare with 22G-RNA expression in Fig 1h. Red: *hsp-70 (F44E5.4/.5)* mRNA. Blue: DAPI stained DNA. Scale bars: 100 μm (Ctrl, 30min HS) and 10 μm (Sperm).

d. Venn diagrams showing overlap between differentially expressed mRNAs and 22G-RNAs upon 5 minutes heat shock, and for 2 hours recovery from heat shock in WT and mutant backgrounds.

Suppl. Figure 3

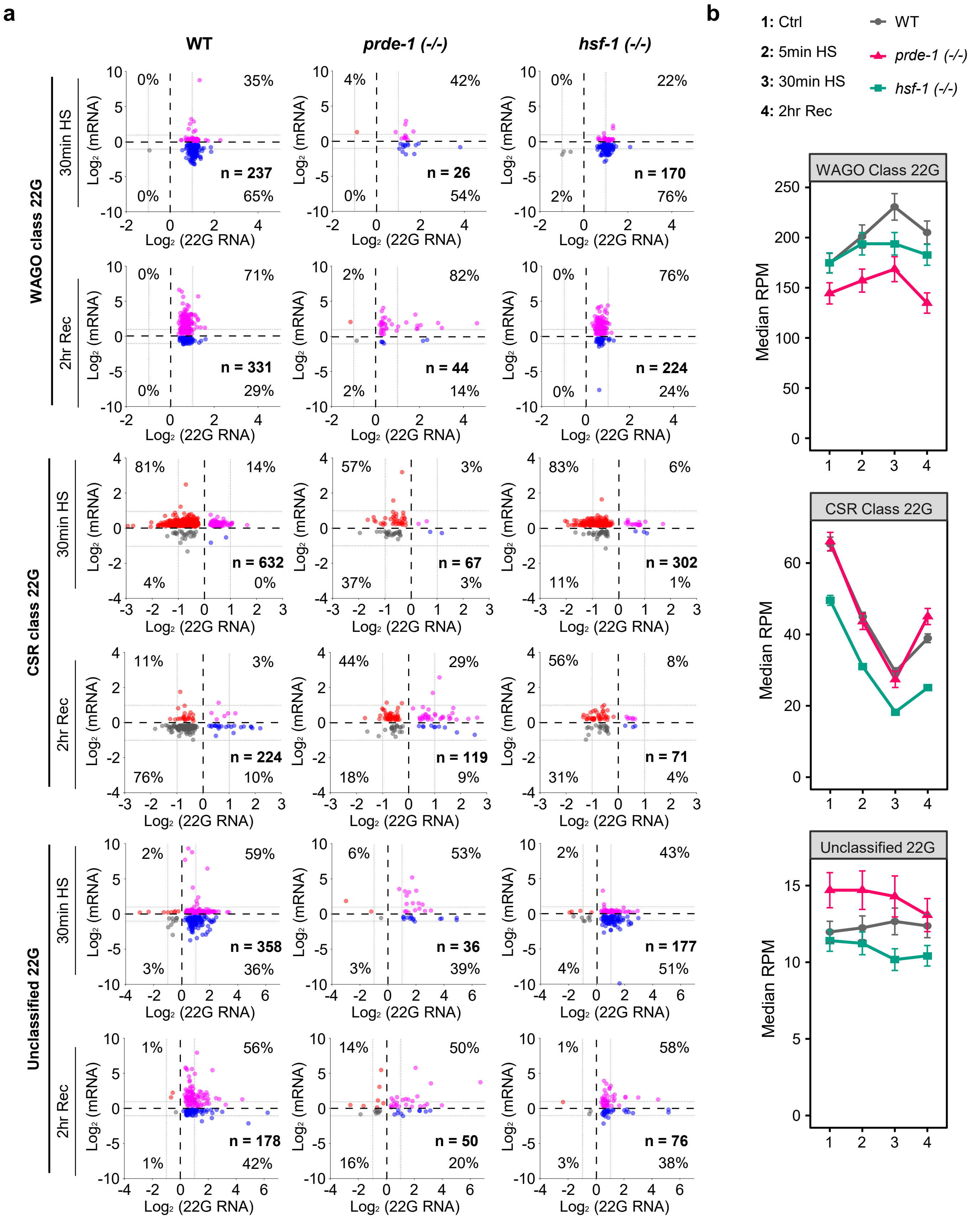

bioRxiv preprint doi: https://doi.org/10.1101/2025.10.27.684940; this version posted December 10, 2025. The copyright holder for this preprint (which was not certified by peer review) is the author/funder, who has granted bioRxiv a license to display the preprint in perpetuity. It is made available under aCC-BY 4.0 International license.

Supplementary Figure 3. piRNA-dependent WAGO and CSR-1 22G-RNAs and unclassified 22G-RNAs are templated from genes differentially expressed upon heat shock.

a. Scatter plot comparing the $\log_2$ fold-changes in WAGO class, CSR-1 class, and unclassified 22G-RNAs and complementary mRNAs in WT, *prde-1,* and *hsf-1*, upon 30min heat shock, and 2 hours recovery from heat shock. Number of mRNAs/category shown, and the % of mRNA in each quadrant of scatter plot shown.

b. Normalized expression (RPM) of WAGO and CSR-1 interacting 22G-RNAs, and unclassified 22G-RNAs in WT, *prde*-1, and *hsf-1* mutants. Y-axis: Expression (RPM). X-axis: Treatments (see top legend).

Suppl. Figure 4

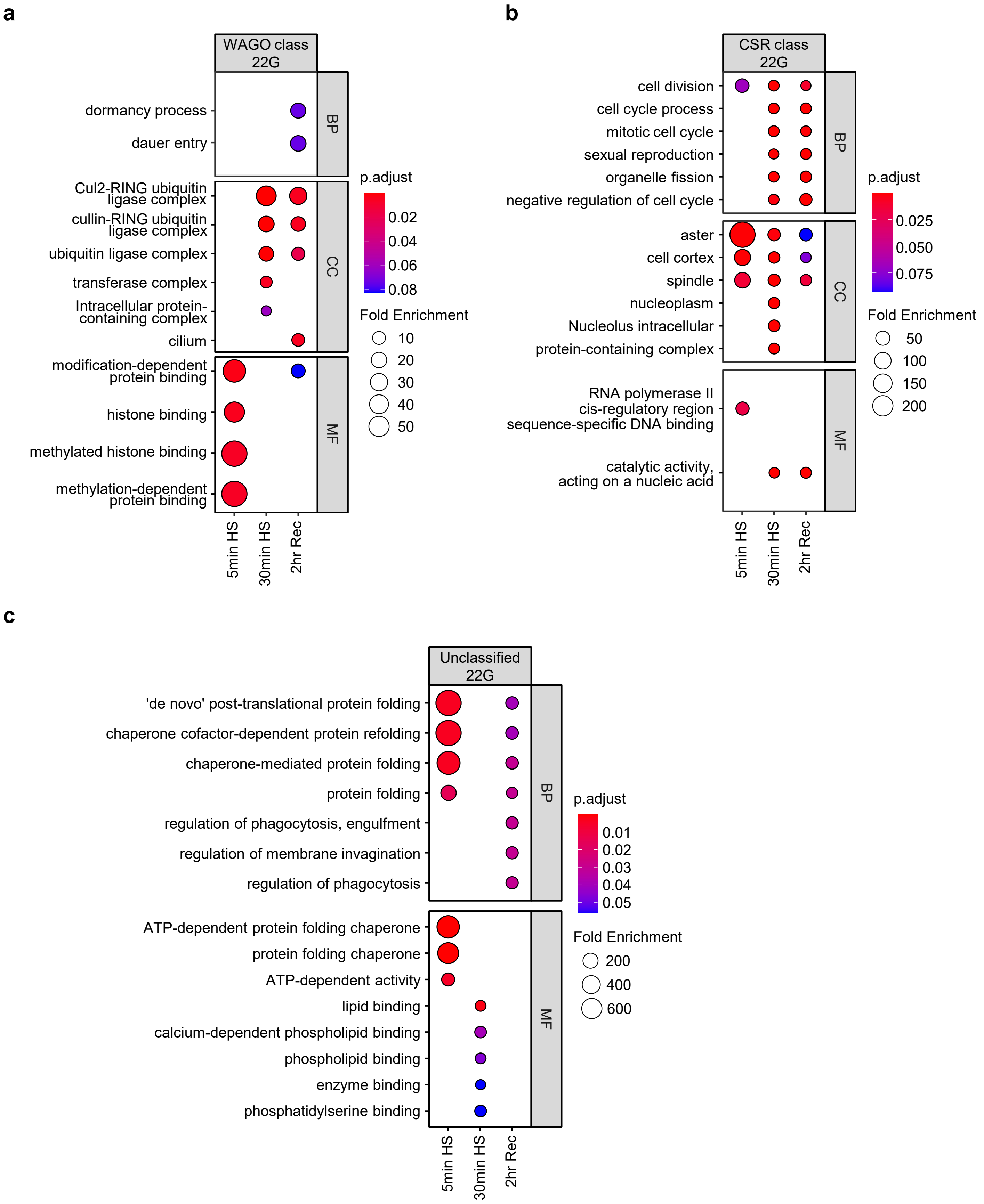

bioRxiv preprint doi: https://doi.org/10.1101/2025.10.27.684940; this version posted December 10, 2025. The copyright holder for this preprint (which was not certified by peer review) is the author/funder, who has granted bioRxiv a license to display the preprint in perpetuity. It is made available under aCC-BY 4.0 International license.

Supplementary Figure 4. 22G-RNAs produced upon heat shock target specific categories of mRNAs.

a-c. Gene Ontology analysis for **(a)** WAGO, **(b)** CSR-1, and **(c)** Unclassified 22G-RNA targets in WT animals across the different treatment conditions. All *hsp* genes are targets of unclassified 22G-RNAs (see Suppl. Table 4).

Suppl. Figure 5

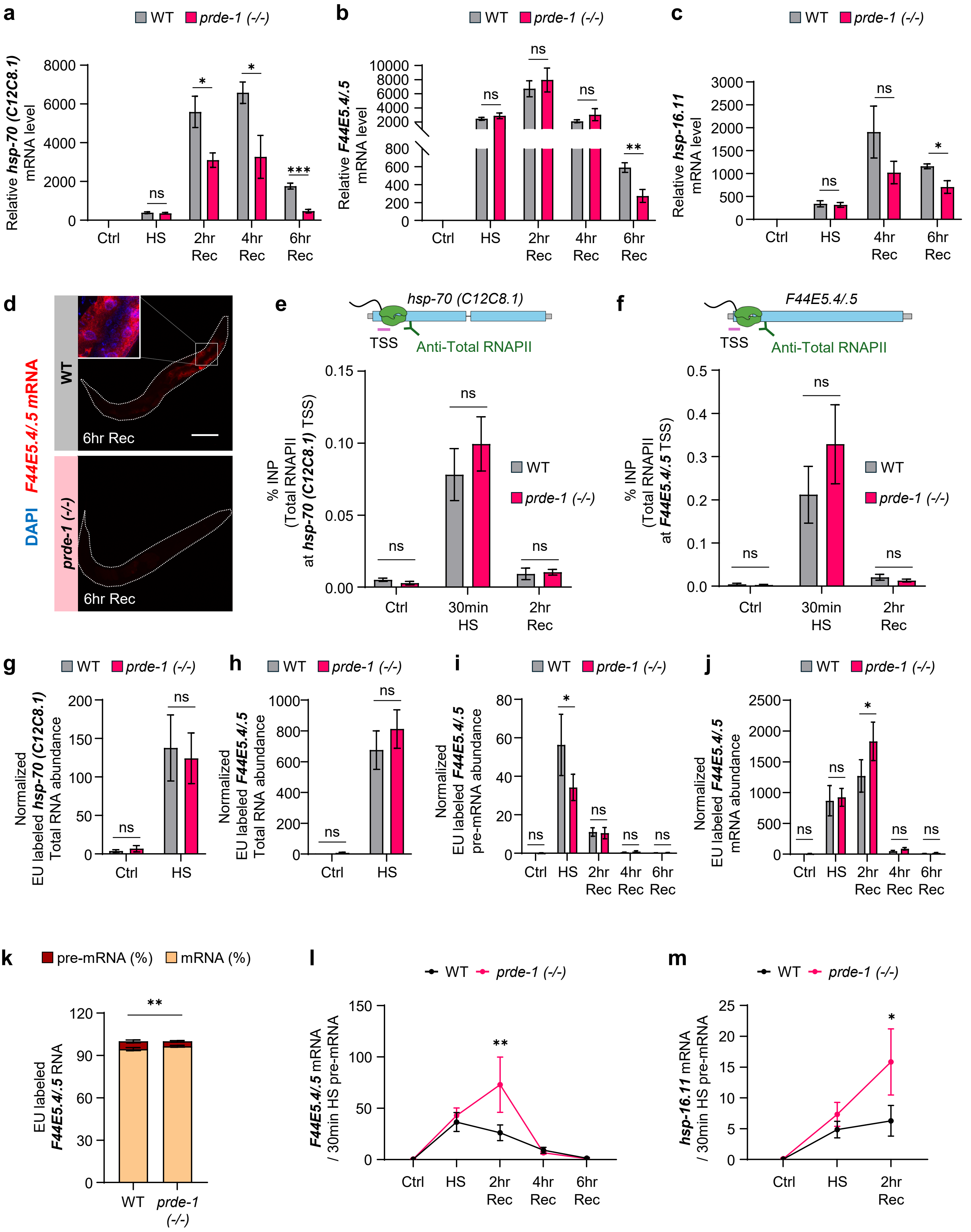

bioRxiv preprint doi: https://doi.org/10.1101/2025.10.27.684940; this version posted December 10, 2025. The copyright holder for this preprint (which was not certified by peer review) is the author/funder, who has granted bioRxiv a license to display the preprint in perpetuity. It is made available under aCC-BY 4.0 International license.

Supplementary Figure 5. The piRNA pathway protects *hsp* mRNAs persistence

a-c. RT-qPCR of relative fold change of *hsp-70s* (*C12C8.1* and *F44E5.4/.5*) and *hsp-16.11* mRNAs levels measured upon heat shock and recovery. mRNA values were normalized to *pmp-3* and shown relative to control (non-heat shocked) animals of each strain. Bars: mean value. Error bars: standard error. n = 3-13 biologically independent experiments. Statistical analysis: two-tailed unpaired t-test.

d. Representative micrographs of projected confocal Z-sections showing RNA FISH of *hsp-70 (F44E5.4/.5)* mRNA in WT and *prde-1* animals after 6 hours following heat shock. Red: *hsp-70 (F544E5.4/.5)* mRNA. Blue: DNA staining with DAPI. Scale bars: 100 µm

e, f. Total RNA polymerase II levels at *hsp* genes. **Top**: Schematic of *hsp-70 (C12C8.1* and *F44E5.4/.5)* gene, and region probed Transcription Start Site (TSS). **Bottom**: ChIP-qPCR showing fold enrichment over input. Y-axis: % ChIP input. X-axis: Treatments. Bars: mean value. Error bars: standard error. n = 3-4 biologically independent experiments. Statistical analysis: 2-way ANOVA.

g, h. Total nascent RNA abundance. EU-labeled **(g)** *hsp-70 (C12C8.1)* and **(h)** *hsp-70 (F44E5.4/.5)* RNA abundance. Y-axis: Normalized abundance. X-axis: Treatments. Bars: mean value. Error bars: standard error. n = 10-12 biologically independent experiments. Statistical analysis: 2-way ANOVA.

i, j. EU-labeled *hsp-70 (F44E5.4/.5)* **(i)** pre-mRNA and **(j)** mRNA abundance. Y-axis: Normalized abundance. X-axis: Treatments. Bars: mean value. Error bars: standard error. n = 5-13 biologically independent experiments. Statistical analysis: 2-way ANOVA.

bioRxiv preprint doi: https://doi.org/10.1101/2025.10.27.684940; this version posted December 10, 2025. The copyright holder for this preprint (which was not certified by peer review) is the author/funder, who has granted bioRxiv a license to display the preprint in perpetuity. It is made available under aCC-BY 4.0 International license.

k. Percentages of *hsp-70 (F44E5.4/.5)* pre-mRNA and mRNA in total EU-labeled RNA after 30min heat shock. Bars: mean value. Error bars: standard error. n = 13 biologically independent experiments. Statistical analysis: two-tailed unpaired t-test.

l. EU-labeled spliced *hsp-70 (F44E5.4/.5)* mRNAs produced from the nascent EU-labeled unspliced *hsp-70 (F44E5.4/.5)* pre-mRNA. Y-axis: Relative abundance. X-axis: Treatments. Line graph: mean value. Error bars: standard error. n = 5-14 biologically independent experiments. Statistical analysis: 2-way ANOVA.

m. EU-labeled spliced *hsp-16.11* mRNAs produced from the nascent EU-labeled unspliced *hsp-16.11* pre-mRNA. Y-axis: Relative abundance. X-axis: Treatments. Line graph: mean value. Error bars: standard error. n = 6-8 biologically independent experiments. Statistical analysis: 2-way ANOVA.

Suppl. Figure 6

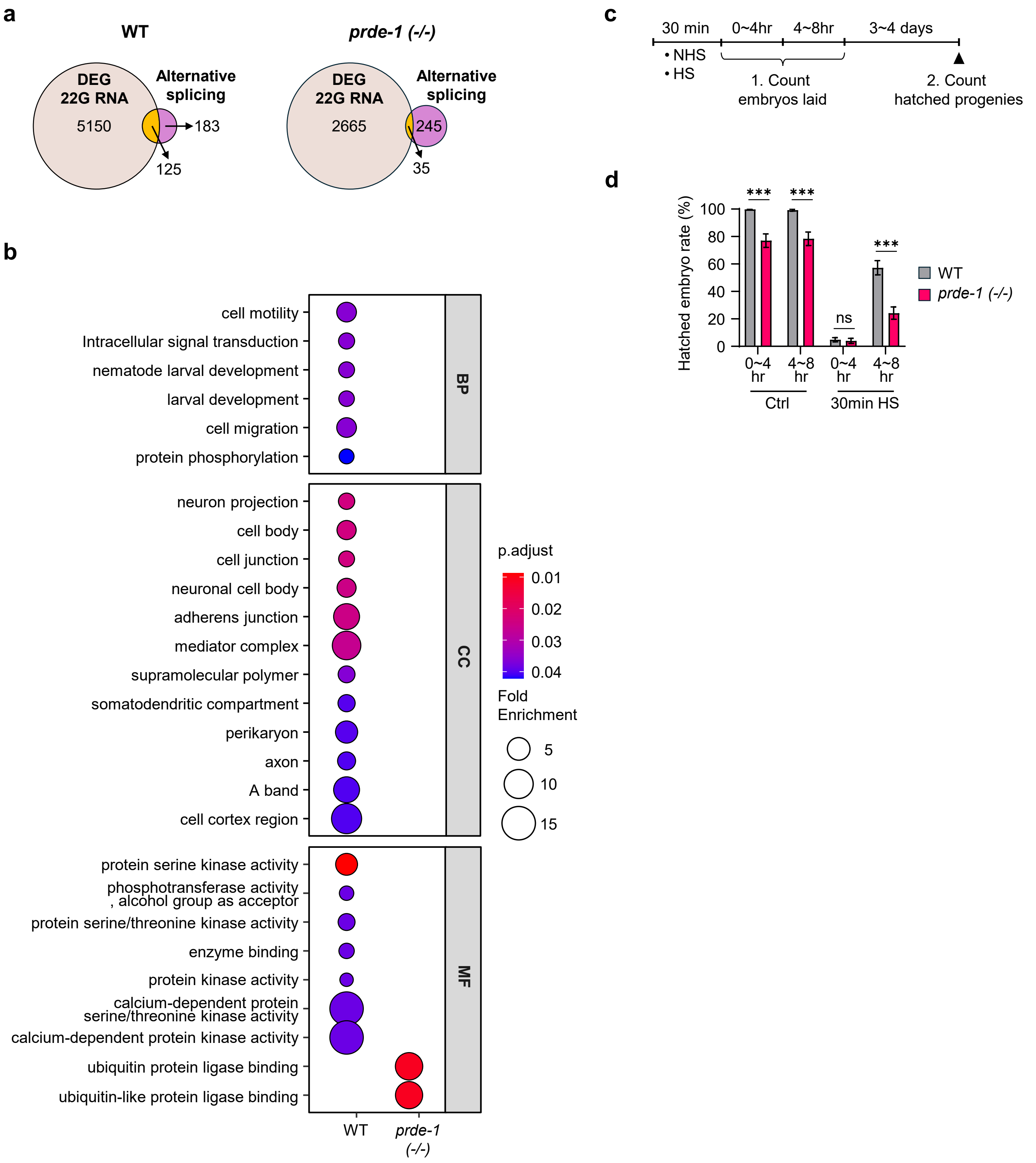

bioRxiv preprint doi: https://doi.org/10.1101/2025.10.27.684940; this version posted December 10, 2025. The copyright holder for this preprint (which was not certified by peer review) is the author/funder, who has granted bioRxiv a license to display the preprint in perpetuity. It is made available under aCC-BY 4.0 International license.

Supplementary Figure 6. Alternative splicing is affected in piRNA deficient (*prde-1*) animals.

a. Venn diagrams showing overlap between differentially expressed AS mRNAs and 22G-RNAs upon 30 minutes heat shock in WT and *prde-1* animals.

b. Gene Ontology analysis (p.adj <0.05) for AS mRNAs in WT and *prde-1* animals.

c, d. **(c)** Experiment schematic to assess recovery of fecundity in WT and *prde-1* animals after heat shock. NHS: non-heat shock. HS: heat shock. **(d)** Percentage of hatched embryos that laid over time during recovery from HS. Bars: mean value. Error bars: standard error. n = 7 biologically independent experiments. Statistical analysis: 2-way ANOVA.

Suppl. Figure 7

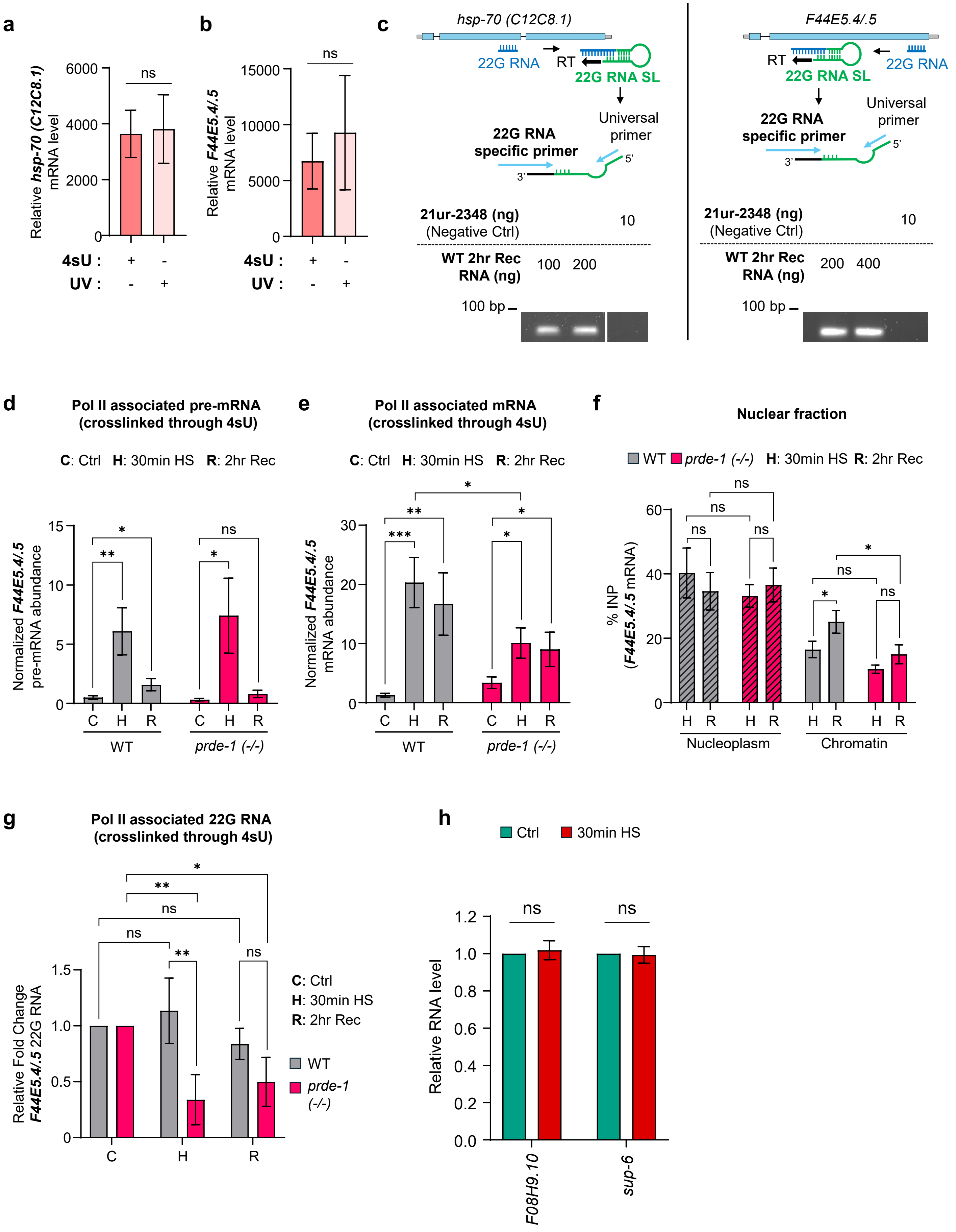

bioRxiv preprint doi: https://doi.org/10.1101/2025.10.27.684940; this version posted December 10, 2025. The copyright holder for this preprint (which was not certified by peer review) is the author/funder, who has granted bioRxiv a license to display the preprint in perpetuity. It is made available under aCC-BY 4.0 International license.

Supplementary Figure 7. 22G-RNAs associate with RNA Polymerase II and nascent *hsp* transcript to alter splicing.

a, b. 4sU treatment does not impair *hsp-70* expression. **(a)** *hsp-70 (C12C8.1)* mRNA and **(b)** *hsp-70 (F44E5.4/.5)* mRNA expressed upon heat shock in the presence and absence of either 4sU do not differ (UV exposure alone was used as comparison). Relative mRNA levels shown. mRNA values were normalized to *pmp-3* and shown relative to 4sU and UV-treated non-heat-shocked WT (value=1, not shown). Y-axis: Relative mRNA. X-axis: Treatment. Bars: mean value. Error bars: standard error. n = 3 biologically independent experiments. Statistical analysis: two-tailed unpaired t-test.

c. Stem loop RT-qPCR to amplify 22G-RNAs. **Top:** Cartoon depicting the 22G-RNA that was assayed, and complementary region on *hsp-70*. Universal primer, and 22G-RNA specific primers shown. **Bottom:** Representative agarose gel showing specificity. Different concentrations of total RNA containing 22G-RNAs were used to assess primer efficiency, and synthetically generated 21ur-2348 oligonucleotides were used as a negative control. See Materials and Methods for details. (expected PCR product size: 69bp)

d, e. Modified PAR-CLIP. Amounts of *hsp-70 (F44E5.4/.5)* **(d)** pre-mRNA and **(e)** mRNA that cross-linked to, and immunoprecipitated with Pol II normalized to *pmp-3* amounts. Y-axis: Normalized or relative abundance. X-axis: Treatment conditions (see top legend). Bars: mean value. Error bars: standard error. (d) n = 8-12 biologically independent experiments. Statistical analysis: two-tailed unpaired t-test; (e) n = 9-14 biologically independent experiments. Statistical analysis: two-tailed unpaired t-test (within strain) and 2 way-ANOVA (between strains).

bioRxiv preprint doi: https://doi.org/10.1101/2025.10.27.684940; this version posted December 10, 2025. The copyright holder for this preprint (which was not certified by peer review) is the author/funder, who has granted bioRxiv a license to display the preprint in perpetuity. It is made available under aCC-BY 4.0 International license.

f. Nuclear fractionation to assess amounts *hsp-70 (F44E5.4/.5)* RNA fate. Fold enrichment over input (nuclei) of *hsp-70 (F44E5.4/.5)* mRNA in respective fractions. Y-axis: % input (total nucleus). X-axis: Nuclear fraction, strain and treatment. Bars: mean value. Error bars: standard error. n = 9-10 biologically independent experiments. Statistical analysis: 2 way-ANOVA (within each fraction).

g. Amounts of *hsp-70* (*F44E5.4/.5*) 22G-RNA that cross-linked to, and immunoprecipitated with Pol II normalized to *pmp-3* amounts in the modified PAR-CLIP assay. Y-axis: Normalized abundance. X-axis: Treatments. Bars: mean value. Error bars: standard error. n = 4-6 biologically independent experiments. Statistical analysis: 2-way ANOVA.

h. Two other U1 RNA levels assessed upon heat shock. RT-qPCR of relative fold change in WT animals. Values normalized to *pmp-3* mRNA values and shown relative to non-heat-shock levels. Y-axis: Relative RNA levels. X-axis: U1RNAs. Bars: mean value. Error bars: standard error. n = 3 biologically independent experiments. Statistical analysis: 2-way ANOVA.

Suppl. Figure 8

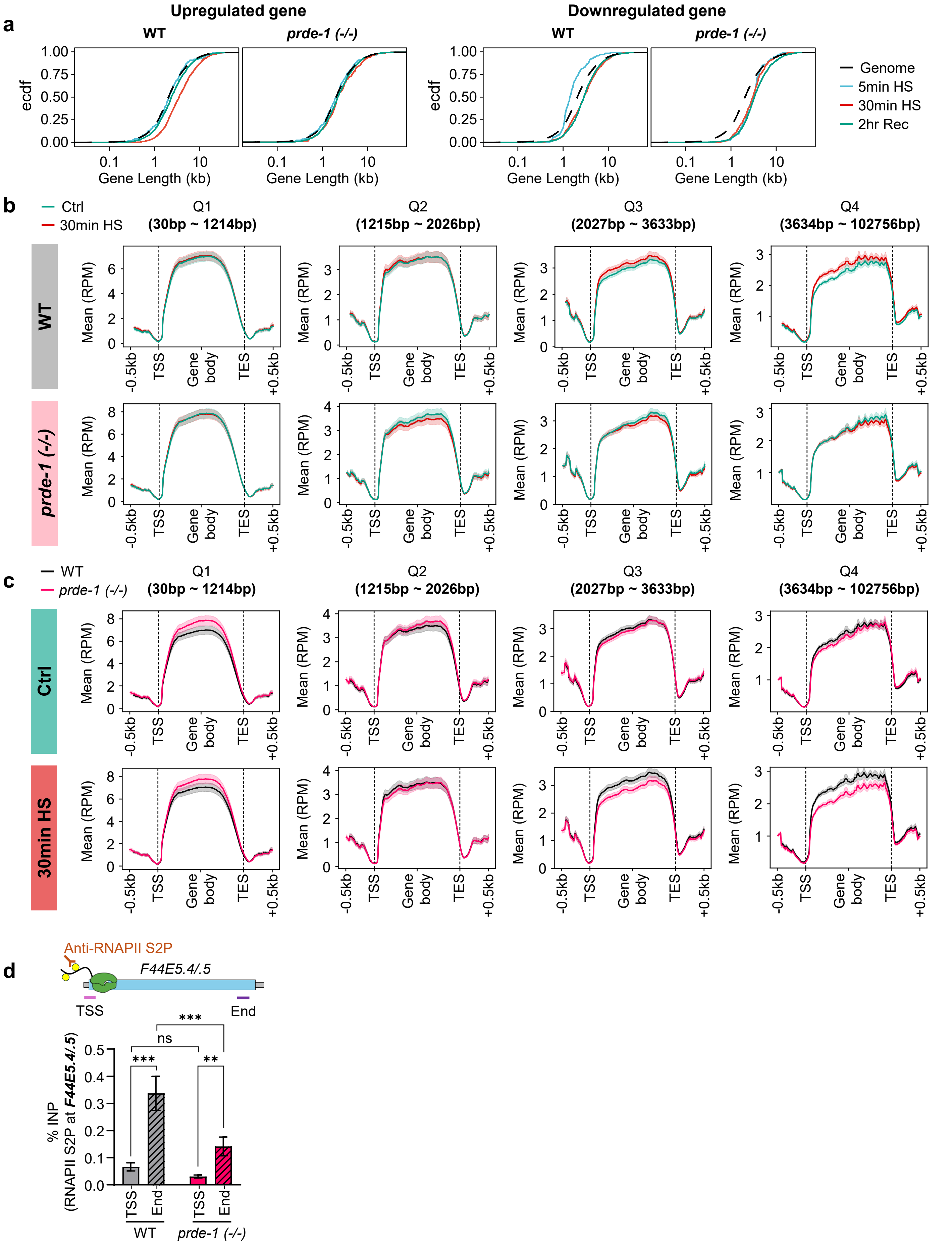

bioRxiv preprint doi: https://doi.org/10.1101/2025.10.27.684940; this version posted December 10, 2025. The copyright holder for this preprint (which was not certified by peer review) is the author/funder, who has granted bioRxiv a license to display the preprint in perpetuity. It is made available under aCC-BY 4.0 International license.

Supplementary Figure 8. piRNA pathway affects gene expression in a a gene-length dependent manner.

a. Empirical Cumulative Distribution Function (ECDF) plots, to visualize the proportion of gene expression values (mRNA-seq) as a function of gene lengths. Expression values of genes significantly upregulated and downregulated upon 30min heat shock (p.adj <0.05) in wild-type (WT) and *prde-1* are plotted. Y-axis: Cumulative proportion of reads. X-axis: Gene lengths. **Top:** Strain names and differential expression. Note bias towards increased gene lengths in WT, but not *prde-1* upregulated genes, but similar bias towards long genes in all downregulated genes.

b, c. Metagene plot showing mRNA expression (mRNA-seq) stratified by gene length compared between control and upon 30 min. heat shock, **(b)** within strains, and **(c)** between strains.

d. Elongating RNA polymerase II (anti-S2P) levels at *hsp* genes. **Top**: Schematic of *hsp-70* (*F44E5.4/.5*) gene, and regions probed: Transcription Start Site (TSS) and distal regions (3') of *hsp-70* (*F44E5.4/.5*). **Bottom:** ChIP-qPCR showing fold enrichment over input. Y-axis: % ChiP input. X-axis: Strain, and region assayed. Bars: mean value. Error bars: standard error. n = 8 (TSS) and 4 (End) biologically independent experiments. Statistical analysis: 2 way-ANOVA.

Suppl. Figure 9

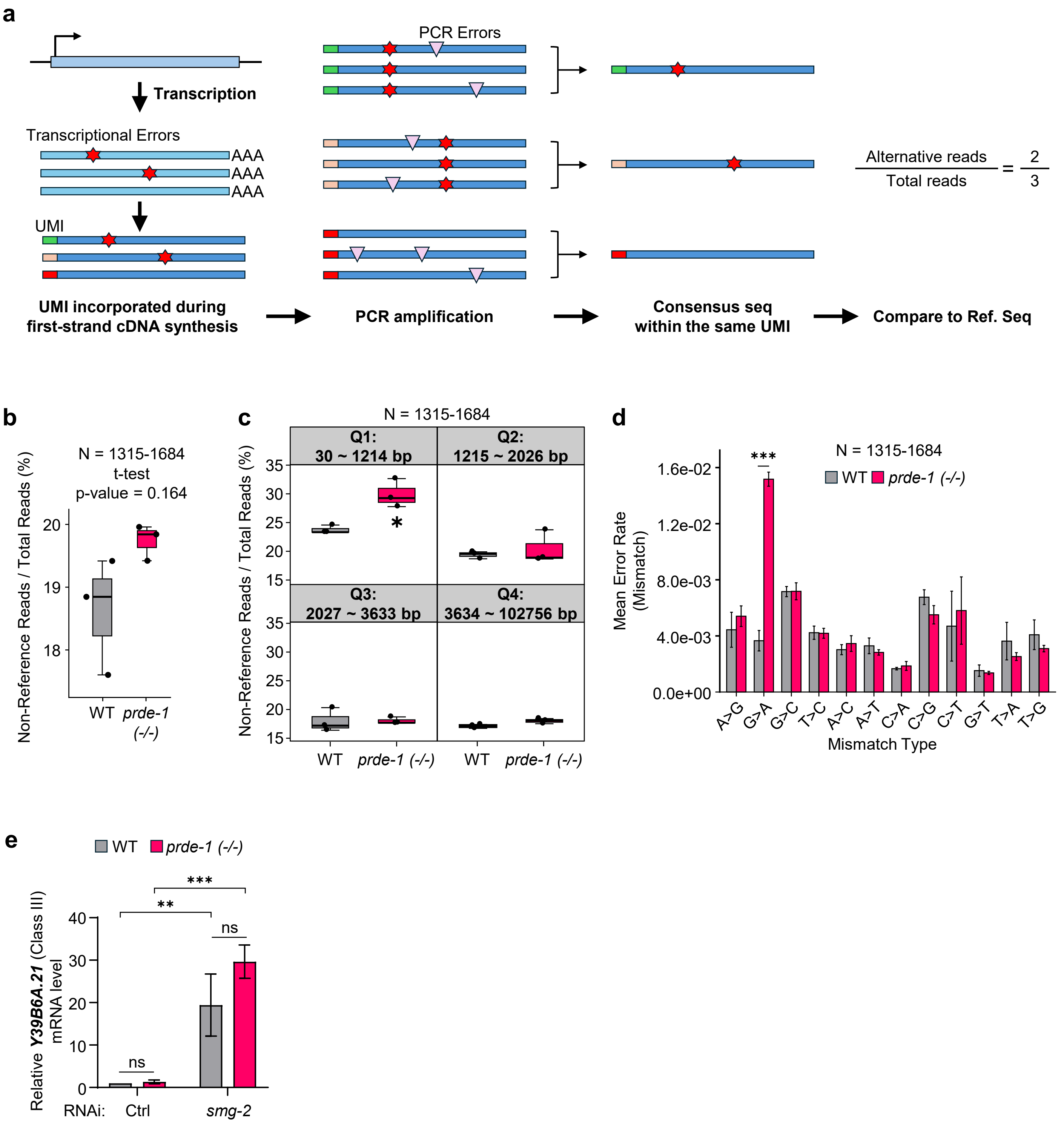

bioRxiv preprint doi: https://doi.org/10.1101/2025.10.27.684940; this version posted December 10, 2025. The copyright holder for this preprint (which was not certified by peer review) is the author/funder, who has granted bioRxiv a license to display the preprint in perpetuity. It is made available under aCC-BY 4.0 International license.

Supplementary Figure 9. piRNAs protect transcript fidelity.

a. Schematic of UMI labeling: mRNAs from wild-type and *prde-1* animals were isolated (some mRNA molecules, but not all, presumably contain errors introduced during transcription or splicing). mRNAs were labelled with UMIs during first-strand cDNA synthesis. UMI-labelled mRNAs were PCR amplified. This process also leads to the generation of PCR-based errors. The PCR-generated errors were distinguished from original errors in the mRNA by generating a consensus of UMI families. These consensus sequences were aligned to the *C.elegans* reference, mismatches identified, and the number of mRNA molecules that contained this mismatch/total number of mRNAs that mapped to that region (gene) were calculated to generate an error rate.

b. Percentage of variant-containing mRNA molecules in wild type and *prde-1* mutants (as identified by HaplotypeCaller and SnpEff). Y-axis: % variants. X-axis: Strain. Total variant containing sites=1315-1684. Approximately 3.71 x 10^7 individual mRNAs analyzed. Boxplots: median value depicted. Error bars: standard error. Statistical analysis: two-tailed unpaired t-test.

c. Percentage of variants among total variant-containing mRNA molecules expressed from genes of different lengths in wild type and *prde-1* mutants (variants identified by HaplotypeCaller and SnpEff). Q1-Q4: Genome divided into quantiles by gene length, as in Suppl. Fig 8b, c (gene lengths in bp). Y-axis: % variants. X-axis: Strain. Total variant containing sites=1315-1684. Boxplots: median value depicted. Error bars: standard error. Statistical analysis: two-tailed unpaired t-test.

d. Mean mismatch rates amongst variant-containing mRNA molecules (variants identified by HaplotypeCaller and SnpEff). Y-axis: Mean error rate (number of mismatch reads per total reads with variants). X-axis: Mismatch. Total variant containing sites=1315-1684. Approximately 3.71 x

bioRxiv preprint doi: https://doi.org/10.1101/2025.10.27.684940; this version posted December 10, 2025. The copyright holder for this preprint (which was not certified by peer review) is the author/funder, who has granted bioRxiv a license to display the preprint in perpetuity. It is made available under aCC-BY 4.0 International license.

10^7 individual mRNAs analyzed. Bars: mean value. Error bars: standard error. Statistical analysis: two-tailed unpaired t-test.

e. NMD-related gene expression levels in WT and *prde-1* animals on Ctrl (L4440) and *smg-2* RNAi (RT-qPCR: relative fold change). *Y39B6A.21* (Class III) expression values normalized to *eft-3* mRNA values and shown relative to wild-type Control RNAi-treated animals. Note *that smg-2 leads to a similar increase in mRNA levels in both wild-type* and *prde*-1. Y-axis: Relative RNA levels. X-axis: RNAi-treatment. Bars: mean value. Error bars: standard error. n = 4 biologically independent experiments. Statistical analysis: 2-way ANOVA.
